# *Cis*SERS: Customizable *In Silico* Sequence Evaluation for Restriction Sites

**DOI:** 10.1371/journal.pone.0152404

**Published:** 2016-04-12

**Authors:** Richard M. Sharpe, Tyson Koepke, Artemus Harper, John Grimes, Marco Galli, Mio Satoh-Cruz, Ananth Kalyanaraman, Katherine Evans, David Kramer, Amit Dhingra

**Affiliations:** 1 Molecular Plant Sciences Graduate Program, Washington State University, Pullman, Washington, United States of America; 2 Department of Horticulture, Washington State University, Pullman, Washington, United States of America; 3 School of Biological Sciences, Washington State University, Pullman, WA, Washington, United States of America; 4 Electrical Engineering and Computer Science, Washington State University, Pullman, Washington, United States of America; 5 MSU-DOE Plant Research Laboratory, Michigan State University, East Lansing, Michigan, United States of America; National Institute of Plant Genome Research, INDIA

## Abstract

High-throughput sequencing continues to produce an immense volume of information that is processed and assembled into mature sequence data. Data analysis tools are urgently needed that leverage the embedded DNA sequence polymorphisms and consequent changes to restriction sites or sequence motifs in a high-throughput manner to enable biological experimentation. C*is*SERS was developed as a standalone open source tool to analyze sequence datasets and provide biologists with individual or comparative genome organization information in terms of presence and frequency of patterns or motifs such as restriction enzymes. Predicted agarose gel visualization of the custom analyses results was also integrated to enhance the usefulness of the software. C*is*SERS offers several novel functionalities, such as handling of large and multiple datasets in parallel, multiple restriction enzyme site detection and custom motif detection features, which are seamlessly integrated with real time agarose gel visualization. Using a simple fasta-formatted file as input, *Cis*SERS utilizes the REBASE enzyme database. Results from *Cis*SERS enable the user to make decisions for designing genotyping by sequencing experiments, reduced representation sequencing, 3’UTR sequencing, and cleaved amplified polymorphic sequence (CAPS) molecular markers for large sample sets. C*is*SERS is a java based graphical user interface built around a perl backbone. Several of the applications of C*is*SERS including CAPS molecular marker development were successfully validated using wet-lab experimentation. Here, we present the tool C*is*SERS and results from *in-silico* and corresponding wet-lab analyses demonstrating that C*is*SERS is a technology platform solution that facilitates efficient data utilization in genomics and genetics studies.

## Introduction

High-throughput sequencing technologies continue to generate vast amounts of information. The DNA sequence information is processed for quality and assembled into contigs resulting in the generation of mature sequence data that is subsequently utilized by biologists in wet lab experiments. Availability of user-friendly computational tools specifically created to process large quantities of sequence information from multiple samples that catalyze the translation of these countless data into useful knowledge for addressing biological questions remains a bottleneck. Existing sequence data can be harnessed for nucleotide polymorphism information, ascertaining genetic diversity in a population, and reduced representation sequencing. The consequences of nucleotide polymorphisms are diverse. They might result in altering the phenotype if there is a change in an amino acid or alterations in the regulatory regions. Alternatively, these may be inconsequential mutations. Biologists endeavor to first identify and then utilize the polymorphic information to establish causal relationships between the genotype and the phenotype in genomics and genetics approaches.

There are several approaches in use that exploit nucleotide polymorphism information. On a global genomic scale, nucleotide polymorphism information is generated using whole genome sequencing, reduced representation sequencing [1–3], genotyping by sequencing [4,5], and SNP arrays [6,7]. Genotyping by sequencing and reduced representation sequencing both utilize restriction site information during sequencing library preparation for genomes [1, 2] and transcriptomes [3–5]. An example is Restriction-site Associated DNA (RAD) sequencing that enables identification of polymorphisms which are subsequently used as DNA markers for population analysis [6, 7]. Whole genome analysis of restriction sites can provide better information to help guide decisions for enzyme selection in digesting DNA for RAD sequencing libraries as well as BAC library production and sub-cloning. Transcriptome analysis via sequencing of 3’ untranslated regions (3’UTR) of cDNA libraries was enabled through the utilization of restriction enzyme digests in maize [3] and sweet cherry [4].

The afore-mentioned approaches are overkill when working with a single or a few hundred genes as is typical of most projects. For such focused applications, methods such as high resolution melting [4], allele specific PCR [8], single locus restriction fragment length polymorphisms (RFLP), amplified fragment length polymorphisms (AFLP) and cleaved amplified polymorphic sequences (CAPS) [9] are used. Of these, methods based on restriction enzyme digestion are relatively easy, widely used, reproducible, and cost-effective to perform and analyze due to the reduced need for specialized equipment and expertise. For genetics applications, restriction enzyme based molecular markers are commonly employed. Historically, these molecular markers have been developed either through trial and error or from polymorphisms among a limited set of individuals. With the availability of high-throughput sequencing technologies, the onus has shifted to identifying multiple, site-specific polymorphisms across large populations. CAPS markers, also known as PCR based Restriction Fragment Length Polymorphic markers (PCR-RFLP), are routinely used for mapping traits in populations and for enabling efficient breeding [9]. CAPS markers are popular due to their relatively low cost and general ease of use through the reliance on the common, simple molecular biology tools of PCR, enzymatic digests and gel electrophoresis [10]. These strategies, however, rely on *a priori* knowledge including the location and sequence of the restriction sites. In addition to polymorphism screening, many types of molecular biology methods utilize restriction site information and therefore require an efficient tool to analyze large sequence datasets.

Several current restriction site analysis tools, summarized in Table 1, have been designed to handle one or several small sequences for targeted analysis [11–17], not the thousands or millions that are now available with high-throughput sequencing. NEBcutter is the most generalized restriction site analysis tool with the others focusing primarily on developing DNA markers. NEBcutter provides useful functionalities from cloning analysis to gel predictions based on different types of gels [17]. Version 2.0 of this web tool, at its farthest limit, handles a single sequence of less than 300 kbases or an input file of a single sequence less than 1 Mbyte for analysis regarding any number of enzymes from the REBASE database [8]. The analysis pipeline enables most molecular functionalities but is not set up for high-throughput analysis nor multiple sequence analysis.

**Table 1:** Restriction Analysis Program Comparison. A comparison of some essential traits of C*is*SERS and other restriction site analysis programs highlights the advantages of C*is*SERS and some of the shared components with previously available tools. Many of these tools were designed for CAPS marker or derived CAPS (dCAPS) marker development and each has varying limitations.

Program	Web-based	Automated decision making	Primer design	Data input type	Enzyme list	Primary Functions	Predicted gel image	Major limitation	Citation
C*is*SERS	No	No	No	Fasta or multi-fasta	REBASE with customization	multiple digest site analyses	Yes	Processing resource limited	
dCAPS Finder 2.0	Yes	No	Yes	Requires 2 sequences	Preset database	CAPS or dCAPS design	No	2–60 base sequences	[11]
BlastDigester	Yes	Yes	Yes	multi-fasta	unknown	CAPS design	No	Limited by Blast	[12]
SNP2CAPS	No	Partial	No	Alignment file	User input	CAPS design	No	Multiple alignments	[13]
CapsID	Yes	Yes	Yes	Alignments	unknown	CAPS design	Yes	unknown	[14]
SNP cutter	Yes	no	Yes	dbSNP or preformatted SNP file	Premade lists using REBASE	CAPS or dCAPS design	No	Format dependent	[15]
SNP-RFLPing	Yes	partial	Yes	SNPs	REBASE	CAPS or dCAPS design	No	Human and Rat only	[16, 18]
NEBcutter	Yes	No	Yes	Fasta	REBASE	Comprehensive digest site analysis	Yes	Max file size 1Mb, max sequence length300kb	[17]

Like NEBcutter, most of the previous molecular marker developing tools are web-server based, limiting functionality for high-throughput analysis depending on the users’ internet connection and the tool’s server availability. The need to upload large datasets to these webserver-based programs can cause a significant bottleneck. Since the molecular marker development tools are mainly used to design CAPS markers, several of these tools have added primer design for amplification of a region around a polymorphism-modified restriction site. Additionally, several of these tools include levels of automated decision making that aids primer set and enzyme selection although this reduces user control and preferences.

Here we present a novel tool, C*is*SERS: Customizable *in silico* Sequence Evaluation for Restriction Sites that was developed to enable high-throughput analysis of mature multiple sequences for restriction sites with an embedded dynamic visualization functionality when identifying and selecting restriction enzymes or custom motifs for subsequent wet-lab applications. C*is*SERS output includes DNA digest information including an agarose gel prediction, to facilitate the user’s decision making process for selection of the most appropriate enzyme(s) for the project application. Unlike any other program in its class, C*is*SERS allows for custom motif detection to identify conserved sites, such as *cis*-acting elements or trans-acting protein binding sites, among all sequences and it even predicts amplicon lengths when oligonucleotides are input as custom motifs. For user convenience and project efficiency, C*is*SERS retains project files to provide easy access to the predicted cut site information to reduce time when the project requires iterative interactions, additional analysis or when two projects require comparison. In summary, C*is*SERS is expected to bridge the gap between sequence acquisition and implementation of diverse wet-lab approaches in order to address biological questions.

## Material and Methods

### Overview of C*is*SERS

**Fig 1:**
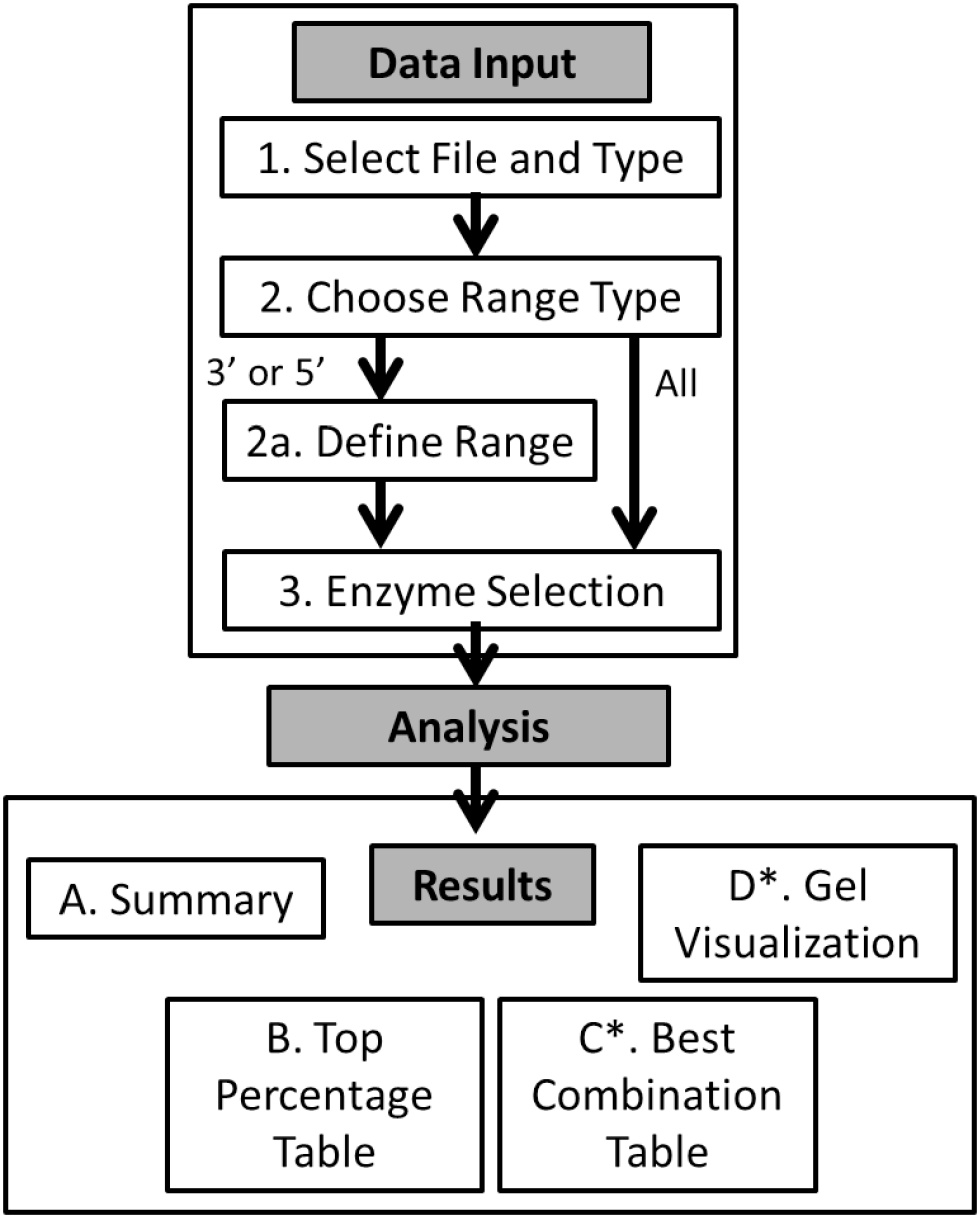
C*is*SERS experimental flow chart. A graphical depiction of the three phases of the C*is*SERS process and their subsections.

C*is*SERS was developed as a standalone program to provide processing of fasta files for restriction site and custom motif analyses generating tables of counts and predicted gel image as outputs. C*is*SERS is a java based graphical user interface built around a perl backbone presented in a standalone java execution file. C*is*SERS requires a onetime download of latest release of Java Runtime Environment (JRE) and Perl and, the C*is*SERS java archive (jar). While there are no known platform dependencies, the program runs without any problems in JRE version 7 on all operating systems. Using hundreds of thousands or millions of sequences or a single sequence in a single fasta file, selected motifs are identified, displayed and analyzed through a java based graphic user interface (GUI). The fundamental string matching functionality embedded in PERL enables motif identification. After analysis is complete, the outputs are displayed in the program including tables describing the cut counts and locations for each restriction site and dynamically created predicted gel images. Fig 1 provides a graphical overview of the C*is*SERS workflow. The program (CisSERS.jar), User manual for the C*is*SERS program and C*is*SERS Overview and Usage graphics can be found in S1, S2 and S3 Files. The CisSERS source code is available in S5 File.

## General Workflow

### User Input

For data entry into C*is*SERS, the user selects the fasta-formatted DNA sequence file for processing using a standard folder/file selection. The file is analyzed to verify that it is in the proper format and C*is*SERS will warn the user if irregularities are identified once it is started. For transcriptome analysis approaches where poly-A trimming and 5’ to 3’ orientation is desired, selection of the ‘Sequences have Poly-A tails’ check box enables this pre-processing (refer to the User’s Manual for all program option locations). If poly-A is selected, identification and subsequent trimming of the poly-A is completed. Sequences without a recognized poly-A sequence are placed in a separate file and are not processed further. The user can also determine how far into a sequence the poly-A tail search will continue. Poly-A tail identification, based on the approach found in the EMBOSS program ‘trimest’ [19], occurs if 4 or more consecutive A’s are found within the specified range. The poly-A tail search will be extended until more than 1 non-A character is identified. If a search limit has been specified, the algorithm will extend the poly-A tail beyond the range to most effectively trim the sequence. While the poly-A identification is running, the algorithm is also applied to identify poly-T heads which are then reverse complemented and placed in the proper 5’ to 3’orientation for all further analyses. The trimmed files, containing all sequences in the 5’ to 3’ orientation, and non-trimmed files generated during this process are saved for further use if desired. Without the Poly-A selection, all reads are processed in their input orientation and must be oriented in the 5’ to 3’ direction for use in non-transcriptomic approaches.

The second step of user input is to choose the desired ‘cut site’ area. The user can choose the area of predicted cut sites from either end or along the entire length of the sequence. The cut sites are predicted using pattern or expression matching functionality inherent in Perl. Fasta files provided by the user are opened and each sequence line is read as a variable to which the master list of enzymes available from the REbase database [17] are identified using the regular expression matching functionality. The 3’ and 5’ options enable the selection of a range of the sequence to be analyzed from the desired end. The user enters this information by dragging the slider or entering the exact positional information in the dialog boxes below the slider diagram.

The third and final input required prior to initiating analysis by C*is*SERS is the enzyme selection. The master list of enzymes is retrieved from the REbase database [17] and can be periodically updated by the user from within C*is*SERS if the host computer has internet access. Enzyme selection can be done through a checkbox tree, a filtering window, or through a name/site search. This allows the user to choose enzymes meeting a number of criteria including custom lists. User defined enzymes and recognition sequences can also be added to the database if desired. The options menu also allows the user to select which outputs are desired prior to starting the analysis.

### Analysis

After all user inputs are entered, selecting ‘Run’ at the bottom of the screen begins the analysis process. During this time, a ‘processing’ tab is shown depicting the progress of the analysis through each stage. Once the analysis is complete, this tab will disappear and ‘Summary’, ‘Best’ and ‘Top’ tabs will appear when the ‘Sequences have Poly-A tails’ box is selected; when the ‘Sequences have Poly-A tails’ box is not selected, an additional ‘Gel Visualization’ tab appears.

### Results/Outputs

The primary outputs of C*is*SERS are: Summary, Best, Top tables and Predicted Gel Visualization as mentioned previously. Each of these outputs is shown on an individual tab and can be saved individually by C*is*SERS. Projects can be saved and reloaded to eliminate the need to reprocess the data.

The ‘Summary’ table displays the total results for each enzyme. This includes the total number of sequences which contain at least one occurrence of that enzyme’s recognition sequence, the total number of cut sites in the fasta file and the percentage of sequences that are cut by this enzyme. The ‘Best’ table is used for finding combinations of enzymes that cut the most sequences. For 3’UTR sequencing, a minimum number of enzymes that cut nearly every transcript in the desired range are ideal. Using a greedy approach, where sequences cut by the best enzyme are removed and the next best enzyme is identified, the enzymes are listed with the combined percentage of sequences cut. Additionally, since there are minimum lengths for processing DNA through some applications, the 'Best' table also displays cut sites in the 'Pre-cut Area', the area between the beginning of the sequence and the beginning of the desired cut area. The last table produced is the ‘Top’ table. This table is a filtered version of the ‘Summary’ table that only displays the enzymes cutting a minimum percentage of the sequences. This setting defaults to 95% and is adjustable through the options menu. These data tables combine to inform the user of the restriction site information necessary to enable many biological approaches.

### Cut Site Identification and Gel Visualization

Basic functionalities of C*is*SERS were tested by evaluating its ability to properly identify restriction sites or motifs using expression matching functionality in Perl and produce a predicted gel image on datasets constructed with known restriction enzyme recognition site sequence inserted into the middle of poly-T sequences resulting in unique sequences with a final length of 60 bases. The set of formulas C*is*SERS uses to produce fragment size to distance relationships were derived and modified from previous reports [9–12]. Briefly, the published formulae describe fragment to fragment interaction in a resistive manner and thus different sized DNA fragments have a size specific differential resistance associated with them while passing through a matrix of agarose fragments. These size specific differential resistance associations were applied to Ohm’s Law, which states that current multiplied by resistance equals voltage (IR = V), and using a voltage constant of 70 VDC to obtain size specific current values. The size specific current values were extrapolated as velocity values and, utilizing a time constant of 45 minutes, distance relationships were obtained using the velocity multiplied by time equals distance equation (VT = D).

### Custom Motif Identification

The ability to examine a certain set of sequences for shared motifs has vast applications. These motifs could be cis-acting transcriptional or translational regulators that impact gene expression and ultimately the phenotype of the organism. C*is*SERS offers this distinctive feature to search for custom motifs enabling such analyses. Custom motifs are input by the user and added to the Cut Sequence list. They are selected via the check box option and are identified in the sequence of interest based on the regular expression matching functionality in Perl.

#### Demonstration case 1

The motif detection feature of C*is*SERS was validated by analyzing the *Nostoc* sp. PCC 7107 genome. *Nostoc* is a commonly used nitrogen fixing microbe used in undergraduate curriculum and possesses a circular genome. The *Nostoc* genes may start with a Pribnow box or an alternate AT rich version that corresponds to the IUPAC code WWWWWW motif where W corresponds to either an A or a T. This motif is generally located between the -4 and -12 position relative to the ATG start codon. The number of bases between the motif and the start codon was varied by inserting 4–12 N’s, WWWWWWN_4-12_ yielding the motifs shown in Table 2. Annotated genes with these motifs were identified and analyzed for the subset of genes that contains all 9 motifs in the transcriptional initiation area.

**Table 2:** AT rich transcription initiation motifs, total motif sites and number of identified motifs associated with annotated gene transcription initiation areas.

Motif Name	Motif	Total number of Identified sites	Total number of gene Identified sites
weakTss-10-4N	WWWWWWNNNNATG	4847	227
weakTss-10-5N	WWWWWWNNNNNATG	4715	220
weakTss-10-6N	WWWWWWNNNNNNATG	4759	206
weakTss-10-7N	WWWWWWNNNNNNNATG	4769	197
weakTss-10-8N	WWWWWWNNNNNNNNATG	4585	192
weakTss-10-9N	WWWWWWNNNNNNNNNATG	4544	196
weakTss-10-10N	WWWWWWNNNNNNNNNNATG	4669	208
weakTss-10-11N	WWWWWWNNNNNNNNNNNATG	4565	212
weakTss-10-12N	WWWWWWNNNNNNNNNNNNATG	4488	217

#### Demonstration case 2

Typically polyadenylation of mRNA 3’ untranslated region (UTR) requires a polyadenylation initiation site that facilitates the binding of the cleavage/polyadenylation specificity factor (CPSF) complex which cleaves and polyadenylates the 3’ end. The majority of eukaryotic mRNA transcripts possess a polyadenine (polyA) tract on the 3’ end of the transcript. The polyA tract has been implicated in regulation of mRNA degradation and translation [13]. Alternative processing of mRNA transcripts can lead to different isoforms of a gene either performing alternative functions, differential regulation in a pathway or gene auto-regulation or non-functionality of a gene product [14]. Among the different forms of alternative processing is the existence of premature polyadenylation of a transcript. C*is*SERS was used to find the predicted polyA initiation sites within 300 bases of the terminal 3’ reported base for each of the ESTs contained in the TAIR ATH_cDNA_EST_sequences_FASTA file for transcripts that could support this form of alternative mRNA processing. Due to the large memory requirements to process the complete file, the ATH_cDNA_EST_sequences_FASTA file was subdivided into 37 datasets, 36 files containing 50,000 fasta sequences and 1 containing 16,638 sequences, for processing by C*is*SERS and the individual subset results were collated. The human canonical AATAAA polyA initiation recognition site, as well as previously identified eukaryotic polyA initiation recognition sites [15], were used as input motifs to identify polyA initiation recognition sites present in the expressed sequence tags of the Arabidopsis dataset (Table 3).

**Table 3:** C*is*SERS summary table of analysis of potential polyA initiation sites from 1,816,638 Arabidopsis cDNAs of the ATH_cDNA_EST_sequences_FASTA dataset from the ftp://ftp.arabidopsis.org/home/tair/Sequences/ website.

PolyA Motif Name	Motif	Number of Motifs Found in the EST dataset	Percentage of ESTs Motif Found
PolyA_Init_1	AAAAAG	358,447	19.73%
PolyA_Init_10	GATAAA	117,536	6.47%
PolyA_Init_11	TATAAA	169,316	9.32%
PolyA_Init_2	AAGAAA	446,967	24.60%
PolyA_Init_3	AATACA	152,030	8.37%
PolyA_Init_4	AATAGA	152,717	8.41%
PolyA_Init_5	AATATA	142,002	7.82%
PolyA_Init_6	ACTAAA	224,897	12.38%
PolyA_Init_7	AGTAAA	166,592	9.17%
PolyA_Init_8	ATTAAA	174,486	9.60%
PolyA_Init_9	CATAAA	135,197	7.44%
PolyA_Init_canonical	AATAAA	246,069	13.55%

### CAPS marker development

CAPS marker development is an important feature of C*is*SERS that was tested to verify the utility of this function. CAPS markers were developed for diploid and polyploid species by analyzing the input sequences in demonstration cases 1 and 2 with C*is*SERS using all restriction enzymes. The restriction digestion results were visualized using the virtual gel output and digestions patterns were visually parsed for discernible differences in sizes of the digested DNA fragments with each restriction enzyme. A single restriction enzyme or a combination of preferably two enzymes can be used to obtain different restriction digestion pattern from similar sequences with embedded polymorphisms, thus resulting in the development of a CAPS marker. As NEBcutter V2.0 does not have a method for analyzing multiple sequences in parallel, which is critical for enzyme comparison, only C*is*SERS-identified enzymes for CAPS markers were analyzed in subsequent biological experiments.

#### Demonstration case 1

**Fig 2:**
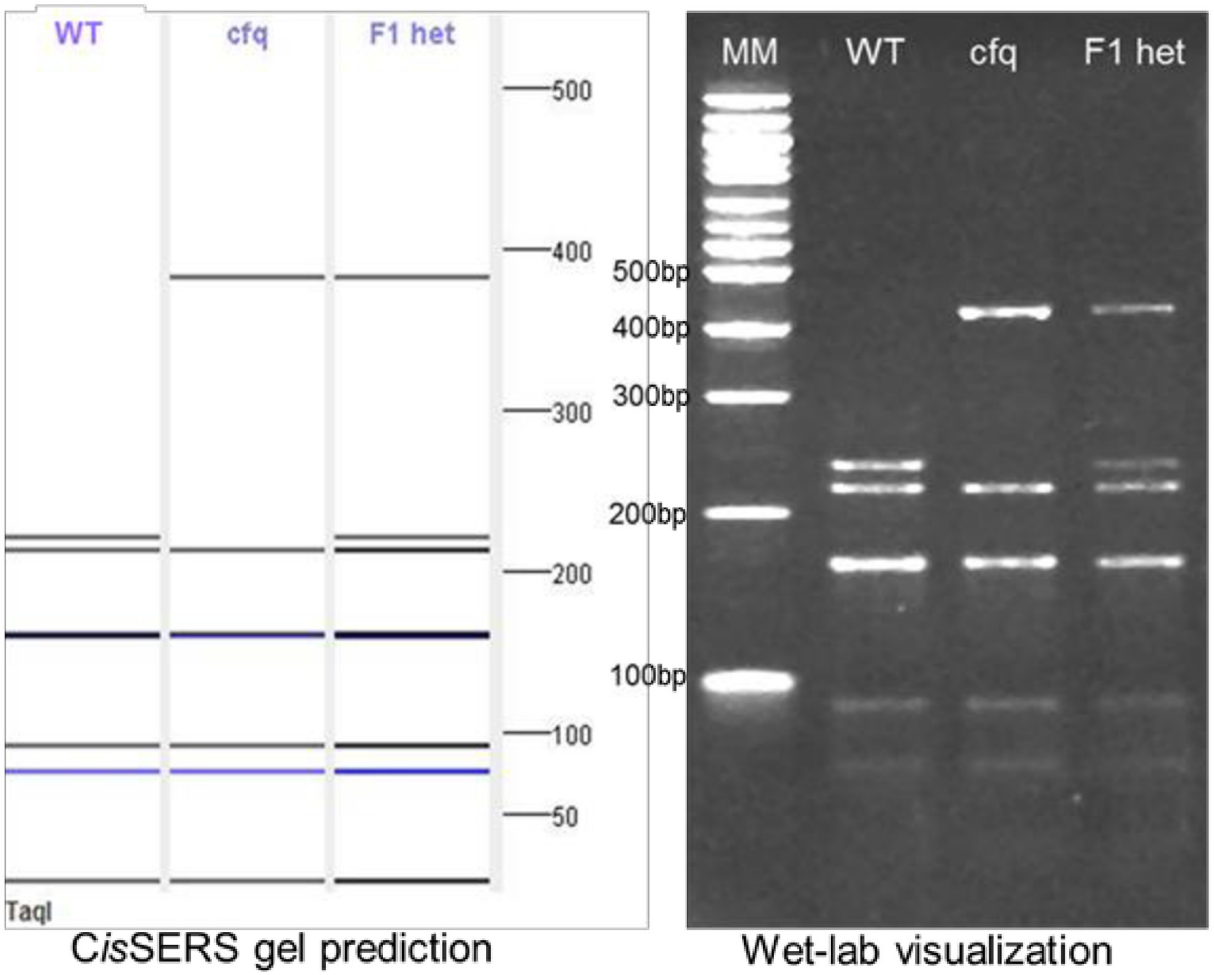
Demonstration case 1: C*is*SERS predicted gel image vs. wet-lab gel visualization test with the *Arabidopsis* ATPC1, cfq mutant sequences. The two were linked to create the “F1 het” lane image while the F1 heterozygous plant DNA was analyzed and labeled “F1 het” in the wet-lab validation image. The banding patterns of all three samples of the C*is*SERS prediction match the wet-lab validation confirming the effectiveness of C*is*SERS to determine effective CAPS marker enzymes.

ATPC1 is one of the two nuclear encoded genes in *Arabidopsis* for the γ subunit of the chloroplast ATP synthase [17]. The coupling factor quick recovery (*cfq*) mutant of *Arabidopsis* was identified as a point mutation in the ATPC1 gene and reduces overall photosynthetic capabilities [18]. The sequences of wild type *Arabidopsis* ATPC1 and the cfq mutant form were processed through C*is*SERS. The purpose for C*is*SERS analysis was to identify at least one restriction enzyme displaying significant visual differences for use as a CAPS marker to enable population screening. DNA was extracted from wild type, cfq mutant, and heterozygous *Arabidopsis* plants and the ATPC1 gene was amplified. The product was then digested with TaqI identified by C*is*SERS for 1 hour at 65°C and electrophoresed on a 10% TBE-Acrylamide gel (Bio-Rad), stained with ethidium bromide, and visualized (Fig 2).

#### Demonstration case 2

**Fig 3:**
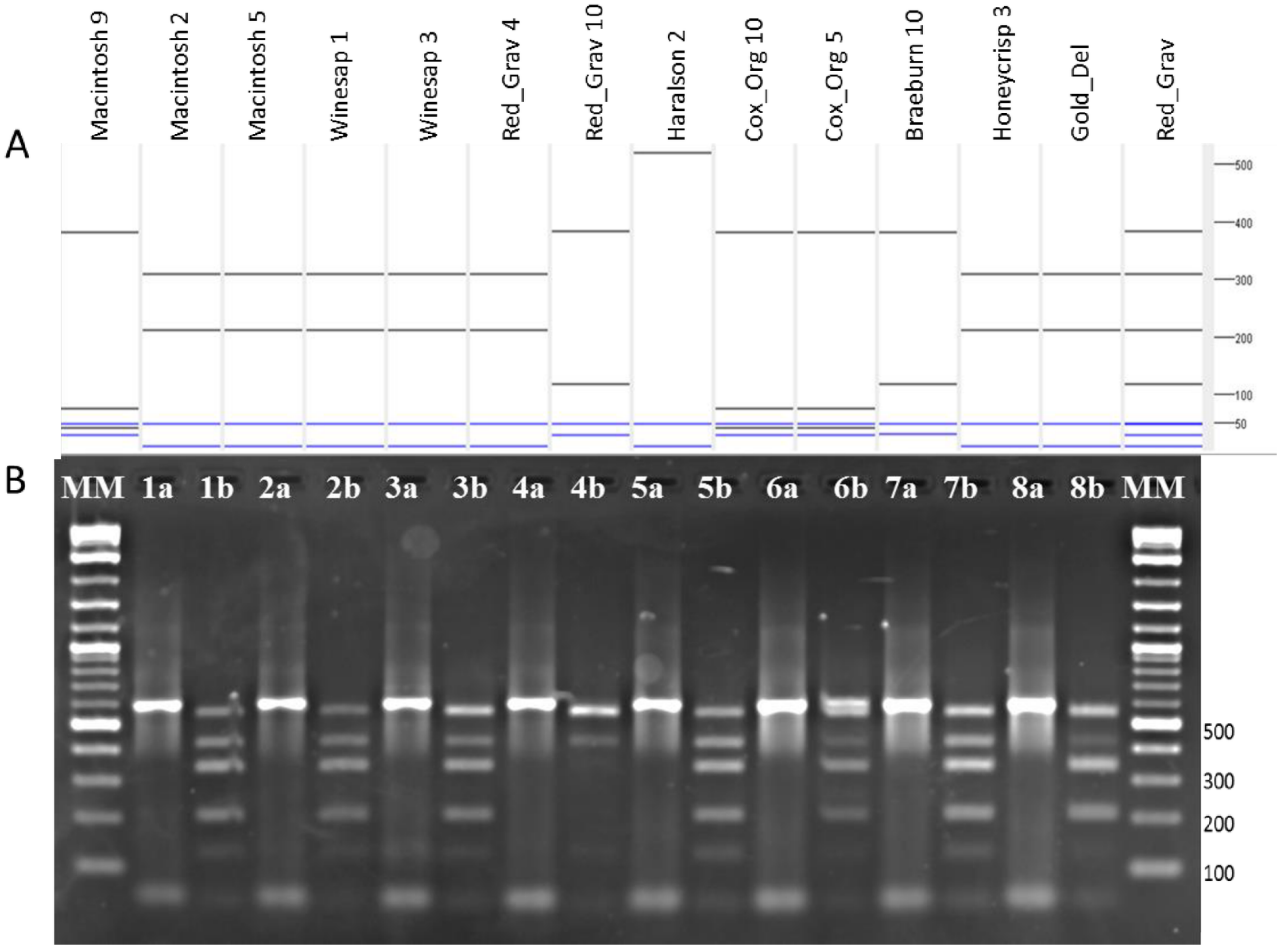
Demonstration case 2: C*is*SERS predicted gel image vs. wet-lab gel visualization test with the Galli sequences. **A**. C*is*SERS predicted gel image of 12 identified alleles from 8 apple cultivar’s cDNA clones, and 2 linked gel images (Gold_Del, and Red_Grav). **B**. Wet-lab electrophoresed gel image of amplified products (#a) and corresponding restriction digest (#b); 1. ‘Macintosh’, 2. ‘Winesap’, 3. ‘Red Gravenstein’, 4. ‘Haralson’, 5. ‘Cox’s Orange Pippin’, 6. ‘Braeburn’, 7. ‘HoneyCrisp’, 8. ‘Golden Delicious’, MM = 100bp DNA molecular marker. Analysis of the individual cultivars (A: Haralson 2 and B: 4b) suggest that ‘Haralson’ is homozygous for the sequenced allele; (A: Macintosh 9, Macintosh 2, Macintosh 5 and B: 1b) indicates that each allele present in ‘Macintosh’ is not yet sequenced; and (A: Cox_Org 10, Cox_Org 5 and B: 5b) also indicates that each allele of ‘Cox’s Orange Pippin’ has not yet been sequenced.

A gene putatively involved in bitter-pit disorder of apple was identified in previous work (Schaeffer and Dhingra, unpublished). Apple is an allotetraploid with a recently published genome [20]. Cloning and sequencing of this gene from eight apple cultivars varying in degree of disorder prevalence was completed. These sequences were then analyzed with C*is*SERS to identify an enzyme which separates the major alleles present in these cultivars. Wet-lab evaluation was performed by amplifying the region and digesting with Cac8I for 3 hours at 37°C. The resulting DNA fragments were electrophoresed on a 2% agarose gel and visualized (Fig 3).

### High-Throughput Analysis

C*is*SERS has the unique capability of analyzing large datasets rapidly, which is a major upgrade compared to other restriction site analysis programs. To demonstrate this functionality, the *Arabidopsis thaliana* EST cDNA dataset was downloaded from the TAIR website and processed with C*is*SERS. 1,816,638 sequences with an average length of 321 bases, longest sequence of 2,883 bases and the shortest EST of 1 base. To limit the output for Table 4 and to emphasize the customization of enzyme selection, the 6 base cutter restriction enzyme set was used to process the entire dataset.

**Table 4:** Restriction enzymes identified with the highest predicted cut site percentages of the Arabidopsis cDNA dataset.

Motif	Cut Seq	# Seqs Cut	# Total Cuts	Percent of Total Sequences Cut	Motif	Cut Seq	# Seqs Cut	# Total Cuts	Percent of Total Sequences Cut
AatII*	GACGTC	66,910	70,203	4.37%	BsgI	GTGCAG	133,382	145,615	8.72%
AccI*	GTMKAC	607,694	884,956	39.73%	BsiWI*	CGTACG	35,290	36,309	2.31%
AccIII*	TCCGGA	90,096	95,917	5.89%	Bso31I*	GGTCTC	258,799	300,090	16.92%
AclI*	AACGTT	128,216	137,480	8.38%	Bsp1286I*	GDGCHC	745,627	1,233,399	48.74%
AcsI*	RAATTY	890,483	1,555,052	58.21%	Bsp1407I*	TGTACA	34,156	37,236	2.23%
AcuI*	CTGAAG	330,051	398,938	21.58%	BspHI*	TCATGA	43,037	46,935	2.81%
AflII*	CTTAAG	132,971	141,810	8.69%	BspLI*	GGNNCC	336,757	762,139	22.01%
AflIII	ACRYGT	460,914	617,710	30.13%	BssECI*	CCNNGG	563,045	1,374,552	36.81%
ApaI*	GGGCCC	21,606	23,212	1.41%	BssHII*	GCGCGC	8,030	8,266	0.52%
ApaLI*	GTGCAC	47,527	49,058	3.11%	Bst1107I*	GTATAC	54,764	56,807	3.58%
AvaI*	CYCGRG	462,908	609,100	30.26%	Bst6I*	CTCTTC	657,095	1,014,786	42.96%
BaeGI*	GKGCMC	76,258	93,761	4.99%	BstBAI*	YACGTR	412,601	528,315	26.97%
BalI*	TGGCCA	111,189	119,300	7.27%	BstC8I*	GCNNGC	451,250	715,776	29.50%
BamHI	GGATCC	113,866	122,596	7.44%	BstDSI*	CCRYGG	458,532	593,070	29.98%
BanI*	GGYRCC	458,532	593,070	29.98%	BstMCI*	CGRYCG	336,221	417,841	21.98%
BanII*	GRGCYC	462,908	609,100	30.26%	BstSFI*	CTRYAG	191,390	266,617	12.51%
Bbv12I*	GWGCWC	565,452	815,716	36.96%	BstV2I*	GAAGAC	412,497	525,230	26.97%
BclI*	TGATCA	205,509	231,475	13.43%	BstX2I*	RGATCY	665,518	1,048,893	43.51%
BfuAI*	ACCTGC	74,558	81,931	4.87%	BsuI*	GTATCC	91,341	100,997	5.97%
BglII	AGATCT	235,829	269,983	15.42%	BtgZI	GCGATG	143,795	155,002	9.40%
BlnI*	CCTAGG	36,333	37,306	2.38%	BtsI	GCAGTG	170,608	190,109	11.15%
BmuI*	ACTGGG	68,520	73,919	4.48%	Cfr10I*	RCCGGY	447,942	605,631	29.28%
BpmI*	CTGGAG	238,862	274,263	15.61%	ClaI*	ATCGAT	147,263	161,798	9.63%
BpuEI	CTTGAG	344,371	414,909	22.51%	DraI	TTTAAA	221,889	257,617	14.51%
BsaWI	WCCGGW	610,391	902,258	39.90%	EaeI*	YGGCCR	447,942	605,631	29.28%
Bse3DI*	GCAATG	213,691	236,982	13.97%	EciI	GGCGGA	150,946	170,687	9.87%
BseRI	GAGGAG	479,092	689,960	31.32%	Eco47III*	AGCGCT	33,781	35,254	2.21%
Eco52I*	CGGCCG	39,284	42,514	2.57%	NspV*	TTCGAA	93,554	100,814	6.12%
Eco57MI	CTGRAG	495,072	672,679	32.36%	PciI*	ACATGT	132,704	143,845	8.68%
EcoRI	GAATTC	132,547	142,270	8.66%	PinAI*	ACCGGT	109,189	118,721	7.14%
EcoRV*	GATATC	140,416	152,012	9.18%	PsiI*	TTATAA	152,592	167,870	9.98%
Esp3I*	CGTCTC	135,776	159,958	8.88%	PspCI*	CACGTG	42,489	46,326	2.78%
FspI*	TGCGCA	33,983	34,697	2.22%	PstI	CTGCAG	130,827	143,216	8.55%
HaeII*	RGCGCY	261,757	314,036	17.11%	PvuI*	CGATCG	59,418	62,867	3.88%
Hin1I*	GRCGYC	418,344	541,230	27.35%	PvuII	CAGCTG	156,402	173,376	10.22%
HincII*	GTYRAC	652,459	970,197	42.65%	SacI*	GAGCTC	180,014	198,914	11.77%
HindIII	AAGCTT	353,375	429,786	23.10%	SacII*	CCGCGG	34,831	36,071	2.28%
HpaI*	GTTAAC	103,809	109,093	6.79%	SalI	GTCGAC	72,616	77,632	4.75%
Hpy166II*	GTNNAC	660,473	2,352,103	43.18%	ScaI*	AGTACT	110,216	115,920	7.21%
Hpy188III	TCNNGA	1,310,936	4,488,839	85.70%	SmaI*	CCCGGG	37,962	39,858	2.48%
KpnI*	GGTACC	59,800	61,722	3.91%	SmlI*	CTYRAG	779,347	1,353,965	50.95%
MluI	ACGCGT	29,567	30,996	1.93%	SnaBI*	TACGTA	43,318	44,707	2.83%
MmeI	TCCRAC	454,882	595,189	29.74%	SpeI*	ACTAGT	63,608	66,363	4.16%
MspA1I	CMGCKG	468,051	617,393	30.60%	SphI*	GCATGC	51,290	53,131	3.35%
MunI*	CAATTG	122,886	130,536	8.03%	SspI	AATATT	162,718	182,345	10.64%
NaeI*	GCCGGC	48,336	50,517	3.16%	StuI*	AGGCCT	66,041	68,945	4.32%
NarI*	GGCGCC	39,029	39,944	2.55%	StyI*	CCWWGG	652,286	978,963	42.64%
NcoI*	CCATGG	180,000	196,511	11.77%	TatI	WGTACW	702,344	1,105,498	45.91%
NdeI*	CATATG	107,887	114,961	7.05%	TsoI	TARCCA	439,177	562,079	28.71%
NheI*	GCTAGC	61,076	63,117	3.99%	VspI*	ATTAAT	131,420	145,945	8.59%
NmeAIII	GCCGAG	146,659	161,103	9.59%	XbaI	TCTAGA	109,179	116,409	7.14%
NruI*	TCGCGA	40,330	42,131	2.64%	XhoI*	CTCGAG	120,878	131,508	7.90%
NsiI*	ATGCAT	119,796	128,076	7.83%					
NspI*	RCATGY	424,159	561,111	27.73%					

Enzymes with a * symbol at the end represent multiple enzymes which all have the same recognition site.

## Results

### Custom Motif Identification

#### Demonstration case 1

While there are few canonical transcriptional start sites (TATAAT sites) associated with *Nostoc* genes (Nos7107_0081 hypothetical protein and Nos7101_1087 group 1 glycosyl transferase on the forward strand and Nos7107_3714 hypothetical protein on the reverse strand), the distinctive functionality of C*is*SERS can be used with the degenerate nucleotide base codes to increase the identification of possible *cis*-element Pribnow box motif variations. A total of 41,941 potential AT rich transcriptional start sites are present in the *Nostoc* genome on the forward strand when the 6 base AT rich site is moved through the -7 to -16 ATG upstream area (Table 2). Of the 41,941 motifs found, 1,875 corresponded to the annotated transcription initiation site area. 12 of the 1,875 identified annotated genes were identified to contain all 9 possible 6-base AT rich potential transcriptional start site motifs.

#### Demonstration case 2

Of the 1,816,638 ESTs in the *Arabidopsis* dataset, 36.86% of the ESTs possess multiple predicted polyA initiation recognition sites within 300 bases of the terminal 3’ base. The number of ESTs and the percentage of the polyA initiation site motif in comparison to the total number of ESTs can be found in Table 3. The recognized polyA initiation motifs ranged from a low of 6.47% to a high of 24.60% of the EST database. An increase of 36.86% of recognized motifs in comparison to the number of ESTs indicate there are ESTs with multiple polyA initiation recognition sites. While C*is*SERS may not be capable to differentiate which of these sites is utilized *in vivo* the information C*is*SERS provides enables an additional level of focus to which ESTs may possess multiple polyA initiation sites as well as which ESTs could be transcriptionally regulated due to premature polyA extension of the transcript.

### CAPS marker development

#### Demonstration case 1

To screen a population of *Arabidopsis* for the *cfq* mutation, the wild type (WT) and mutant sequences (cfq) were processed using C*is*SERS. The analysis revealed TaqI as an enzyme that generates clear differences due to the point mutation (Fig 2a). Biological examination of the wild type, cfq mutant, and F1 heterozygous plants was conducted through digestion of the amplified ATPC1 gene product. Visualization of the digestion pattern was completed with a 10% polyacrylamide gel (Fig 2b). The banding pattern in the biological gel matches the predicted gel image produced by C*is*SERS and verified the utility of this tool for an enzyme selection for CAPS analysis of this mutation. This CAPS marker is currently being deployed for screening of F1 and F2 plants and confirming the phenotypic observations demonstrating the utility of C*is*SERS in enabling genetics research (Cruz and Kramer, unpublished). However, *Arabidopsis* represents a diploid demonstration case with a very well defined genome. Such analyses can get complicated in the case of a sample with a higher ploidy as illustrated in the next demonstration case.

#### Demonstration case 2

Sequencing of a selected Md*pbag* (*Malus x domestica* putative bitter pit associate gene) gene from eight apple cultivars yielded fifteen sequences which were manually trimmed to remove plasmid and primer sequences (S4 File). Upon C*is*SERS evaluation, Cac8I was chosen due to its potential to differentiate five of the alleles across these eight cultivars (Fig 3A). The wet-lab gel (Fig 3B) and the predicted gel image agreed with only three of the eight digested samples. These differences likely indicate presence of additional alleles which are not expected to be represented by the draft apple genome [[20]]. However, further analysis provides a resolution to some of the differences. C*is*SERS predicts the restriction digest banding pattern only of the sequence used as input, and, for heterozygous organisms, the banding pattern for a restriction enzyme digest will be representative of all alleles as seen for the ‘Macintosh’ predicted digest and the actual digest (Fig 3A first 3 lanes and Fig 3B lane 1b). To resolve the latter difficulty, C*is*SERS has the distinctive functionality to link two or more sequences together to provide more accurate predictive gel visualization. This is demonstrated with the ‘Red Gravenstein’ samples in Fig 3A where the ‘Red_Grav4’ and ‘Red_Grav10’ alleles were linked to produce the ‘Red Grav’ composite. The linked predicted gel visualization matches the wet-lab gel from ‘Red Gravenstein’ in lane 3b of Fig 3B. The predicted gel visualization for cloned sequences from ‘Haralson’ and the actual gel demonstrates the possibility that the ‘Haralson’ cultivar is homozygous for this allele and further in-depth investigation is warranted. The wet-lab gel digests demonstrate the remaining cultivars display a combination of two alleles indicating these are heterozygous and require sequencing of additional clones to capture the other allele. Subsequent clone selection and sequencing for these cultivars captured additional alleles indicated from this analysis (data not shown). This process illustrated a case where C*is*SERS output identified an enzyme that was not effective at resolving all the alleles in a species with a complex genome and provided impetus to pursue additional experimentation which resulted in identification of additional alleles. Also, application of C*is*SERS in complex genomes with draft genome information can enable identification of areas that may have potential sequence inaccuracies.

### High-Throughput Analyses

#### Demonstration Case

Analysis of 78,096 cDNA sequences from *Arabidopsis* with 41 restriction enzymes through C*is*SERS produced the summary in Table 4. The individual enzymes’ restriction sites were identified in a range of 1.63% to 81.28% of the total number of sequences. BssHII restriction sites represent the least number of sites in the 78,096 cDNA dataset and the largest number of restriction sites for a single enzyme was found for Bst6I at 168,429 sites. These results demonstrate the unique capability of C*is*SERS to process large datasets for guiding enzyme selection decisions for global applications such as reduced representation sequencing.

## Discussion

The results demonstrate the effectiveness and multiple distinctive functionalities of C*is*SERS in analyzing mature sequence data. Identifying enzymes for CAPS markers was highly effective in the *cfq* example. Interestingly, in the case of Md*pbag* sequences, the resulting information about the probable heterozygosity of the locus was critical and resulted in further investigation revealing the presence of additional alleles, thus guiding further wet lab research. The highly customizable and diverse motif detection functionality resulted in the identification of potential AT rich transcriptional start sites in the *Nostoc* genome. The versatility of C*is*SERS is evident by using the motif identification feature to predict prokaryotic promoter architecture and eukaryotic poly-adenylation (polyA) initiation recognition sites. Canonical as well as non-canonical Pribnow and polyA initiation site *cis*-element motifs in the sequence upstream of the coding sequence areas of *Nostoc sp*. *PCC 7107*, NC_019676.1, and sequence upstream of the 3’ UTR area of *Arabidopsis thaliana* was searched and potential *cis*-elements were identified to facilitate future investigation. Lastly, the high-throughput analysis capabilities of C*is*SERS were demonstrated. Analyzing entire transcriptomes or genomes enables data-guided decision making for subsequent restriction enzyme based experimentation. High-throughput sequencing technologies are expensive and experimental design is a major component prior to sequencing. Based on the effective identification of restriction sites in standard and custom sequences, the identification of enzymes for reduced representation sequencing is also expected to be accurate and help ensure quality experimental design prior to sequencing. Combined, these experiments confirm the biological applicability of C*is*SERS as a highly effective addition to researcher’s toolkits.

### Limitations and Future Improvements

C*is*SERS is a comprehensive and useful tool as demonstrated in previous sections. Extremely large datasets may require higher amounts of RAM or lengthy run times when processing all enzymes and gel visualization of these datasets may cause noticeable computer lag. These limitations are overcome by increasing RAM and computer processing speeds but can also be alleviated by decreasing the amount of input sequences or the number of enzymes being processed. C*is*SERS relies on base sequence as supplied in the fasta format which does not hold any type of sequence specific methylation data and as such any methylation susceptible restriction enzyme site identification by C*is*SERS would have to be scrutinized. At this point, methylation identification is simply based on any change in function of the restriction enzyme by methylation including: requiring methylation, requiring no-methylation and any partial specificity. Currently, each of these enzymes must be further evaluated by the user to make sure the chosen enzyme fits their project and the type of DNA they are processing.

## Conclusions

As a tool developed to facilitate biological approaches, C*is*SERS enables the identification of restriction sites and custom motifs in large mature multi-sequence data files. Genotyping by sequencing and reduced representation sequencing approaches commonly utilize a restriction enzyme and C*is*SERS provides an efficient platform that will aid in the decision making process for users to determine the number of sites across the genome or transcriptome of interest. This is expected to facilitate guided development and deployment of CAPS markers for breeding and restriction enzyme selection for mutation identification that leverage the polymorphisms present in populations. Additionally, the custom motif functionality provides a convenient tool to query assembled genomes and transcriptome datasets for regions of biological interest. Overall, C*is*SERS is a standalone, open source front end tool for efficient and prudent utilization of next-generation sequencing data as the science begins to shift focus from how much data can be obtained to how we best utilize these data.

## Supporting Information

S1 File C*is*SERS program in .jar format available for download. (JAR)

S2 File C*is*SERS User Manual. (PDF)

S3 File C*is*SERS Overview and Usage Document. (PDF)

S4 File Sequences used for CAPS marker development in this study. (PDF)

S5 File CisSERS source code. (ZIP)
