# Supplementary material for: CisSERS: Customizable In Silico Sequence Evaluation for Restriction Sites
Source: PLoS One. 2016 Apr 12;11(4):e0152404. doi: 10.1371/journal.pone.0152404 (PMC4829253; doi:10.1371/journal.pone.0152404)
Supplement: S2 File — (PDF) [file pone.0152404.s002.pdf]

C*is*SERS: Customizable *in silico* Sequence Evaluation for Restriction Sites

Richard M Sharpe, Tyson Koepke, Artemus Harper, John Grimes, Marco Galli, Mio Cruz, Anantharaman Kalyanaraman, Katherine Evans, David Kramer, and Amit Dhingra

Washington State University
Copyright 2012

CisSERS User Manual

Table of Contents

Introduction....3
Usage diagram....4
Prerequisites....5
Project setup....6
- Select Input File....7
- Decide if Poly-A tails are present....7

Decide Cut site....7
Select Desired Enzymes....8
- Manual Enzyme selection....8
- Selecting Enzyme by Criteria....8
  - Cut Type....8
  - Recognition Site length....9
  - Methylation Sensitivity....9
  - Ambiguities....9
  - Sold by....9
  - Filter....9

Editing the enzyme list....10
- Adding an Enzyme....10
  - Name....10
  - Enzyme....10
  - Companies....10
  - Methylation....10

Editing an Enzyme....11
- Deleting an Enzyme....11
- Adding and modifying categories....11
- Moving and copying enzymes....11

Search Enzymes....11
Running the Program....11
Summary Tab....12
Best Table....13
- Enzymes....13
- Unique Cuts....13
- Premature Cuts....13
- Cuts....13

Top Table....14
- Enzymes....14
- Unique Cuts....14
- Premature Cuts....14

Gel Visualization....15
- Sequence Selector....15

Enzyme Selector....16
- Linking Sequences....16
- Enzyme sorting....16
- Standard Visualization Mode....16
- Single Digest Mode....16
- Gel Visualization Panel....16

Bleed and Intensity Visualization Slider..17
Scale Visualization Slider 17
Save Gel Image 17
Additional Options 17
Save and Load Project...17
Update Enzyme List 17
Export Enzyme List 17
Retain Intermediate Files 17
Warn about gaps in File...18
Set Perl Command..18
Cut Locations 18
Create Neo(Iso)schizomers Report..19
Errors and warnings 20
Gel Image – Unsupported format selected...20
Load Project – Bad File Name 20
Load Project – Access Error..20
Load Project – Load Error 20
Perl Command – Cannot find Perl Command 20
Run – You must select at least 1 enzyme...20
Run – Fasta file does not have poly A tails...20
Run - After trimming, all fasta entries were eliminated 21
Save Project – Bad File Name 21
Save Project, Save Table Data – Save Error..21
Out of Memory Error..21
Other Errors 21
Overwrite existing file 21
Filter Dialog – At least one [cut type, company, recognition site] must be selected..21
Filter Dialog - Other Recognition Site length must be a positive integer 21
Error message when updating enzymes from NEB...22
Gel Visualization - You may only remove linked sequences...................................22

Introduction

C*is*SERS: Customizable *in silico* Sequence Evaluation for Restriction Sites.

C*is*SERS is a graphical user interface that assists in identifying restriction sites in sequence data.

Capabilities include:

- Finding enzyme restriction cut sites for sequences of interest.
- Finding a combination of enzymes that cut the majority of sequences.
- Trimming and orientation of poly-A tail sequences
- Visualizing the difference in how gels look when run with different sequences or enzymes.
- Raw output of all places the enzymes cut.

Usage Diagram

Fasta File to Analyze

Indicates that all sequences have poly-A tails

Determines if bases should be skipped when searching on 3' end or 5' end

Save or load the list of Enzymes and which ones are selected

Tri-state checkbox, can be selected to select or deselect all items in that category.

Enzyme checkbox select to include in enzyme search.

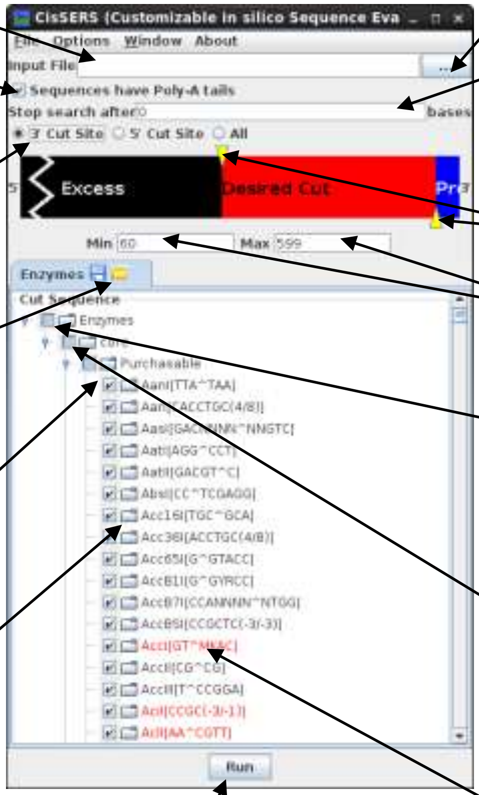

Start the analysis

Browse file system for fasta file

Number of bases that may prepend a poly-A tail
Only visible when Poly-A option is selected.

Adjust cut location on slider

Manually set slider position. Slider will auto update.

Root enzyme category. Can be renamed, but not removed.

Core category.
This category is reinitialized when a new enzyme list is downloaded.
If renamed, it will not be overwritten when a new list is downloaded

Enzyme name with recognition site and cut location. Red indicates may cut differently based on methylation. Right click to edit, delete, or add a new enzyme to this category Drag and drop to move.

Prerequisites

You will need the following to be able to run C*is*SERS:

A Desktop or laptop computer running Windows 2000 or higher, Mac OS 10.5 or higher, or a Java compatible version of Linux.

Java 5.0 or higher. Windows and Linux users can get Java from http://www.java.com. For Mac, Java is included as part of the operating system.

Perl, downloadable from http://www.perl.org. Mac and Linux users should find that perl is already installed on their system.

There is no explicit RAM, HD, or CPU requirements. More RAM will allow for larger data sets to be processed.

Project setup

Before you get started with *Cis*SERS, you will need to have the nucleotide sequences you want to analyze on your computer in a single multi fasta file.

When *Cis*SERS first opens you will be presented with a window that looks like:

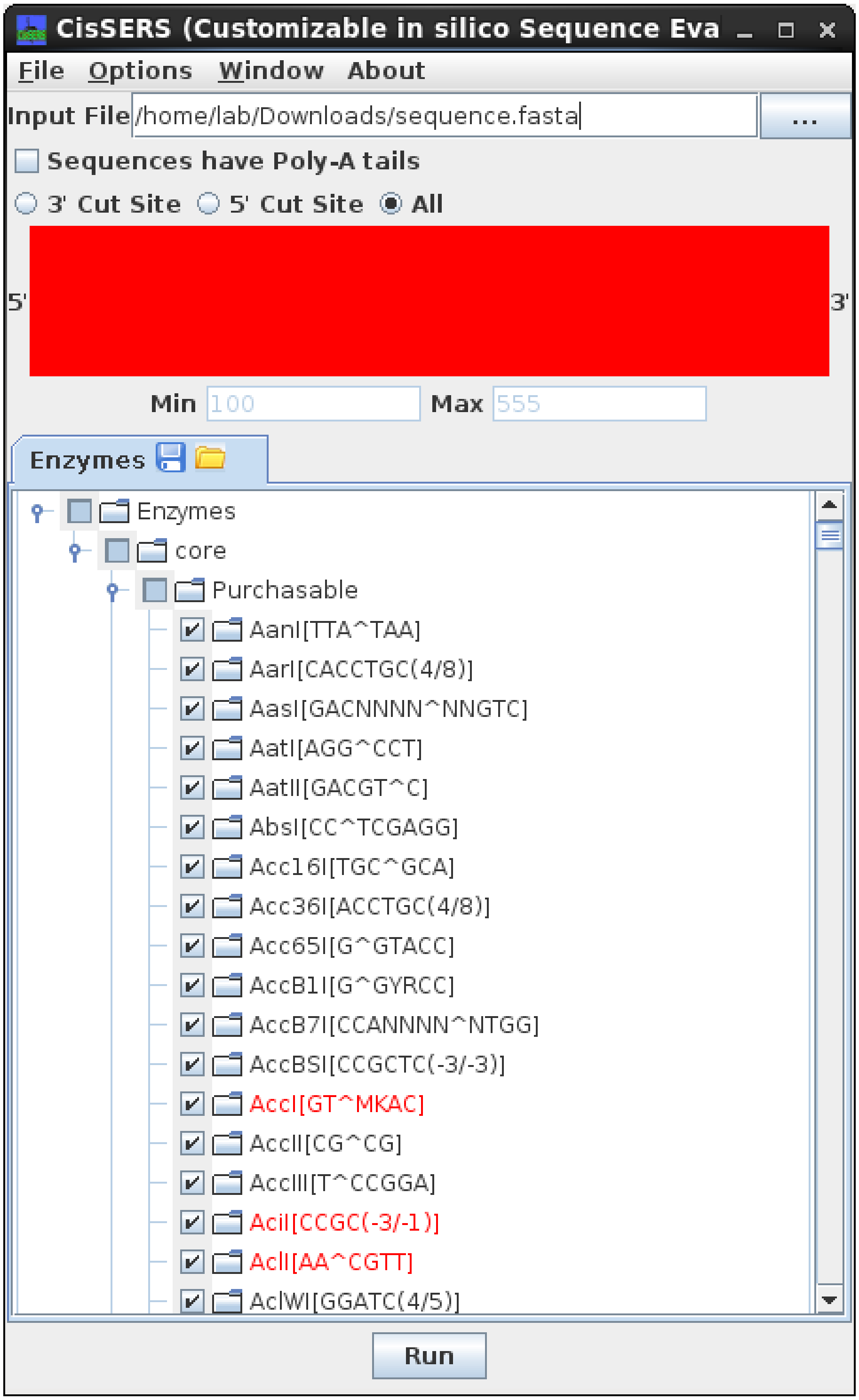

The actual screen may differ based on your operating system.

Select Input File

In the Input File text box, select the fasta file that contains all the sequences you want to process. You can click the “...” button to select a file from an open dialog box. C*is*SERS needs write permission to the folder where the fasta file is in order to generate preprocessed fasta files.

Decide if Poly-A tails are present

If your sequences have Poly-A tails, you should check this box. When this box is checked, another text box will appear below allowing you to decide how many junk base pairs you expect before poly-A tails. In most cases, the default value of 0 will work. Selecting this option will remove gel visualization from being shown after processing.
When poly-A tails option is selected, only sequences that have a poly-A tail will be considered, and will be appropriately oriented if the sequence is reversed.

When this box is not checked, your data should not have poly-A tails, and the sequences should be oriented.

Decide Cut site

The area of the sequences that are analyzed with the choice of 5’ end, 3’ end or the entire sequence.

5' Cut Site: Identify restriction sites within a user defined number of bases of the 5' end.
3' Cut Site: Identify restriction sites within a user defined number of bases of the 3' end.
All: Identify all restriction sites across the entire sequence.

If you choose the 3' Cut Site or the 5' Cut site, the double slider bar below:
You can adjust the Desired Cut area by dragging the yellow knobs, or by dragging the red Desired Cut box itself. As you adjust the location, the Min and Max text boxes will adjust to indicate the numeric range. The text boxes can also be edited to manually adjust the targeted Desired Cut area. Manual adjustment is necessary for a Desired Cut area past 1000 bp.

Select Desired Enzymes

Enzymes appear with their name on the left and their recognition site and cut location within square brackets to the right. Enzymes in red are sensitive to methylation. A ^ in the recognition site indicates a cut location within or adjacent to the recognition site, while two numbered in parenthesis indicate a cut location that is fully or partly outside the recognition site.

Manual Enzyme selection

When you first use C*is*SERS, a default list of enzymes is preloaded into the Enzymes tab. If you want to select all enzymes, or a category of enzymes (Purchasable or non-Purchasable) you may select the folder for that. Similarly, if you want to select only a few select enzymes you may manually select them from the list.

Selecting Enzyme by Criteria

If you want to select enzymes that match chosen criteria, you may use the Filter Enzymes menu option. This will show a dialog where you can choose which enzymes you want to select based on indicated criteria:

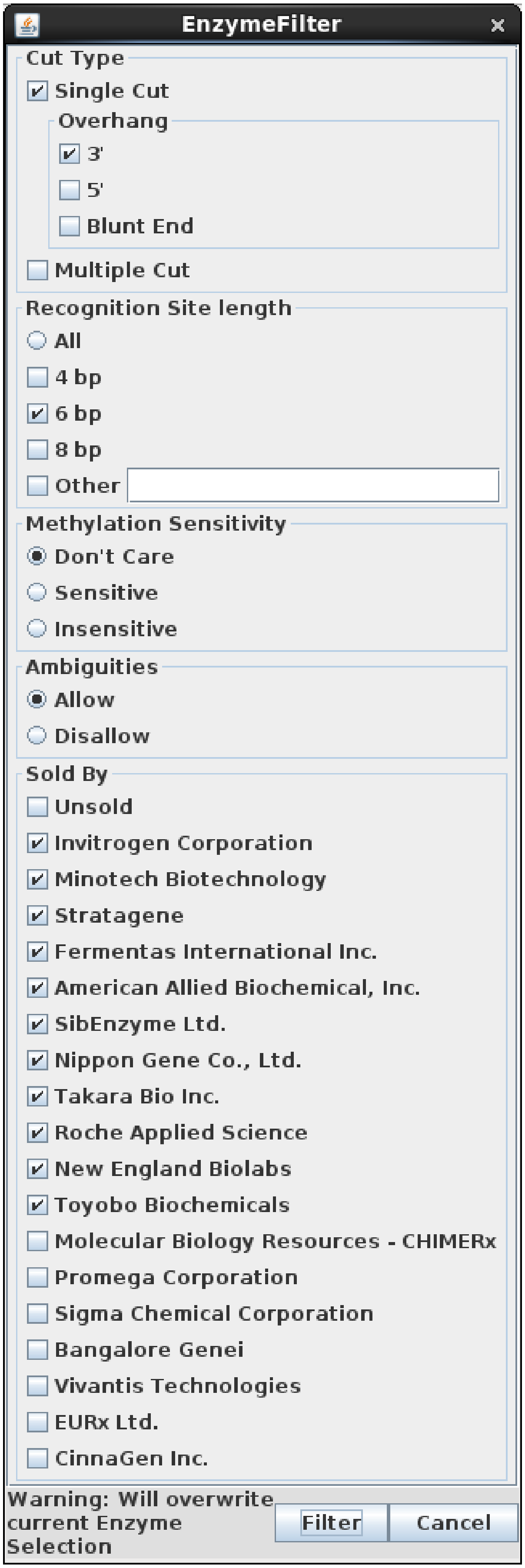

Cut Type

Single Type: Allows enzymes with one cut location.
Overhang 3': Allow enzymes that cut with a 3' overhang.
Overhang 5': Allow enzymes that cut with a 5' overhang.
Blunt End: Allow enzymes that cut bluntly.
Multiple Cut: Allow enzymes with two or more cut locations.
Note that enzymes that aren't annotated with a cut location are not filtered out in this section.

Recognition Site length

This filters based on the size of the recognition site. The other size allows for positive integer value recognition site lengths. The recognition site length does not consider Ns on the left or right side as part of the recognition site (while Ns in the middle do count). This means that the cut location can occur outside the recognition site.

Methylation Sensitivity

A methylation sensitive enzyme is one that may act differently if methylation is present or absent. Please consult external documentation on methylation sensitive enzymes for further details about how the enzyme acts with respect to methylation.

Ambiguities

An enzyme with ambiguities can cut on multiple recognition sites. Enzymes without ambiguities will only cut on sites annotated with all AGCTs within its recognition site. An enzyme annotated with Ns on the left or right of its recognition site will not exclude the enzyme from being considered unambiguous.

Sold by

Only enzymes that are sold by at least one of the selected companies will not be filtered out. If unsold is selected, then enzymes that are not sold by the companies below are also candidates for filtering. Note that selecting unsold while not selecting all other companies will probably not be very helpful, as if an enzyme is only sold by one of the unselected companies it will not be selected.

Filter

Once you press the filter button, all previous selected enzymes will be cleared, and a new set of enzymes will be selected based on the selected filter options. Pressing cancel will dismiss this dialog without making any changes.

Editing the enzyme list

If any changes need to be made to the enzyme list they can be done by right clicking an enzyme or its category, or by dragging an enzyme to a new location. Changes that are made to the enzyme list will not change the default startup list, and can be saved as part of the project, or separately by itself. Any changes you make outside the core category will be overwritten once you choose to “Update Enzyme List” in the File menu.

Adding an Enzyme

By right clicking a category or an enzyme you may “Add Enzyme”. Adding an enzyme while right clicking on another enzyme will add it to that enzyme's category.

Name

Enter the name of the enzyme, the name cannot include the # symbol.

Enzyme

Enter the recognition site and cut location of the enzyme here.

Companies

Enter a string of characters where each character represents the companies the enzyme is sold by:

B Invitrogen Corporation
C Minotech Biotechnology
E Stratagene
F Thermo Scientific Fermentas
I SibEnzyme Ltd.
J Nippon Gene Co., Ltd.
K Takara Bio Inc.
M Roche Applied Science
N New England Biolabs
Toyobo Biochemicals
Q Molecular Biology Resources - CHIMERx
R Promega Corporation
S Sigma Chemical Corporation
U Bangalore Genei
V Vivantis Technologies
X EURx Ltd.
Y CinnaGen Inc.

For example, EKX means that Invitrogen, Takara Bio, and EURx sell this enzyme. As you sync with NEBs database it is possible that additional companies will be added. In this case refer to http://rebase.neb.com/rebase/link_nar for a list of companies and their associated letter.

Methylation

Check this box to indicate that the enzyme is sensitive to methylation.

Editing an Enzyme
Editing an enzyme is similar to adding an enzyme. The boxes to edit the enzyme's info are filled in with the current information.

Deleting an Enzyme
Deleting an enzyme is done by selecting an enzyme, selecting delete. A confirmation dialog box is shown to avoid a misclick, as there is no undo function.

Adding and modifying categories
A category can be added by right clicking an enzyme or category and selecting “Add Category”. Whether an enzyme is selected or the base category, the new category will be added as a subcategory directly to the base category. After this, type in the category's name in the dialog box. Editing a category's name is done by selecting a category and selecting Edit.

Moving and copying enzymes
An enzyme can be moved to another location in a different category, or reordered within the same category by clicking and dragging the enzyme to the destination you wish to place it. Copying an enzyme is done by holding control while moving the enzyme. This is primarily useful for copying existing enzymes to a new custom category of “favorite” enzymes.

Search Enzymes
The search Enzymes feature, available in the File menu, allows for the quick navigation of an enzyme based on its name or recognition site.
Searching can be done either by typing in the name of the enzyme. Typing in the first part of an enzyme's name will show all enzymes that start with the text that is put in. Typing in the recognition site will match all enzymes that have that exact recognition site. As in the above screen shot, there are nine sequences that have a recognition site of AAGCTT, but only one of those is actively sold by companies.

Running the Program
When you are ready to begin processing, click the Run button.
A progress tab will appear indicating the progress:
If any errors occur during processing an error message dialog will appear indicating the error.

After processing is complete, the processing tab will close, and the best tab, summary tab, and if poly-A tails option is not selected, a Gel Visualization tab will open.

Summary Tab

After processing the summary tab gives detailed information about the process.
The disk icon on the Summary tab can be used to save the summary data to a csv file, which can be imported into a spreadsheet program like Excel.
Enzyme column: This is the name of an enzyme that cut. If a * is after the name, then multiple enzymes that shared the same recognition site were consolidated. See the Neo(Iso)schizomer report in the Window menu for a detailed view on what enzymes where consolidated.
Cut Seq: This is the recognition site of the enzyme
Seq Cut: This is the total number of sequences that have the recognition site in them at least once.
Total Cuts: This is the total number of cuts across all sequences that don't entirely fall within the pre-cut area.
Percent of Total Sequences Cut: This is the percentage of sequences that were cut at least once that don't entirely fall within the pre-cut area.

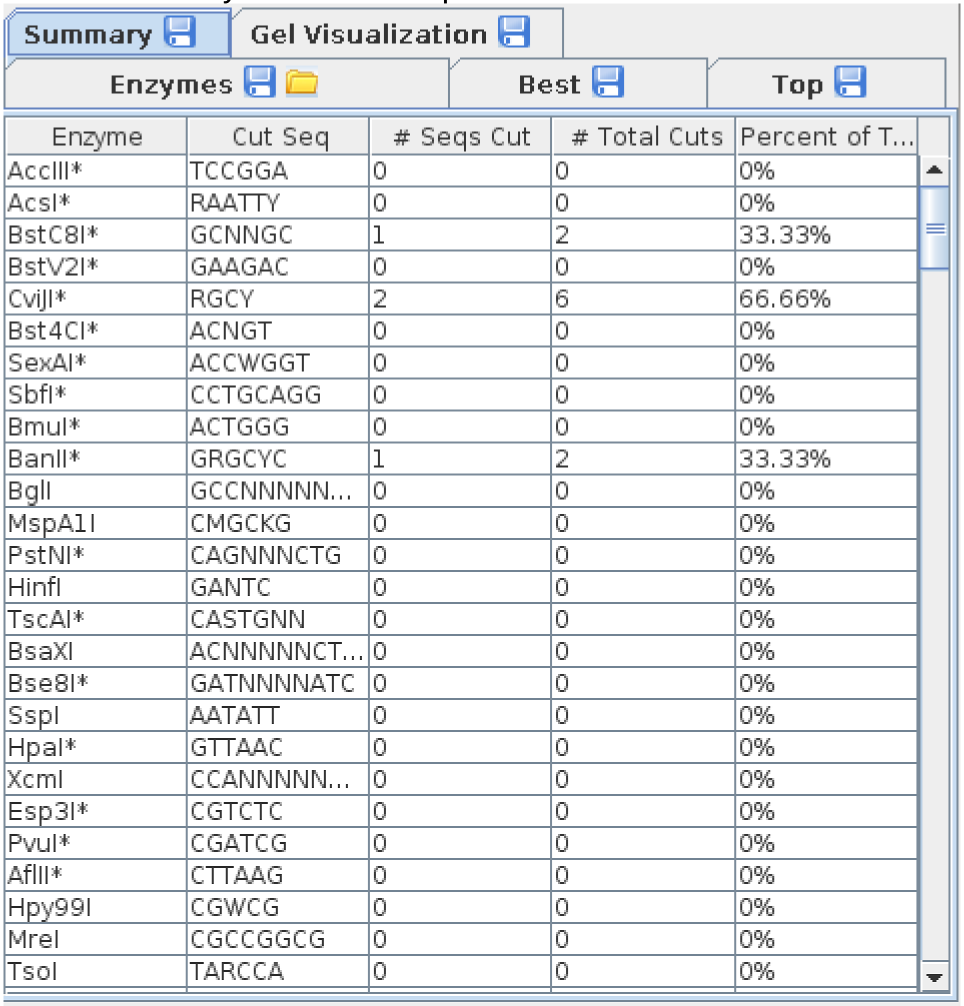

Enzyme	Cut Seq	# Seqs Cut	# Total Cuts	Percent of T...
AccIII*	TCCGGA	0	0	0%
AcsI*	RAATTY	0	0	0%
BstC8I*	GCNNGC	1	2	33.33%
BstV2I*	GAAGAC	0	0	0%
CviJI*	RGCY	2	6	66.66%
Bst4CI*	ACNGT	0	0	0%
SexAI*	ACCWGGT	0	0	0%
SbfI*	CCTGCAGG	0	0	0%
BmuI*	ACTGGG	0	0	0%
BanII*	GRGCYC	1	2	33.33%
BglI	GCCNNNNN...	0	0	0%
MspA1I	CMGCKG	0	0	0%
PstNI*	CAGNNNCTG	0	0	0%
HinfI	GANTC	0	0	0%
TscAI*	CASTGNN	0	0	0%
BsaXI	ACNNNNNCT...	0	0	0%
Bse8I*	GATNNNNATC	0	0	0%
SspI	AATATT	0	0	0%
HpaI*	GTTAAC	0	0	0%
XcmI	CCANNNNN...	0	0	0%
Esp3I*	CGTCTC	0	0	0%
PvuI*	CGATCG	0	0	0%
AflII*	CTTAAG	0	0	0%
Hpy99I	CGWCG	0	0	0%
MreI	CGCCGGCG	0	0	0%
TsoI	TARCCA	0	0	0%

Best Table

Enzymes Best Top Summary

Enzyme	Unique Cuts	Premature Cuts	Cuts
Bst6I*	30.82%	7.05%	8784
BstV2I*	13.85%	2.63%	6280
BsmBI*	10.49%	3.32%	5369
BseRI	7.69%	1.89%	7612
SpeI*	5.63%	2.42%	3324
BpuEI	4.21%	1.21%	4805
Total	72.72%	18.55%	0

The best table shows a collection of up to 6 enzymes (adjustable in the options menu) that cut the most number of sequences at least once.

Enzymes

This column is the name of the enzyme. Enzymes with a * at the end represent multiple enzymes which all have the same recognition site.

Unique Cuts

This column is the percentage of sequences that the enzyme cuts at least once and was not cut by any of the above enzymes. Enzymes higher on the list cut more total sequences.

Premature Cuts

This column is the percentage of sequences that the enzyme cuts in the precut area and was not already cut in the precut area by an enzyme higher in the list.

Cuts

This is the total number of cuts an enzyme made though out all sequences. This does not exclude any sequences that were cut by enzymes higher in the list.

Top Table

Enzymes Best Top Summary

Enzyme	Unique Cuts	Premature Cuts
AcsI*	100%	66.66%
StyI*	100%	33.33%
TsoI	100%	33.33%
AcuI*	100%	0%
BseDI*	100%	33.33%
MmeI	100%	66.66%
Bst6I*	100%	0%
SmlI*	100%	33.33%
PlaDI	100%	0%
Eco57MI	100%	0%
Hpy188III*	100%	33.33%
CchII	100%	0%
BsiWI*	100%	0%
BstNSI*	100%	0%
Tth111II	100%	0%

The top table shows all enzymes that cut 95% (adjustable in the options menu) of the sequences (in either the desired cut area or the pre-cut area).

Enzymes

This column is the name of the enzyme. Enzymes with a * at the end represent multiple enzymes which all have the same recognition site.

Unique Cuts

This column is the percentage of sequences the enzyme cuts at least once in the desired cut region.

Premature Cuts

This column is the percentage of sequences that the enzyme cut in the pre-cut.

Gel Visualization
The Gel Visualization is a visual guide to indicate how a gel will look and the relative locations of the bands.

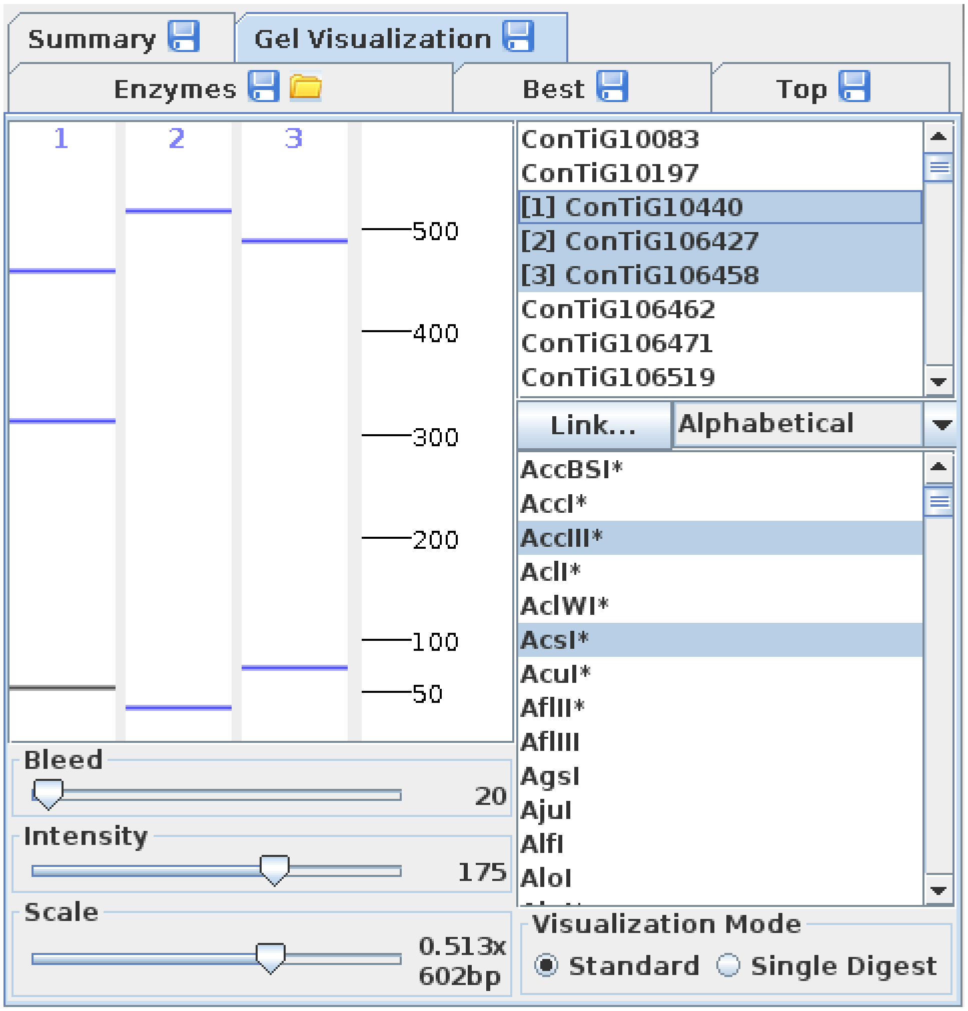

Sequence Selector
The upper right list contains all sequences that were processed. In order to display a gel image, at least 1 sequence must be selected (multiple sequences can be selected by holding down the control key and clicking in standard visualization mode) and at least 1 enzyme must be selected. Each sequence selected gets its own gel lane on the left. The number in brackets by a selected sequence indicates its id which will appear in on the gel visualization when there isn't enough room to display the full name of the contig in standard mode.

Enzyme Selector

The lower right list contains a list of all processed enzymes. Enzymes with a star following its name have Neo(Iso)schizomers which are not shown in the list. Check the Neo(Iso)schizomers report from the Window menu if you don't see the enzyme you are interested in. The number in brackets to the right of the enzyme name is the identifier that is displayed on top of each gel lane if there is insufficient room for the enzyme name in single digest mode. Each enzyme that is selected will be part of the gel visualization display (multiple enzymes can be selected by holding down the control key and clicking). A gel is displayed once at least 1 enzyme and at least 1 sequence are selected.

Linking Sequences

By clicking the Link button, you may select any of sequences to be linked together into a single gel lane. After giving the new sequence combination a name (which must be unique), it will be added to the sequence list.

Enzyme sorting

By selecting from the drop down box right of the link button, enzymes can be sorted. The default sort is Alphabetical, which sorts the enzymes according to name. Difference score will give each enzyme a score based on differentiating the currently selected sequences from each other. If any change to the currently selected enzyme or sequence is made no change to the order is made, and the sorting method is changed to Custom (there is no current way to manually sort the enzymes).

Standard Visualization Mode

Standard visualization Mode is the default, and can be selected under the Visualization mode box. In standard mode, each sequence constitutes its own lane. The bands that appear in the visualization are the expected cuts if a full digest is done on the sequence using all the selected enzymes.

Single Digest Mode

In single digest mode, only one sequence can be selected, and each gel lane represents using one of the selected enzymes to digest the sequence. If only one sequence and one enzyme is selected, this will produce the same results as standard mode, except the header for the lane will be the name of the enzyme instead of the name of the sequence.

Gel Visualization Panel

The Gel Visualization panel shows the predicted gel based on the parameters inputted. The bands that are shown are spaced linearly unlike a real gel. The ladder on the right indicates where the band is place. Blue bands indicate the start and end bands of the sequence, and may be slightly inaccurate based on where the enzyme cuts, or may be absent if the sequence is circular. The header for the lane will be the name of the enzyme or sequence for that lane if room permits, or a number which is prepended in brackets in the sequence or enzyme list if room does not permit.

Bleed and Intensity Visualization Slider

This bleed slider can be used to increase how much each band bleeds, while the intensity slider indicates how quickly each band fades. Higher values may help to indicate the difficulty in distinguishing lanes.

Scale Visualization Slider

Use this slider to adjust how much a gel lane is visible. This slider is automatically adjusted each time a new sequence/enzyme selection is made such that the highest band is close to the top of the visualization. If any of the bands are clipped off by zooming in, a scroll bar will appear to allow seeing further up the gel.

Save Gel Image

By clicking the disk icon in the Gel Visualization tab you may save the current gel image as seen on the screen (with an added legend) to an image file. Available formats are png, gif, jpeg, and bmp.

Additional Options

Save and Load Project

You may save or load the current project settings and results (if any) under the File menu. Only the parameters used to run the program and the mediate results are saved. Currently selected items in any table or the gel visualization, any created linked sequences, and the setting in gel visualization are not saved. Loading a project will overwrite the current settings. If you need multiple projects open at once, you can open multiple instances of the program (on a Mac you will need to use the terminal to open a second instance of the program).

Update Enzyme List

You may update the list of enzymes from NEB from the File -> Update Enzyme List. This will download data from http://rebase.neb.com/rebase/link_nar and populate the core category in the enzyme list. When you do this it will overwrite any changes you have made to the core category. It is possible that this operation may fail if the above link is not accessible, or NEB has modified the format of this file. This will only update the list for the current running instance of the program. It is recommend that you save the enzyme list using the disk icon on the Enzymes tab and choose to have it automatically open on program startup. Any previously saved projects will still use the older enzyme list.

Export Enzyme List

This is an advanced feature that saves the currently checked enzymes to a single flat file used for processing.

Retain Intermediate Files

When this is checked any intermediate fasta files generated by preprocessing are kept. When this is not checked, these files are deleted on exit.

Warn about gaps in File

If your input file has gaps in it and this option is checked, then you will see a warning that gaps are removed before processing. You can enable or disable this option in the Options menu.

Set Perl Command

Under the Options menu you can change how C*is*SERS uses perl. If you have installed perl in a non-standard location you can use this to set the path to where perl is located.

Cut Locations

Show Cut Locations in the Window menu will display all the locations in all the sequences where a cut occurs in a table.

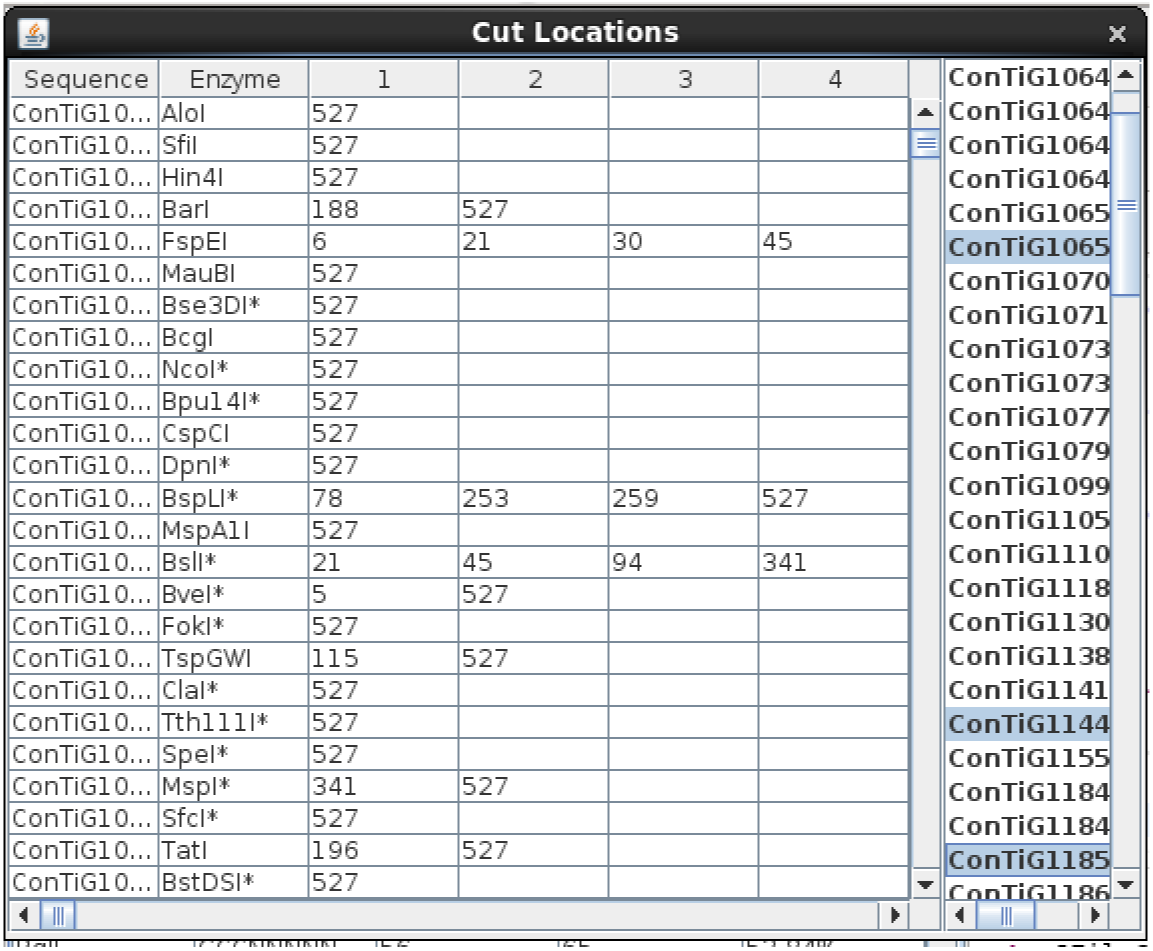

Sequence	Enzyme	1	2	3	4
ConTiG10...	AloI	527			
ConTiG10...	SfiI	527			
ConTiG10...	Hin4I	527			
ConTiG10...	BarI	188	527		
ConTiG10...	FspEI	6	21	30	45
ConTiG10...	MauBI	527			
ConTiG10...	Bse3DI*	527			
ConTiG10...	BcgI	527			
ConTiG10...	NcoI*	527			
ConTiG10...	Bpu14I*	527			
ConTiG10...	CspCI	527			
ConTiG10...	DpnI*	527			
ConTiG10...	BspLI*	78	253	259	527
ConTiG10...	MspA1I	527			
ConTiG10...	BslI*	21	45	94	341
ConTiG10...	BveI*	5	527		
ConTiG10...	FokI*	527			
ConTiG10...	TspGWI	115	527		
ConTiG10...	ClaI*	527			
ConTiG10...	Tth111I*	527			
ConTiG10...	SpeI*	527			
ConTiG10...	MspI*	341	527		
ConTiG10...	SfcI*	527			
ConTiG10...	TatI	196	527		
ConTiG10...	BstDSI*	527			

The left list allows selecting which sequences you want to view in the table. The sequence and enzyme columns are a pair of all possible sequence and enzyme combinations. Each column following that is a location where that enzyme is found in the sequence.

Create Neo(Iso)schizomer Report

This option, under the Windows menu, is used to see what enzymes share the same recognition site. Since C*is*SERS combines all enzymes that share the same recognition site, this is useful to find the alias entry that an enzyme is listed under.

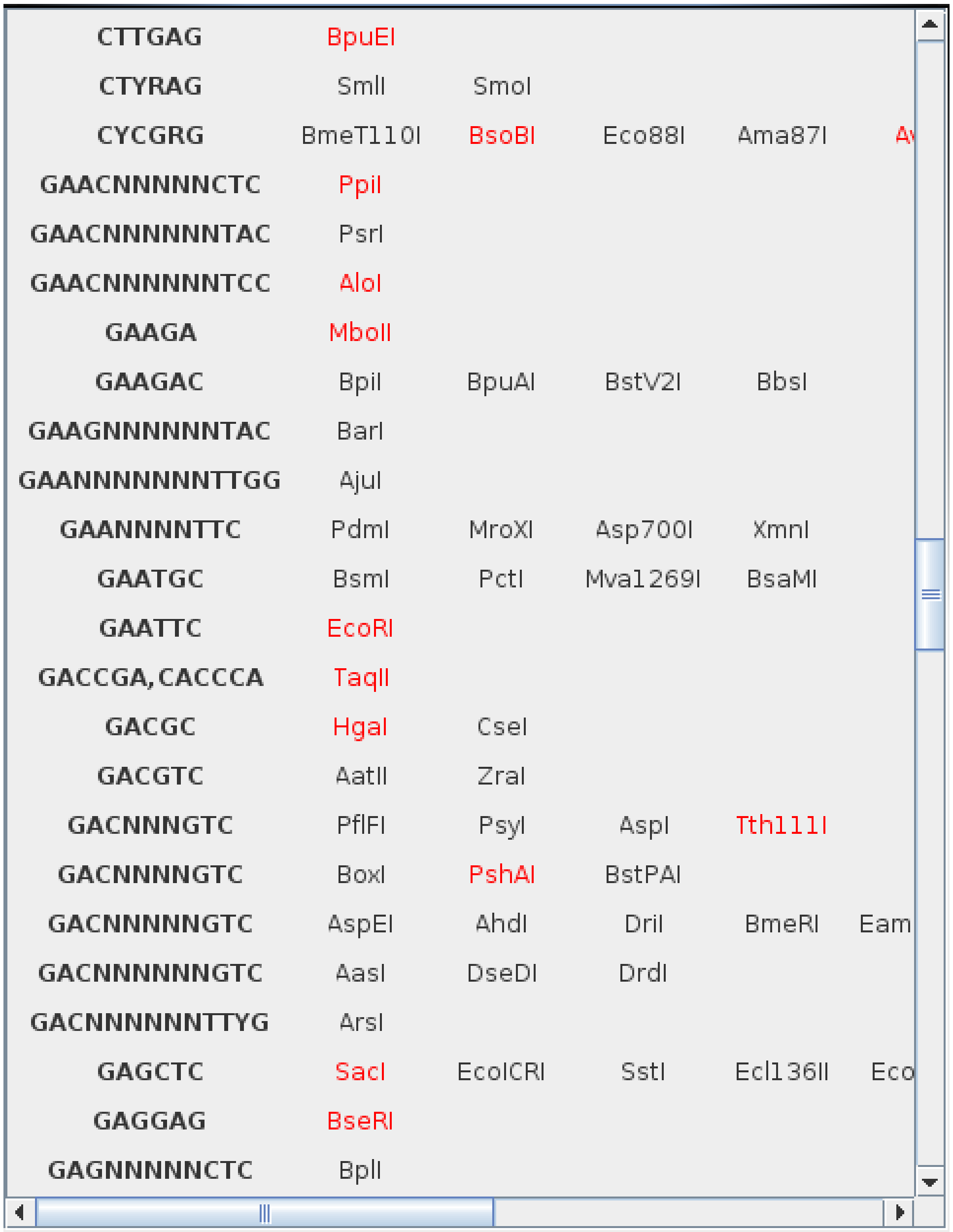

**CTTGAG**	BpuEI				
**CTYRAG**	SmlI	SmoI			
**CYCGRG**	BmeT110I	BsoBI	Eco88I	Ama87I	A
**GAACNNNNNCTC**	PpiI				
**GAACNNNNNNTAC**	PsrI				
**GAACNNNNNNTCC**	AloI				
**GAAGA**	MboII				
**GAAGAC**	BpiI	BpuAI	BstV2I	BbsI	
**GAAGNNNNNNTAC**	BarI				
**GAANNNNNNNTTGG**	AjuI				
**GAANNNNTTC**	PdmI	MroXI	Asp700I	XmnI	
**GAATGC**	BsmI	PctI	Mva1269I	BsaMI	
**GAATTC**	EcoRI				
**GACCGA,CACCCA**	TaqII				
**GACGC**	HgaI	CseI			
**GACGTC**	AatII	ZraI			
**GACNNGTC**	PflFI	PsyI	AspI	Tth111I	
**GACNNNGTC**	BoxI	PshAI	BstPAI		
**GACNNNNNGTC**	AspEI	AhdI	DriI	BmeRI	Eam
**GACNNNNNNGTC**	AasI	DseDI	DrdI		
**GACNNNNNNTTYG**	ArsI				
**GAGCTC**	SacI	EcoICRI	SstI	Ecl136II	Eco
**GAGGAG**	BseRI				
**GAGNNNNNCTC**	BplI				

Errors and warnings

The following is a list of many common errors and warning you may receive. This is not a comprehensive list, any errors or warnings not mentioned are either bugs, or unforeseen issues often related to being able to read or write data.

Gel Image – Unsupported format selected

PNG, JPEG, GIF, and BMP are the supported image formats for exporting to a Gel Image. Please make sure your saved file name ends in one of: .jpg, .png, .jpeg, .gif

Load Project – Bad File Name

The project you tried to load was not found. It was probably deleted between the time you selected it and tried to load it.

Load Project – Access Error

The project file could not be fully read. Make sure you have read permission to this file, and that you didn't unplug an external drive that contained the file while it was loading.

Load Project – Load Error

The project file was not in the correct format. Make sure the file you select was saved using C*is*SERS, and that you are using the same version of C*is*SERS to load the project file that you saved it with. C*is*SERS is not guaranteed to be able to load project saved from different versions.

Perl Command – Cannot find Perl Command

Perl was not found in the location specified under Options -> Set Perl Command. Make sure that Perl is installed, and that the perl command option points to the location that perl is installed too.

Run – You must select at least 1 enzyme

At least one enzyme must be selected from the enzyme tab to do any processing.

Run – Fasta file does not have poly A tails

No poly-A tails were found in the sequences and the “Sequences have Poly-A tails” option was selected. If your sequences don't have poly-A tails, or they were already removed, deselect this option. If there are non-poly-A base pairs before the poly-A tail, you can set the threshold of how far to look into the sequence by specifying the “Stop search after” option below the Poly-A tail checkbox (when selected). Otherwise, please manually remove poly-A tails and do any needed reverse complementing of sequences with poly-T heads, as C*is*SERS cannot recognize these poly-A tails.

Run - After trimming, all fasta entries were eliminated

At least one poly-A tail was found, but all sequences either had a poly-A tail with no sequence, no poly-A tail, or both a poly-A tail and a poly-T head. If you have Stop search after option as non-0 consider reducing this value to avoid fully trimming your sequences.

Save Project – Bad File Name

You have either selected a file that is special to the operating system, such as null on Windows, or a folder with the same name as the chosen project file name was created while the save dialog was displayed.

Save Project, Save Table Data – Save Error

There was a problem while saving. Ensure you can write to the location you specified (e.g. don't try to save to a CD), and that you didn't remove any external drive that you were saving too. It could also mean that your hard drive is going bad.

Out of Memory Error

C*is*SERS has run out of memory. The program may or may not continue to work from this point on (restarting is recommended). Try shutting down all other programs, running on a computer with more RAM, or reducing your data set.

Other Errors

If you see a dialog with the title: "An unknown error has occurred," it probably indicates a bug in the program. Please contact us so that we can fix it. Sorry for the inconvenience.

Overwrite existing file

When saving a file, you will see this confirmation dialog if you the file you are writing to already exists. If you proceed the previous version will be lost.

Filter Dialog – At least one [cut type, company, recognition site] must be selected

You have attempted to apply a filter on the enzymes with all choices for a category deselected. Select one or more options in this category.

Filter Dialog - Other Recognition Site length must be a positive integer

Recognition site length must be a positive integer. Please input this value using numbers 0 through 9 (e.g. 7.00 is not an acceptable value, whereas 7 is).

Error message when updating enzymes from NEB

If you get an error message when updating from NEB, this could be due to numerous reasons. First, ensure that your internet is working and you can contact http://www.neb.com. Otherwise, NEBs enzyme may be down, or they may have changed their enzyme list format in a way that C*is*SERS can no longer read.

Gel Visualization - You may only remove linked sequences

When you delete a sequence using the delete key, only pseudo linked sequences created with the link button can be removed.

java.lang.NullPointerException

During the review process, one reviewer reported this exception error with JRE 8 and 9 on machines running Ubuntu 14.04.2, x86_64 platform and specific builds of Java. However, the same reviewer confirmed that there are no issues with JRE 7.
CisSERS has been evaluated on Redhat Enterprise Linux platform, CentOS release 6.5, Windows 7 and Windows 10 without the NPE error.
