# Supplementary material for: CisSERS: Customizable In Silico Sequence Evaluation for Restriction Sites
Source: PLoS One. 2016 Apr 12;11(4):e0152404. doi: 10.1371/journal.pone.0152404 (PMC4829253; doi:10.1371/journal.pone.0152404)
Supplement: S3 File — (PDF) [file pone.0152404.s003.pdf]

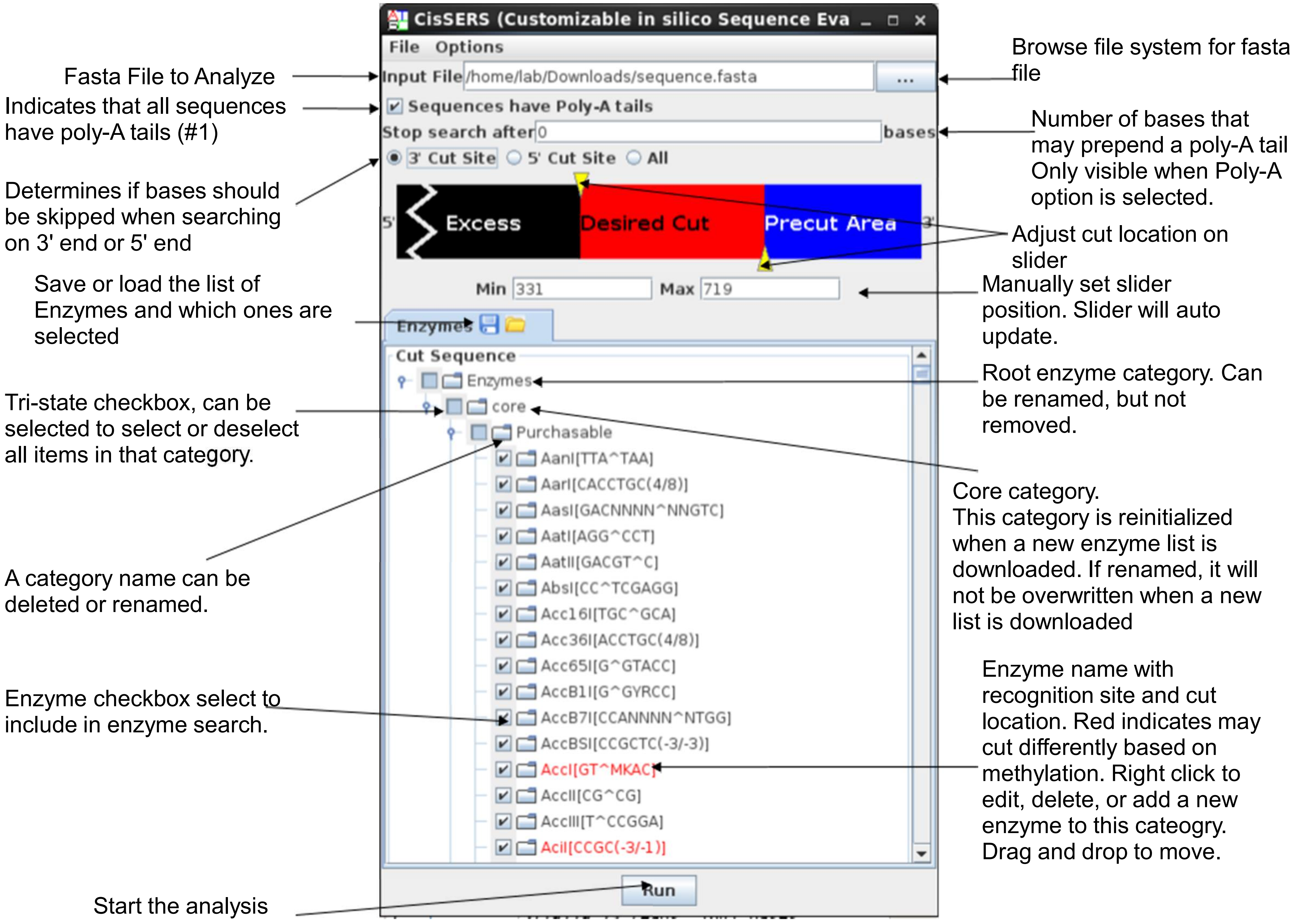
CisSERS (Customizable in silico Sequence Eva
File Options
Input File /home/lab/Downloads/sequence.fasta
...
Sequences have Poly-A tails
Stop search after 0 bases
3' Cut Site
5' Cut Site
All
5'
Excess
Desired Cut
Precut Area
3'
Min 331
Max 719
Enzymes
Cut Sequence
Enzymes
core
Purchasable
AanI[TTA^TAA]
AarI[CACCTGC(4/8)]
AasI[GACNNNN^NNGTC]
AatI[AGG^CCT]
AatII[GACGT^C]
AbsI[CC^TCGAGG]
Acc16I[TGC^GCA]
Acc36I[ACCTGC(4/8)]
Acc65I[G^GTACC]
AccB1I[G^GYRCC]
AccB7I[CCANNNN^NTGG]
AccBSI[CCGCTC(-3/-3)]
AccI[GT^MKAC]
AccII[CG^CG]
AccIII[T^CCGGA]
AciI[CCGC(-3/-1)]
Run
Fasta File to Analyze
Indicates that all sequences have poly-A tails (#1)
Determines if bases should be skipped when searching on 3' end or 5' end
Save or load the list of Enzymes and which ones are selected
Tri-state checkbox, can be selected to select or deselect all items in that category.
A category name can be deleted or renamed.
Enzyme checkbox select to include in enzyme search.
Start the analysis
Browse file system for fasta file
Number of bases that may prepend a poly-A tail Only visible when Poly-A option is selected.
Adjust cut location on slider
Manually set slider position. Slider will auto update.
Root enzyme category. Can be renamed, but not removed.
Core category.
This category is reinitialized when a new enzyme list is downloaded. If renamed, it will not be overwritten when a new list is downloaded
Enzyme name with recognition site and cut location. Red indicates may cut differently based on methylation. Right click to edit, delete, or add a new enzyme to this cateogry. Drag and drop to move.

File Menu

- Save Project – Saves all settings and the enzyme list using a save dialog box.
- Load Project – Loads a project using a load dialog box.
- Create Neo(Iso)schizomer report – Displays a list of all checked enzymes #2.
- Update Enzyme List – Connects to NEB to download a fresh copy of the enzymes. This will overwrite the core category.

File Menu Cont

- Export Enzyme List – Saves the enzymes to a file, which can be used to directly run the analysis tool from the terminal.
- Filter Enzymes – Shows a dialog that can be used to automatically select enzymes based on a criteria. #3
- Search Enzymes – Shows a window that can be used to show enzymes that match a provided name or recognition site. #4

Options Menu

- Retain intermediate fasta files – When checked, any fasta file generated from prepossessing will not be deleted on exit. These files are in the same location as the input fasta file with a similar name.
- Warn about gaps in file – When checked, a warning will be presented if the input file has gaps. Gaps are removed before processing the file.
- Set Perl Command – Sets the location of perl. When CisSERS starts up a check is done to determine if the specified command is available.

Note 1

When Poly-A tails option is selected, any sequence that has either (but not both) a poly-A tail or a poly-T head will have the poly-A tail removed. Sequences with poly-T heads will be reverse complemented beforehand. Any sequence that does not have a poly-A tail, has both a poly-A tail and a poly-T head, or is completely trimmed will not be processed. These sequences are placed in temporary files during processing that are removed when the program closes if “Retain intermediate fasta files is not selected.”

Note 2

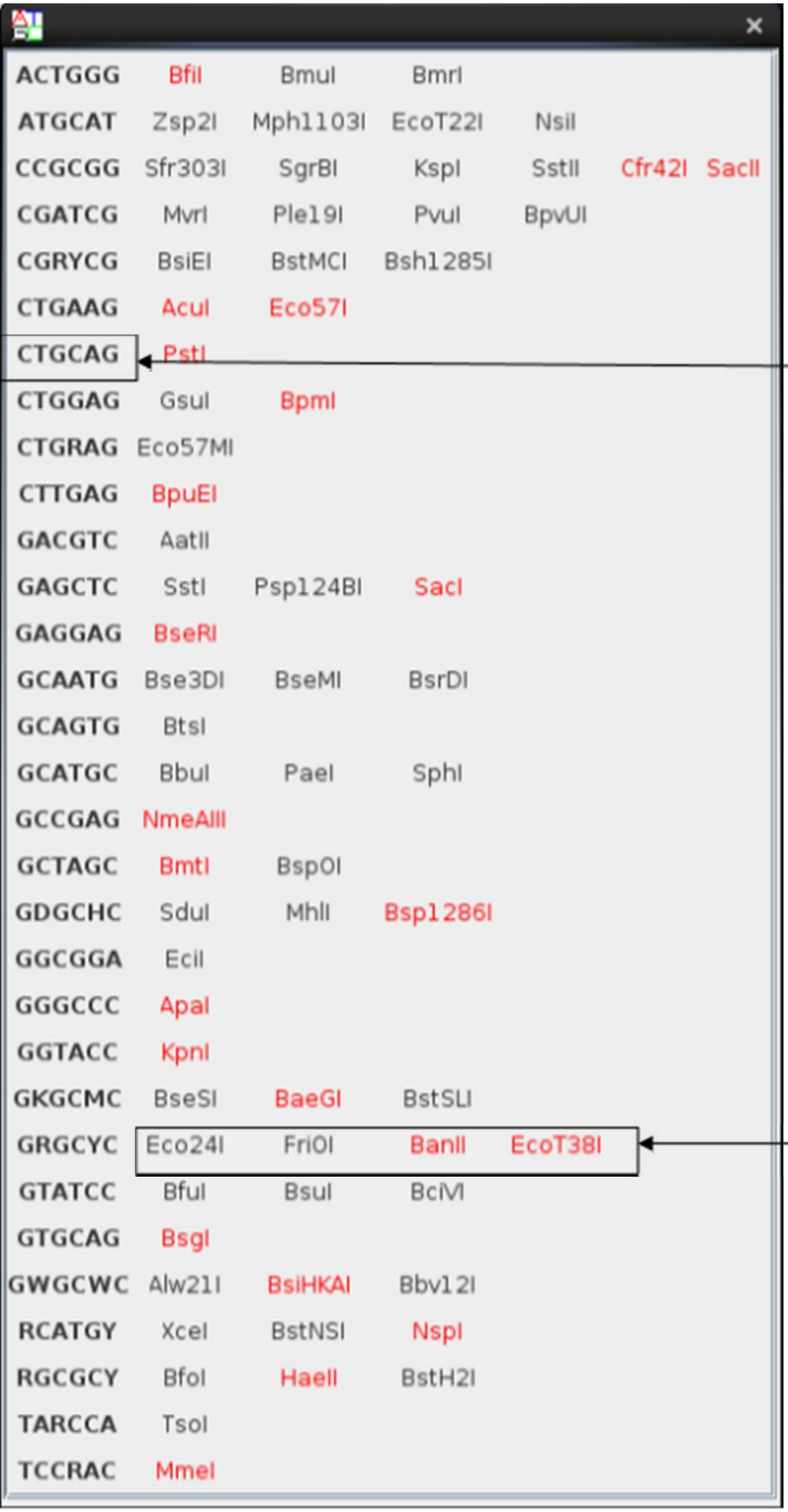

ACTGGG	BfiI	BmuI	BmrI			
ATGCAT	Zsp2I	Mph1103I	EcoT22I	NsiI		
CCGCGG	Sfr303I	SgrBI	KspI	SstII	Cfr42I	SacII
CGATCG	MvrI	Ple19I	PvuI	BpvUI		
CGRYCG	BsiEI	BstMCI	Bsh1285I			
CTGAAG	AcuI	Eco57I				
CTGCAG	PstI					
CTGGAG	GsuI	BpmI				
CTGRAG	Eco57MI					
CTTGAG	BpuEI					
GACGTC	AatII					
GAGCTC	SstI	Psp124BI	SacI			
GAGGAG	BseRI					
GCAATG	Bse3DI	BseMI	BsrDI			
GCAGTG	BtsI					
GCATGC	BbuI	PaeI	SphI			
GCCGAG	NmeAIII					
GCTAGC	BmtI	BspOI				
GDGCHC	SduI	MhlI	Bsp1286I			
GGCGGA	EciI					
GGGCCC	ApaI					
GGTACC	KpnI					
GKGCMC	BseSI	BaeGI	BstSLI			
GRGCYC	Eco24I	FriOI	BanII	EcoT38I		
GTATCC	BfuI	BsuI	BciVI			
GTGCAG	BsgI					
GWGCWC	Alw21I	BsiHKAI	Bbv12I			
RCATGY	XceI	BstNSI	NspI			
RGCGCY	BfoI	HaeII	BstH2I			
TARCCA	TsoI					
TCCRAC	MmeI					

Note 3

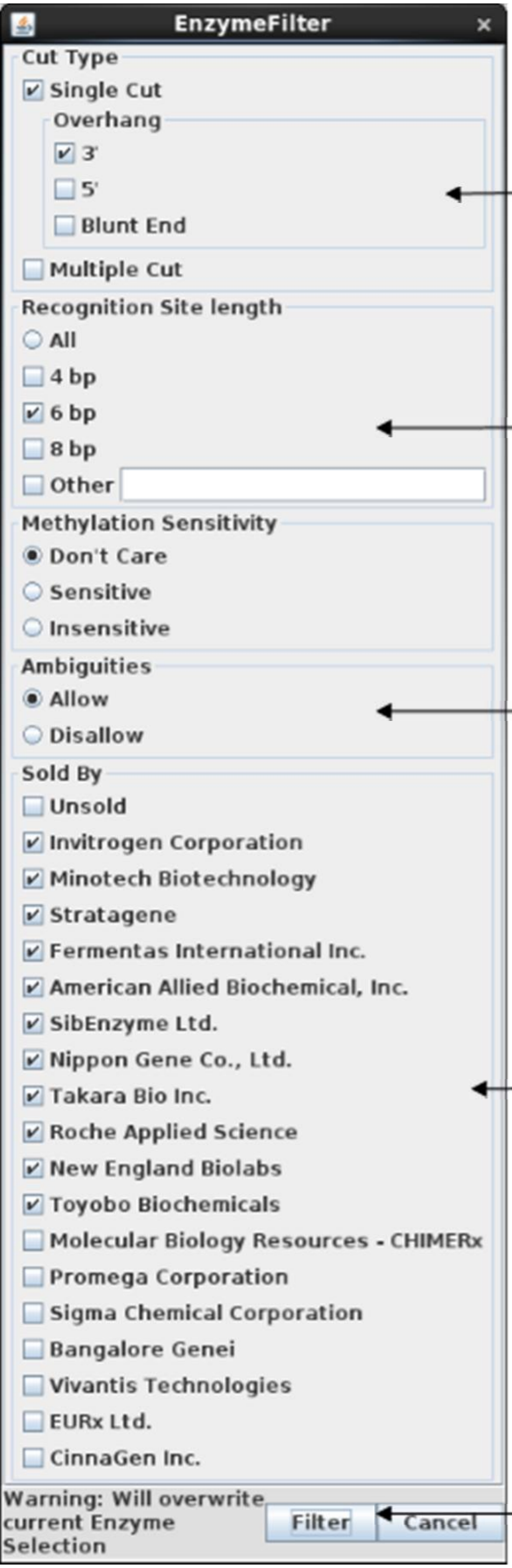

Select the desired overhang, and if enzymes with multiple cuts are allowed.

Specify the size of the recognition site. The other box can be used to type in a single size cut length. Cut length does not consider 'N' bases that are on either side of the recognition site.

When ambiguities are disallowed, only enzymes that have a recognition site consisting entirely of 'ATCG' are considered.

If Unsold is checked, enzymes that are not made by any of the below companies are considered. All other enzymes are considered if they are sold by at least one of the checked companies.

The filter can be applied by pressing the Filter button. This may uncheck enzymes already checked, and may check any enzymes currently unchecked.

After Processing

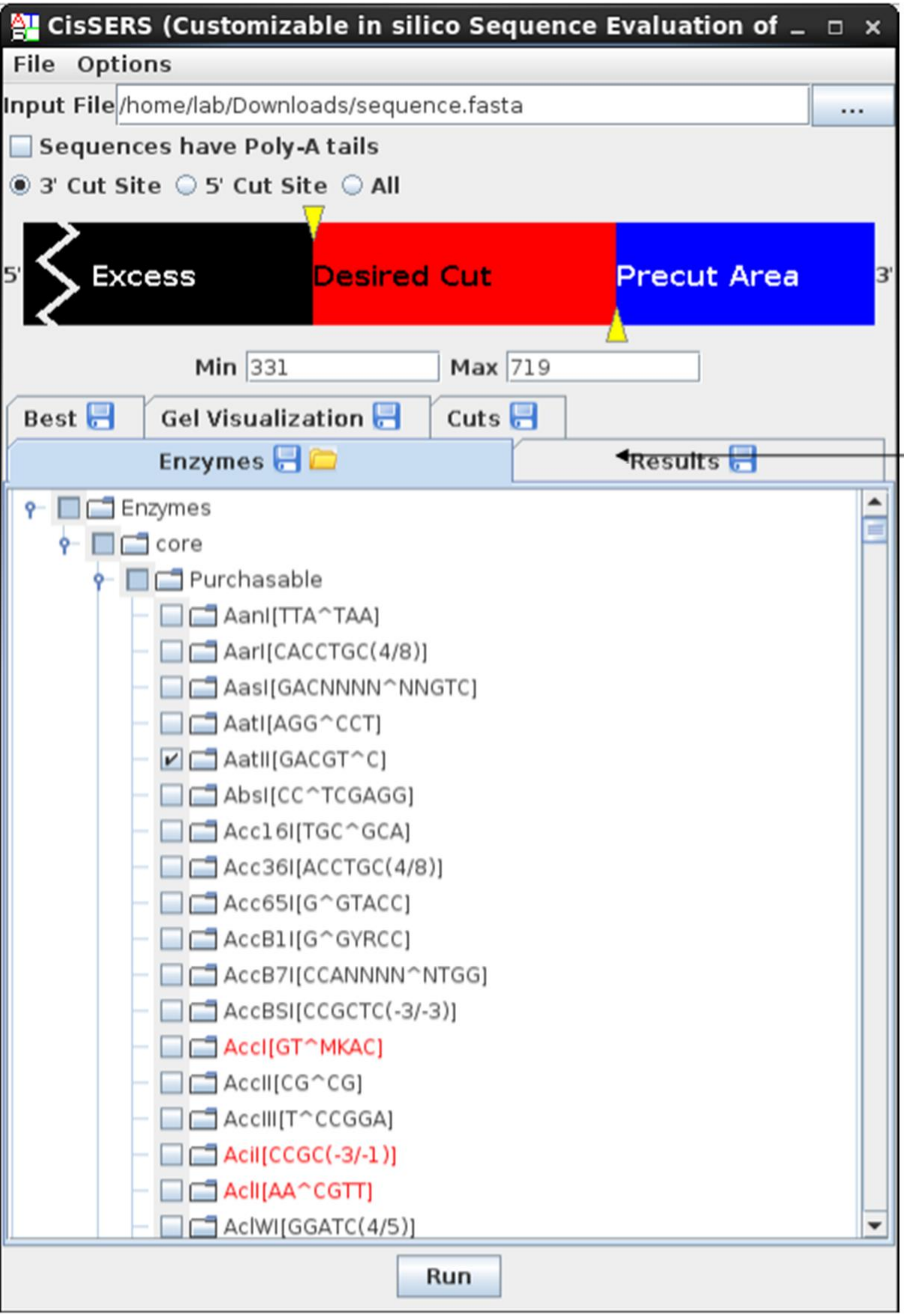

Additional tabs are added after processing.

Results

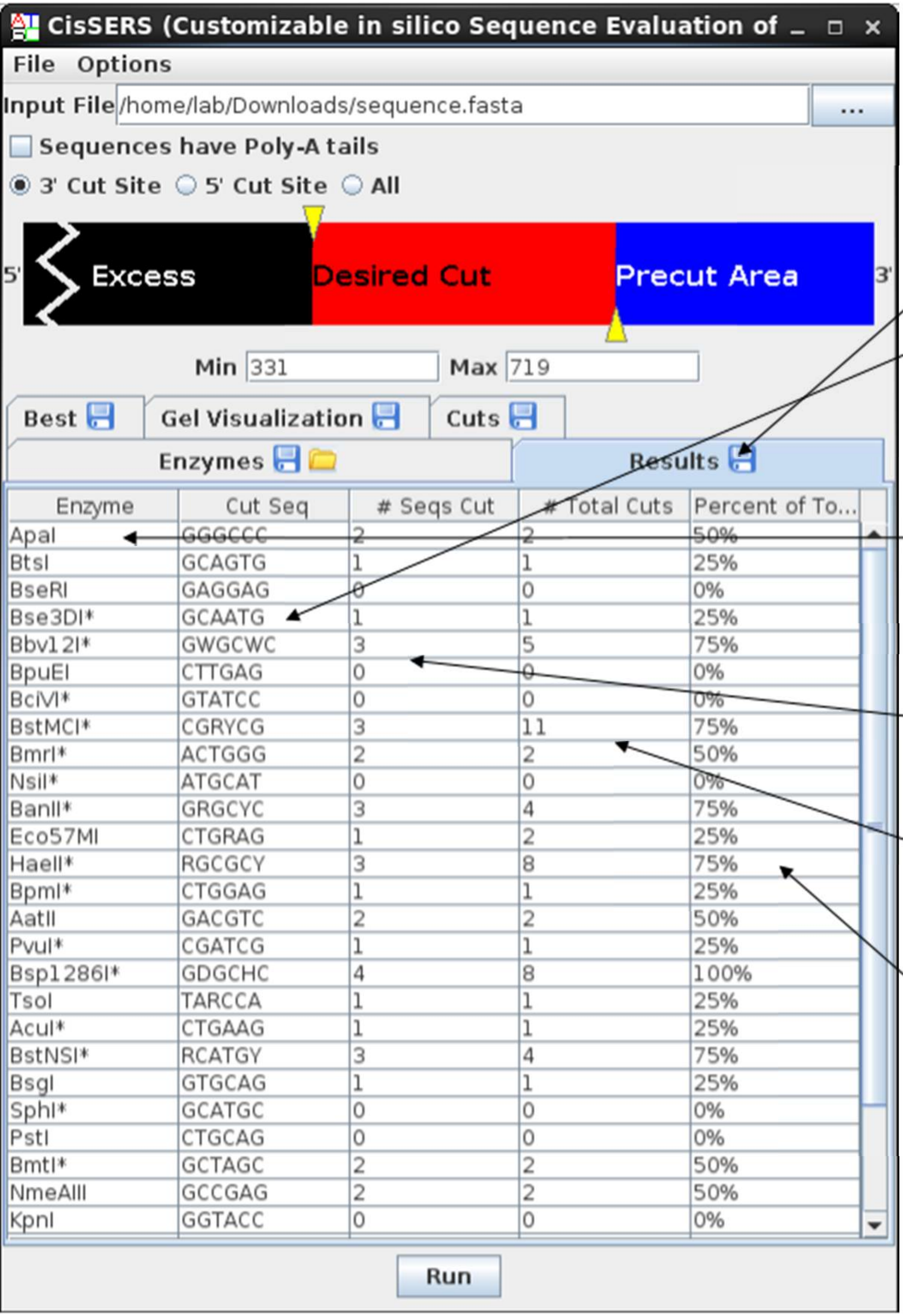

Enzyme	Cut Seq	# Seqs Cut	# Total Cuts	Percent of To...
ApaI	GGGCCC	2	2	50%
BtsI	GCAGTG	1	1	25%
BseRI	GAGGAG	0	0	0%
Bse3DI*	GCAATG	1	1	25%
Bbv12I*	GWGCWC	3	5	75%
BpuEI	CTTGAG	0	0	0%
BciVI*	GTATCC	0	0	0%
BstMCI*	CGRYCG	3	11	75%
BmrI*	ACTGGG	2	2	50%
NsiI*	ATGCAT	0	0	0%
BanII*	GRGCYC	3	4	75%
Eco57MI	CTGRAG	1	2	25%
HaeII*	RGCGCY	3	8	75%
BpmI*	CTGGAG	1	1	25%
AatII	GACGTC	2	2	50%
PvuI*	CGATCG	1	1	25%
Bsp1286I*	GDGCHC	4	8	100%
TsoI	TARCCA	1	1	25%
AcuI*	CTGAAG	1	1	25%
BstNSI*	RCATGY	3	4	75%
BsgI	GTGCAG	1	1	25%
SphI*	GCATGC	0	0	0%
PstI	CTGCAG	0	0	0%
BmtI*	GCTAGC	2	2	50%
NmeAIII	GCCGAG	2	2	50%
KpnI	GGTACC	0	0	0%

Save the results as a csv (comma separated value) file

Recognition site of the enzyme.

Name of the enzyme. A star after the name of the enzymes indicates that other enzymes share this recognition site.

Total number of sequences cut by this enzyme

Total number of times the enzyme cut among all sequences

Percentage of sequences cut at least once

Best

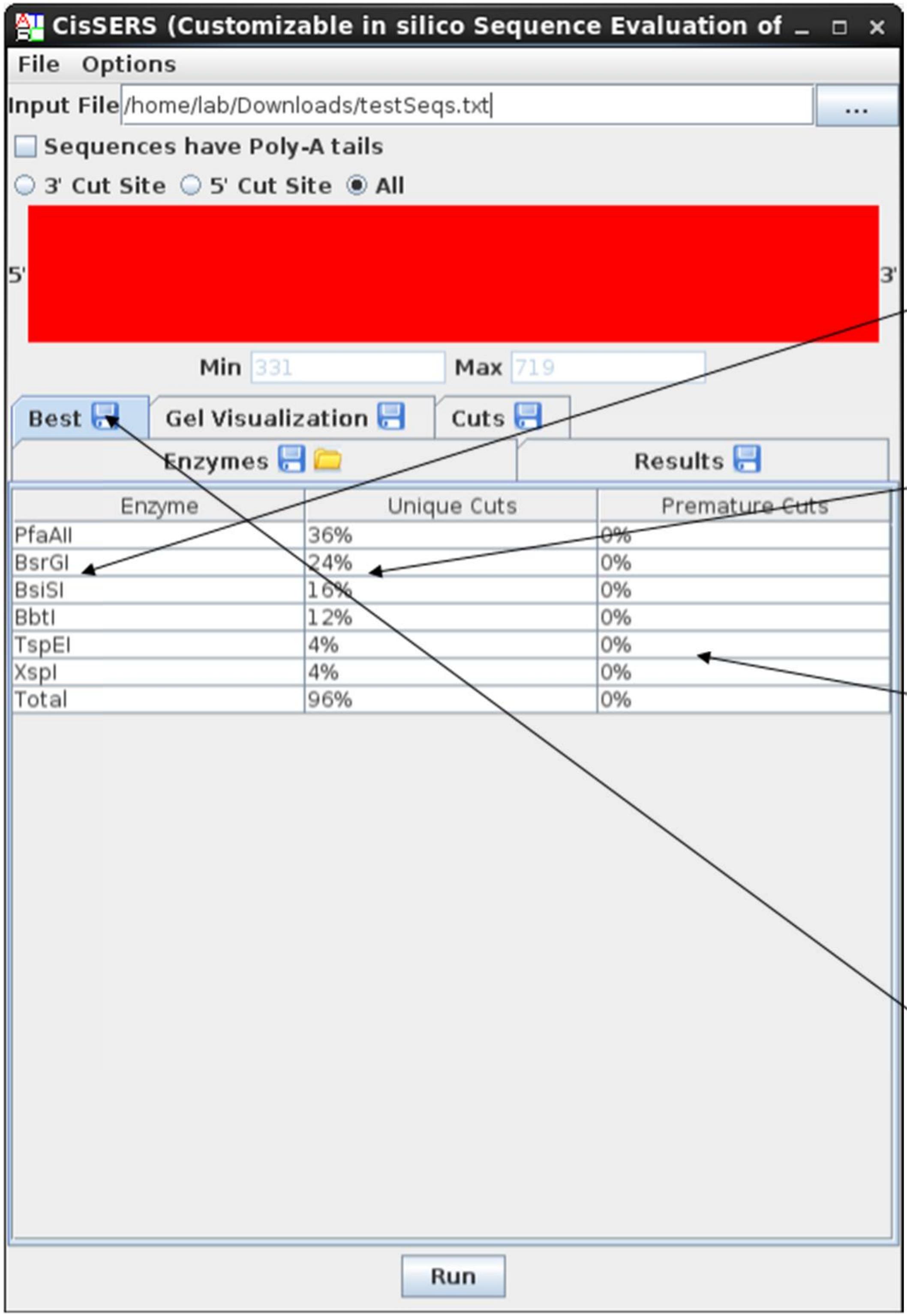

Enzyme Name

Percent of sequences cut by this enzyme that were not cut by above enzymes

Percent of sequences cut in the precut area that were not cut by the above enzymes

Save the results as a csv (comma separated value) file

Gel Visualization

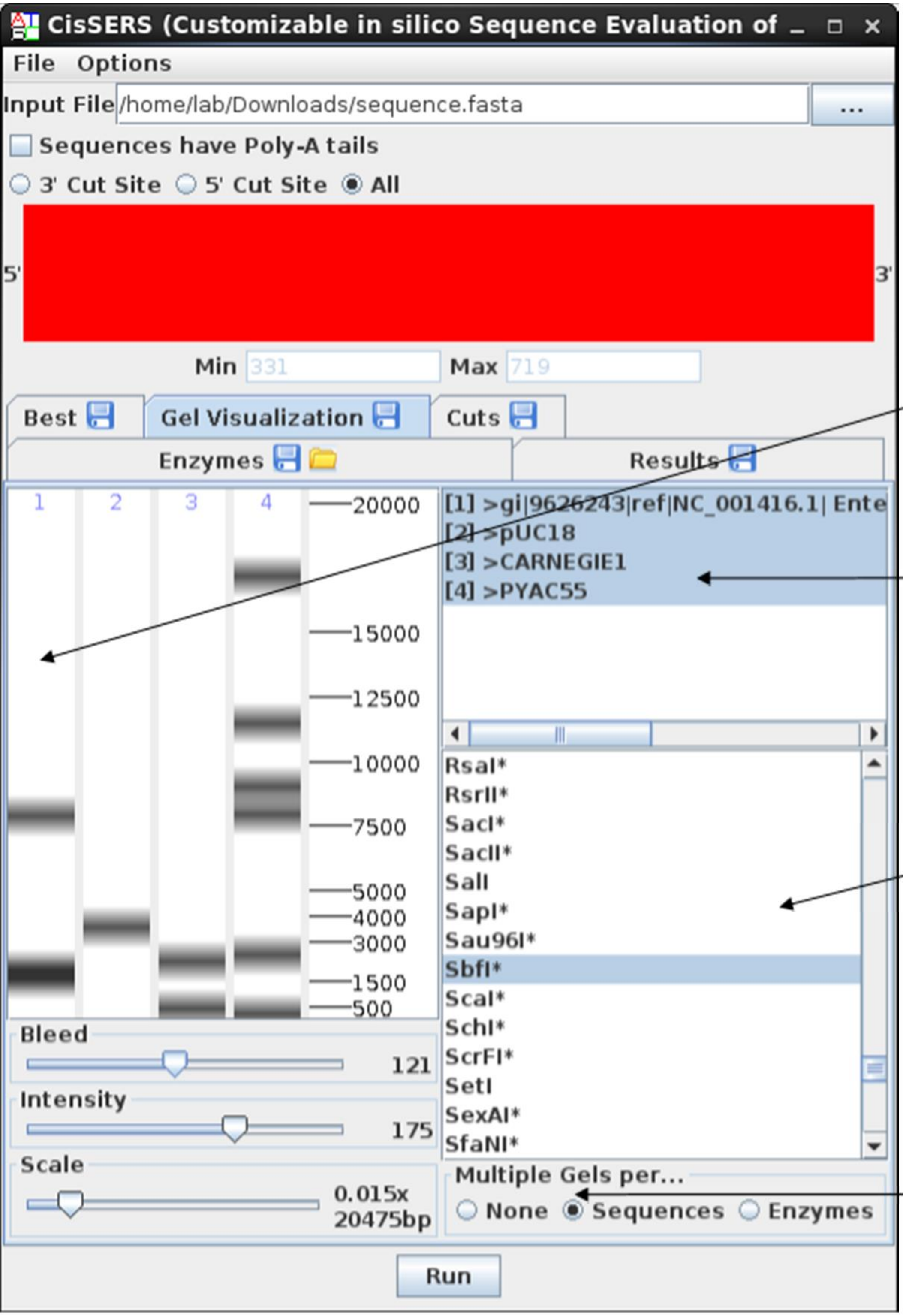

The Gel lane. The number at the top of the lane refers to number of the sequence in the list.

The list of sequences. Any selected will appear in the gel lane(s) to the left. Control click to select multiple.

The list of enzymes. Any selected enzyme will appear in the gel lanes(s) to the left. Control click to select multiple.

The gel mode. None means 1 gel for one sequence and one enzyme. Sequences means each lane refers to a single sequence. Enzymes means each lane refers to a different enzyme for a single sequence.

Gel Visualization 2

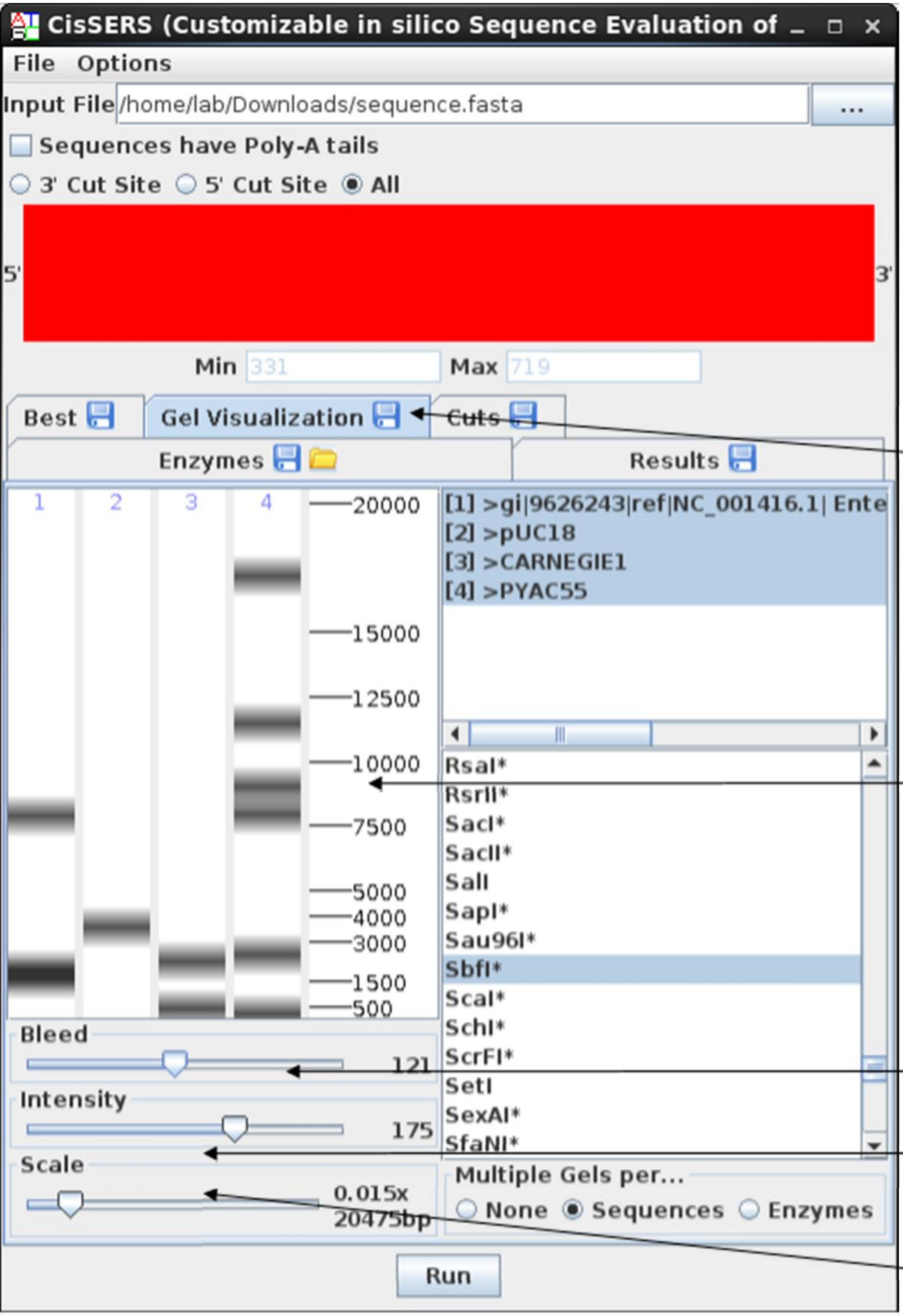

This saves the gel as an image to disk.

This shows the ladder for the gel.

This slider controls how think the bands are

This slider controls how dark the bands are

This slider controls how much zoom is on the bands
