# Supplementary material for: CisSERS: Customizable In Silico Sequence Evaluation for Restriction Sites
Source: PLoS One. 2016 Apr 12;11(4):e0152404. doi: 10.1371/journal.pone.0152404 (PMC4829253; doi:10.1371/journal.pone.0152404)
Supplement: S4 File — (PDF) [file pone.0152404.s004.pdf]

Supplementary Dataset 1: Galli Prepared sequences
>Macintosh 9
TATCCACGCCCTTCGTCGGCGCATCTCTGCCGTCCGTCTCCCGCCGTGGGTCCTACGCCCTCCCCTACCGCAGCACTGGACTCGC
CACCACCAGAATCAGGCTCAGCCTGCACGACTCCGTTCCTCCGATGAACCCCTTCGACCACTCGGCCGTCGACGTCGCCTCGCTT
TTGAGCCGCGCCGAGGGACTTCTTTACACTATTGCCGACGCCGCCGTGGCCGTCGACCCGAGCTCCACCGACGCCGCCGCTCAGA
AGAACGGCGGTTGGTTCGGCTTCATCTCAGACGCCATGGAGTTCGTGCTTAAGATTCTGAAAGGCGGACTTGAGACCGTGCACGT
GCCGTATGCATACGGCTTTGCAATTATACTGCTTACAATTATCGTCAAACTTGCCACATTGCCGCTCACAAAACAACAGGTGGAA
TCGACGTTAGCGATGCAAAACCTTCAACCAAAACTTAAGGCCATACAAAAGAGATACGAGGGCAATACGGAAAGAATACAACTTG
AGACATCACGGCTGTATCGGCAGGCAGGGGTTAATCCATTGGCAGGGTGTTTACCAACTTTGGCCAC
>Macintosh 2
TATCCACACCGTTCGTCGGCACATCTCTGCCGTCTGTCTCCCTCCGTGGGTCCTACACTCTCCCCTACCGCAGCAACGGACTCGC
CACCACCAGAATCAGGTTCAGCCTCCACGACTCCGTTCCTCCGATTAATCCCTTCGACCACTCCCCCGTCGACGTCGCCTCGCTT
TTGAGCCGCGCCGAGGGGCTTCTTTACACGATTGCCGACGCCGCCGTCGCCGTCGACCCGAGCTCCGCCGATGCTGCAGCTCAGA
AGAATGGCGGGTGGTTCGGCTTCATCTCCGACGCCATGGAGTTCGTGCTCAAGATTTTGAAAGGCGGGCTCGATGCCGTCCACGT
TCCGTATTCATACGGTTTTGCAATTATACTGCTTACGGTTATCGTTAAACTTGCCACACTGCCTCTCACAAAGCAACAGGTGGAA
TCGACGTTAGCGATGCAAAACCTTCAACCAAAACTTAAAGCCATACAAAAAAGATATGAGGGCAATACGGAAAGAATACAACTTG
AGACATCACGGCTGTATCGGCAGGCAGGGATTAATCCATTGGCAGGGTGTTTACCAACTTTGGCCAC
>Macintosh 5
TATCCACACCGTTCGTCGGCACATCTCTGCCGTCTGTCTCCCTCCGTGGGTCCTACACTCTCCCCTACCGCAGCAACGGACTCGC
CACCACCAGAATCAGGCTCAGCCTCCACGACTCCGTTCCTCCGATTAATCCCTTCGACCACTCCCCCGTCGACGTCGCCTCGCTT
TTGAGCCGCGCCGAGGGGCTTCTTTACACGATTGCCGACGCCGCCGTCGCCGTCGACCCGAGCTCCGCCGATGCTGCAGCTCAgA
AGAATGGCGGGTGGTTCGGCTTCATCTCCGACGCCATGGAGTTCGTGCTCAAGATTTTGAAAGGCGGGCTCGATGCCGTCCACGT
TCCGTATTCATACGGTTTTGCAATTATACTGCTTACGGTTATCGTTAAACTTGCCACACTGCCTCTCACAAAGCAACAGGTGGAA
TCGACGTTAGCGATGCAAAACCTTCAACCAAAACTTAAAGCCATACAAAAAAGATATGAGGGCAATACGGAAAGAATACAACTTG
AGACATCACGGCTGTATCGGCAGGCAGGGATTAATCCATTGGCAGGGTGTTTACCAACTTTGGCCAC
>Winesap 1
TATCCACACCGTTCGTCGGCACATCTCTGCCGTCTGTCTCCCGCCGTGGGTCCTACACTCTCCCCTACCGCAGCAACGGACTCGC
CACCACCAGAATCAGGTTCAGCCTCCACGACTCCGTTCCTCCGATTAATCCCTTCGACCACTCCCCCGTCGACGTCGCCTCGTTT
TTGAGCCGCGCCGAGGGGCTTCTTTACACGATTGCCGACGCCGCCGTCGCCGTCGACCCGAGCTCCGCCGATGCTGCGGCTCAGA
AGAATGGCGGGTGGTTCGGCTTCATCTCCGACGCCATGGAGTTCGTGCTCAAGATTTTGAAAGGCGGGCTCGATGCCGTGCACGT
TCCGTATTCATACGGTTTTGCAATTATACTGCTTACGGTTATCGTTAAACTTGCCACACTGCCTCTCACAAAGCAACAGGTGGAA
TCGACGTTAGCGATGCAAAACCTTCAACCAAAACTTAAAGCCATACAAAAAAGATATGAGGGCAATACGGAAAGAATACAACTTG
AGACATCACGGCTGTATCGGCAGGCAGGGATTAATCCATTGGCAGGGTGTTTACCAACTTTGGCCAC
>Winesap 3
TATCCACACCGTTCGTCGGCACATCTCTGCCGTCTGTCTCCCGCCGTGGGTCCTACACTCTCCCCTACCGCAGCAACGGACTCGC
CACCACCAGAATCAGGTTCAGCCTCCACGACTCCGTTCCTCCGATTAATCCCTTCGACCACTCCCCCGTCGACGTCGCCTCGTTT
TTGAGCCGCGCCGAGGGGCTTCTTTACACGATTGCCGACGCCGCTGTCGCCGTCNACCCGAGCTCCGCCGATGCTGCGGCTCATA
AGAATGGCGGGTGGTTCGGCTTCATCTCCGACGCCATGGAgTTCGTGCTCAAGATTTTGAAAGGCGGGCTCGATGCCGTGCACGT
TCCGTATTCATACGGTTTTGCAATTATACTGCTTACGGTTATCGTTAAACTTGCCACACTGCCTCTCACAAAGCAACAGGTGGAA
TCGACGTTAGCGATGCAAAACCTTCAACCAAAACTTAAAGCCATACAAAAAAGATATGAGGGCAATACGGAAAGAATACAACTTG
AGACATCACGGCTGtATCGGCAGGCaGGGATTAATCCATTGGCAGGGTGTTTACCAACTTTGGCCAC
>Red_Grav 4
TATCCACACCGTTCGTCGGCACATCTCTGCCGTCTGTCTCCCTCCGTGGGTCCTACACTCTCCCCTACCGCAGCAACGGACTCGC
CACCACCAGAATCAGGTTCAGCCTCCACGACTCCGTTCCTCCGATTAATCCCTTCGACCACTCCCCCGTCGACGTCGCCTCGCTT
TTGAGCCGCGCCGAGGGGCTTCTTTACACGATTGCCGACGCCGCCGTCGCCGTCGACCCGAGCTCCGCCGATGCTGCAGCTCAGA
AGAATGGCGGGTGGTTCGGCTTCATCTCCGACGCCATGGAGTTCGTGCTCAAGATTTTGAAAGGCGGGCTCGATGCCGTCCACGT
TCCGTATTCATACGGTTTTGCAATTATACTGCTTACGGTTATCGTTAAACTTGCCACACTGCCTCTCACAAAGCAACAGGTGGAA
TCGACGTTAGCGATGCAAAACCTTCAACCAAAACTTAAAGCCATACAAAAAAGATATGAGGGCAATACGGAAAGAATACAACTTG
AGACATCACGGCTGTATCGGCAGGCAGGGATTAATCCATTGGCAGGGTGTTTACCAACTTTGGCCAC
>Red_Grav 8
TATCCACACCGTTCGTCGGCACATCTCTGCCGTCTGTCTCCCTCCGTGGGTCCTACACTCTCCCCTACCGCAGCAACGGACTCGC
CACCACCAGAATCAGGTTCAGCCTCCACGACTCCGTTCCTCCGATTAATCCCTTCGACCACTCCCCCGTCGACGtCgCCTCGCTT
TTGAGCCGCGCCGAGGGGCTTCTTTACACGATTGCCGACGCCGCCGTCGCCGTCGACCCGAGCTCCGCCGATGCTGCAGCTCAGA
AGAATGGCGGGTGGTTCGGCTTCATCTCCGACGCCATGGAGTTCGTGCTCAAGATTTTGAAAGGCGGGCTCGATGCCGTCCACGT
TCCGTATTCATACGGTTTTGCaATTATACTGCTTACGGTTATCGTTAAACTTGCCACACTGCCTCTCACAAAGCAACAGGTGGAA
TCGACGTTAGCGATGCAAAACCTTCAACCAAAACTTAAAGCCATACAAAAAAGATATGAGGGCAATACGGAAAGAATACAACTTG
AGACATCACGGCTGTATCGGCAGGCAGGGATTAATCCATTGGCAGGGTGTTTACCAACTTTGGCCAC
>Red_Grav 10
TATCCACGCCCTTCGTCGGCGCATCTCTGCCGTCCGTCTCCCGCCGTGGGTCCTACGCCCTCCCCTACCGCAGCAGTGGACTCGC
CACCACCAGAATCAGGCTCAGCCTCCACGACTCCGTTCCTCCGATGAACCCCTTCGACCACTCGGCCGTCGACGTCGCCTCGCTT
TTGAGCCGCGCTGAGGGACTTCTTTACACTATTGCCGACGCCGCCGTGGCCGTCGACCCGAGCTCCACCGACGCCACCGCTCAGA
AGAACGGCGGTTGGTTCGGCTTCATCTCCGACGCCATGGAGTTCGTGCTTAAGATTCTGAAAGGCGGACTTGAGACCGTGCACGT
GCCGTATGCATACGGCTTTGCAATTATACTGCTTACAATTATCGTCAAACTTGCCACATTGCCGCTCACAAAACAACAGGTGGAA

TCGACGTTAGCGATGCAAAACCTTCAACCAAAACTTAAAGCCATACAAAAAAAGATATGAGGGCAATACGGAAAGAATACaACTT
GAGACATCACGGCTGTATCGGCAGGCAGGGATTAATCCATTGGCAGGGTGTTTACCAACTTTGGCCAC
>Haralson 2
TATCCACACCGTTCGTCGGCACATCTCTGCCGTCTGTCTCCCGCCGTGGGTCCTACACTCTCCCCTACCGCAGCAACGGACTCGC
CACCACCAGAATCAGGTTCAGCCTCCACGACTCCTTTCCTCCGATTAATCCCTTCGACCACTCCCCCGTCGACGTCGCCTCGCTT
TTGAGCCGCGCCGAGGGGCTTCTTTACACGATTGCCGACGCCGCCGTCGCCGTCGACCCGAGCTCCGCCGATGCTGCGGCTCAGG
AGAATGGCGGGTGGTTCGGCTTCATCTCCGACGCCATGGAGTTCGTGCTCAAGATTTTGAAAGGCGGACTCGATGCCGTGCACGT
TCCGTATTCATACGGTTTTGCAATTATACTGCTTACGGTTATCGTTAAACTTGCCACACTGCCTCTCACAAAGCAACAGGTGGAA
TCGACGTTAGCGATGCAAAACCTTCAACCAAAACTTAAAGCCATACAAAAAAGATATGAGGGCAATACGGAAAGAATACAACTTG
AGACATCACGGCTGTATCGGCAGGCAGGGATTAATCCATTGGCAGGGTGTTTACCAACTTTGGCCAC
>Haralson 5
TATCCACACCGTTCGTCGGCACATCTCTGCCGTCTGTCTCCCGCCGTGGGTCCTACACTCTCCCCTACCGCAGCAACGGACTCGC
CACCACCAGAATCAGGTTCAGCCTCCACGACTCCTTTCCTCCGATTAATCCCTTCGACCACTCCCCCGTCGACGTCGCCTCGCTT
TTGAGCCGCGCCGAGGGGCTTCTTTACACGATTGCCGACGCCGCCGTCGCCGTCGACCCGAGCTCCGCCGATGCTGCGGCTCAGA
AGAATGGCGGGTGGTTCGGCTTCATCTCCGACGCCATGGAGTTCGTGCTCAAGATTTTGAAAGGCGGACTCGACGCCGTGCaCGT
TCCGTACTCATACGGTTTTGCAATTATACTGCTTACGGTTATCgTTAAACTTGCCACACTGcCTCTCACAAAGCAACAGGTGGAA
TCGACGTTAGCGATGCAAAaCCTTCAACCAAAACTTAAAGCCATACAAAAAAGATATGAGGGCAATACGGAAAGAATACAACTTG
AgaCATCACGGCTGTATCGGCAGGCAGGGATTAATCCATTGGCAGGGTGTTTACcAACTTTGGNCAC
>Cox_Org 10
TATCCACGCCCTTCGTCGGCGCATCTCTGCCGTCCGTCTCCCGCCGTGGGTCCTACGCCCTCCCCTACCGCAGCACTGGACTCGC
CACCACCAGAATCAGGCTCAGCCTGCACGACTCCGTTCCTCCGGTGAACCCCTTCGACCACTCGGCCGTCGACGTCGCCTCGCTT
TTGAGCCGCGCCGAGGGACTTCTTTACACTATTGCCGACGCCGCCgTGGCCGTCGACCCGAGCTCCACCGACGCCGCCGCTCANA
AgAACGGCGGtTGGTTCGGCTTCaTCTCCGACGCCATGGAGTTCGTGCTTAAGATTCTGAAAGGCGGACTTGAGACCGTGCACGT
GCCGTATGCATACGGCTTTGCAATTATACTGCTTACAATTATCGTCAAACTTGCCACATTGTCGCTCACAAAACAACAGGTGGAA
TCGACGTTAGCGATGCAAAACCtTCaACCAAAACTtAAGGCCATACAAAAGAGATACGAGGGCAATACGGAAAGAATACAACTTG
AGACATCACGGCTGTATCGGCAGGCAGGGGTTAATCCATTGGCAGGGTGTTTACCAACTTTGGCCAC
>Cox_Org 5
TATCCACGCCCTTCGTCGGCGCATCTCTGCCGTCCGTCTCCCGCCGTGGGTCCTACGCCCTCCCCTACCGCAGCACTGGACTCGC
CACCACCAGAATCAGGCTCAGCCTGCACGACTCCGTTCCTCCGGTGAACCCCTTCGACCACTCGGCCGTCGACGTCGCCTCGCTT
TTGAGCCGCGCCGAGGGACTTCTTTACACTATTGCCGACGCCGCCGTGGCCGTCGACCCGAGCTCCACCGACGCCGCCGCTCAGA
AGAACGGCGGTTGGTTCGGCTTCATCTCCGACGCCATGGAGTTCGTGCTTAAGATTCTGAAAGGCGGACTTGAGACCGTGCACGT
GCCGTATGCATACGGCTTTGCAATTATACTGCTTACAATTATCGTCAAACTTGCCACATTGTCGCTCACAAAACAACAGGTGGAA
TCGACGTTAGCGATGCAAAACCTTCAACCAAAACTTAAGGCCATACAAAAGAGATACGAGGGCAATACGGAAAGAATACAACTTG
AGACATCACGGCTGTATCGGCAGGCAGGGGTTAATCCATTGGCAGGGTGTTTACCAACTTTGGCCAC
>Braeburn 10
TCATCCACGCCCTTCGTCGGCGCATCTCTGCCGTCCGTCTCCCGCCGTGGGTCCTACGCCCTCCCCTACCGCAGCAGTGGACTCG
CCACCACCAGAATCAGGCTCAGCCTCCACGACTCCGTTCCTCCGATGAACCCCTTCGACCACTCGGCCGTCGACGTCGCCTCGCT
TTTGAGCCGCGCTGAGGGACTTCTTTACACTATTGCCGACGCCGCCGTGGCCGTCGACCCGAGCTCCACCGACGCCGCCGCTCAG
AAGAACGGCGGTTGGTTCGGCTTCATCTCCGACGCCATGGAGTTCGTGCTTAAGATTCTGAAAGGCGGACTTGAGACCGTGCACG
TGCCGTATGCATACGGCTTTGCAATTATACTGCTTACAATTATCGTCAAACTTGCCACATTGCCGCTCACAAAACAACAGGTGGA
ATCGACGTTAGCGATGCAAGACCTTCAACCAAAACTTAAGGCCATACAAAAGAGATACGAGGGCAATACGGAAAGAATACAACTT
GAGACATCACGGCTGTATCGGCAGGCAGGGGTTAATCCATTGGCAGGGTGTTTACCAACTTTGGCCAC
>Honeycrisp 3
TATCCACACCGTTCGTCGGCACATCTCTGCCGTCTGTCTCCCTCCGTGGGTCCTACACTCTCCCCTACCGCAGCAACGGACTCGC
CACCACCAGAATCAGGTTCAGCCTCCACGACTCCGTTCCTCCGATTAATCCCTTCGACCACTCCCCCGTCGACGTCGCCTCGCTT
TTGAGCCGCGCCGAGGGGCTTCTTTACACGATTGCCGACGCCGCCGTCGCCGTCGACCCGAGCTCCGCCGATGCTGCAGCTCAGA
AGAATGGCGGGTGGTTCGGCTTCATCTCCGACGCCATGGAGTTCGTGCTCAAGATTTTGAAAGGCGGGCTCGATGCCGTGCACGT
TCCGTATTCATACGGTTTTGCAATTATACTGCTTACGGTTATCGTTAAACTTGCCACACTGCCTCTCACAAAGCAACAGGTGGAA
TCGACGTTAGCGATGCAAAACCCTCAACCAAAACTTAAAGCCATACAAAAAAGATATGAGGGCAATACGGAAAGAATACAACTTG
AGACATCACGGCTGTATCGGCAGGCAGGGATTAATCCATTGGCAGGGTGTTTACCAACTTTGGCCAC
>Gold_Del
TATCCACGCCGTTCGTCGGCACATCTCTGCCGTCTGTCTCCCTCCGTGGGTCCTACACTCTCCCCTACCGCAGCAACGGACTCGC
CACCACCAGAATCAGGTTCAGCCTCCACGACTCCGTTCCTCCGATTAATCCCTTCGACCACTCCCCCGTCGACGTCGCCTCGCTT
TTGAGCCGCGCCGAGGGGCTTCTTTACACGATTGCCGACGCCGCCGTCGCCGTCGACCCGAGCTCCGCCGATGCTGCAGCTCAGA
AGAATGGCGGGTGGTTCGGCTTCATCTCCGACGCCATGGAGTTCGTGCTCAAGATTTTGAAAGGCGGGCTCGATGCCGTGCACGT
TCCGTATTCATACGGTTTTGCAATTATACTGCTTACGGTTATCGTTAAACTTGCCACACTGCCTCTCACAAAGCAACAGGTGGAA
TCGACGTTAGCGATGCAAAACCTTCAACCAAAACTTAAAGCCATACAAAAAAGATATGAGGGCAATACGGAAAGAATACAACTTG
AGACATCACGGCTGTATCGGCAGGCAGGGATTAATCCATTGGCAGGGTGTTTACCAACTTTGGCCAC

Supplementary Dataset 2: Arabidopsis atpc1 wild type (wt) and cfq mutant
>wt
AACAACAATGTGGGTTTCATCAAAACCATCTCTTTCTGCTGATTCATCTTCCTTATCATTCCGATCTGTTCTCAAGTGCCCAACT
AACACTTCCTCACCTCCTTCACGAGCTTCCTCTGTTTCACCACTCCAAGCGTCTCTTCGTGAGCTCAGAGACCGTATCGATTCAG
TCAAAAACACTCAAAAGATCACCGAAGCTATGAAGCTTGTCGCTGCAGCTAAAGTCAGGAGAGCTCAAGAAGCTGTTGTCAATGG
ACGACCATTCTCAGAAACCCTAGTTGAAGTTCTTTACAACATCAACGAACAGCTTCAAACCGATGATGTCGATGTTCCCTTAACC
AAAGTCAGACCGGTTAAGAAAGTAGCTCTCGTTGTCGTCACCGGTGATCGTGGATTATGTGGTGGATTCAACAATTTCATCATTA
AGAAAGCAGAGGCAAGAATCAAAGAGCTTAAAGGTCTAGGTCTTGAATACACAGTCATTAGCGTGGGCAAGAAGGGAAATTCTTA
TTTCCTCCGTCGCCCGTACATCCCCGTCGACAAATACCTAGAAGCCGGAACTTTACCTACGGCTAAAGAAGCTCAAGCTGTGGCT
GATGATGTCTTCTCTCTGTTTATAAGTGAAGAAGTCGACAAAGTCGAGCTCTTGTACACAAAGTTTGTCTCTTTGGTCAAATCAG
AACCCGTGATCCACACGCTACTGCCTTTATCACCTAAAGGAGAGATCTGTGACATTAATGGAACCTGTGTGGATGCTGCGGAAGA
TGAGTTTTTCAGGTTAACGACAAAAGAAGGGAAATTGACAGTTGAAAGAGAGACTTTTAGGACACCAACAGCTGATTTCTCGCCG
ATCTTGCAATTCGAGCAAGACCCTGTTCAGATTCTTGATGCTTTGTTGCCTCTGTATCTTAACAGTCAGATTCTTAGGGCATTAC
AGGAGTCATTGGCTAGTGAGCTTGCAGCTAGAATGAGTGCAATGAGTAGTGCTTCGGATAATGCATCGGATCTCAAGAAATCGCT
TTCGATGGTGTATAATAGAAAGCGTCAAGCTAAGATTACTGGAGAGATTCTTGAGATTGTTGCTGGAGCTAATGCAC
>cfq
AACAACAATGTGGGTTTCATCAAAACCATCTCTTTCTGCTGATTCATCTTCCTTATCATTCCGATCTGTTCTCAAGTGCCCAACT
AACACTTCCTCACCTCCTTCACGAGCTTCCTCTGTTTCACCACTCCAAGCGTCTCTTCGTGAGCTCAGAGACCGTATCGATTCAG
TCAAAAACACTCAAAAGATCACCGAAGCTATGAAGCTTGTCGCTGCAGCTAAAGTCAGGAGAGCTCAAGAAGCTGTTGTCAATGG
ACGACCATTCTCAGAAACCCTAGTTGAAGTTCTTTACAACATCAACGAACAGCTTCAAACCGATGATGTCGATGTTCCCTTAACC
AAAGTCAGACCGGTTAAGAAAGTAGCTCTCGTTGTCGTCACCGGTGATCGTGGATTATGTGGTGGATTCAACAATTTCATCATTA
AGAAAGCAGAGGCAAGAATCAAAGAGCTTAAAGGTCTAGGTCTTGAATACACAGTCATTAGCGTGGGCAAGAAGGGAAATTCTTA
TTTCCTCCGTCGCCCGTACATCCCCGTCGACAAATACCTAGAAGCCGGAACTTTACCTACGGCTAAAGAAGCTCAAGCTGTGGCT
GATGATGTCTTCTCTCTGTTTATAAGTGAAGAAGTCGACAAAGTCGAGCTCTTGTACACAAAGTTTGTCTCTTTGGTCAAATCAG
AACCCGTGATCCACACGCTACTGCCTTTATCACCTAAAGGAGAGATCTGTGACATTAATGGAACCTGTGTGGATGCTGCGGAAGA
TGAGTTTTTCAGGTTAACGACAAAAGAAGGGAAATTGACAGTTGAAAGAGAGACTTTTAGGACACCAACAGCTGATTTCTCGCCG
ATCTTGCAATTCAAGCAAGACCCTGTTCAGATTCTTGATGCTTTGTTGCCTCTGTATCTTAACAGTCAGATTCTTAGGGCATTAC
AGGAGTCATTGGCTAGTGAGCTTGCAGCTAGAATGAGTGCAATGAGTAGTGCTTCGGATAATGCATCGGATCTCAAGAAATCGCT
TTCGATGGTGTATAATAGAAAGCGTCAAGCTAAGATTACTGGAGAGATTCTTGAGATTGTTGCTGGAGCTAATGCAC
