# Supplementary material for: CisSERS: Customizable In Silico Sequence Evaluation for Restriction Sites
Source: PLoS One. 2016 Apr 12;11(4):e0152404. doi: 10.1371/journal.pone.0152404 (PMC4829253; doi:10.1371/journal.pone.0152404)
Supplement: S5 File — (ZIP) [file pone.0152404.s005.zip › CisSERS/Enzymes.txt.docx]

*Stratagene E

*Fermentas International Inc. F

*Invitrogen Corporation B

*Minotech Biotechnology C

*Roche Applied Science M

*New England Biolabs N

*Toyobo Biochemicals O

*American Allied Biochemical, Inc. H

*SibEnzyme Ltd. I

*Nippon Gene Co., Ltd. J

*Takara Bio Inc. K

*Bangalore Genei U

*Vivantis Technologies V

*Molecular Biology Resources - CHIMERx Q

*Sigma Chemical Corporation S

*Promega Corporation R

*CinnaGen Inc. Y

*EURx Ltd. X

+Enzymes

+core

+Purchasable

AanI TTA^TAA F false false

AarI CACCTGC(4/8) F false false

AasI GACNNNN^NNGTC F false false

AatI AGG^CCT O false false

AatII GACGT^C FIKMNORV false false

AbsI CC^TCGAGG I false false

Acc16I TGC^GCA IV false false

Acc36I ACCTGC(4/8) I false false

Acc65I G^GTACC FINRV false false

AccB1I G^GYRCC IV false false

AccB7I CCANNNN^NTGG IRV false false

AccBSI CCGCTC(-3/-3) IV false false

AccI GT^MKAC BJKMNOQRSUX true false

AccII CG^CG JK false false

AccIII T^CCGGA JKR false false

AciI CCGC(-3/-1) N true false

AclI AA^CGTT INV true false

AclWI GGATC(4/5) I false false

AcoI Y^GGCCR I false false

AcsI R^AATTY IV false false

AcuI CTGAAG(16/14) IN true false

AcvI CAC^GTG QX false false

AcyI GR^CGYC J false false

AdeI CACNNN^GTG F false false

AfaI GT^AC K false false

AfeI AGC^GCT IN false false

AfiI CCNNNNN^NNGG V false false

AflII C^TTAAG JKN false false

AflIII A^CRYGT MN true false

AgeI A^CCGGT JNR true false

AgsI TTS^AA I false false

AhdI GACNNN^NNGTC N false false

AhlI A^CTAGT IV false false

AjiI CACGTC(-3/-3) F false false

AjnI ^CCWGG I true false

AjuI (7/12)GAANNNNNNNTTGG(11/6) F false false

AleI CACNN^NNGTG N false false

AlfI (10/12)GCANNNNNNTGC(12/10) F false false

AloI (7/12)GAACNNNNNNTCC(12/7) F true false

AluBI AG^CT I false false

AluI AG^CT BCFHIJKMNOQRSUVXY true false

Alw21I GWGCW^C F false false

Alw26I GTCTC(1/5) F true false

Alw44I G^TGCAC FJO false false

AlwI GGATC(4/5) N true false

AlwNI CAGNNN^CTG N false false

Ama87I C^YCGRG IV false false

Aor13HI T^CCGGA K false false

Aor51HI AGC^GCT K false false

ApaI GGGCC^C BFIJKMNOQRSUVX true false

ApaLI G^TGCAC CKNU true false

ApeKI G^CWGC N true false

ApoI R^AATTY N true false

ArsI (8/13)GACNNNNNNTTYG(11/6) I false false

AscI GG^CGCGCC N true false

AseI AT^TAAT JNO true false

AsiGI A^CCGGT IV false false

AsiSI GCGAT^CGC IN true false

Asp700I GAANN^NNTTC M false false

Asp718I G^GTACC M false false

AspA2I C^CTAGG IV false false

AspEI GACNNN^NNGTC M false false

AspI GACN^NNGTC M false false

AspLEI GCG^C IV false false

AspS9I G^GNCC IV true false

AssI AGT^ACT U false false

AsuC2I CC^SGG I false false

AsuHPI GGTGA(8/7) IV false false

AsuII TT^CGAA C false false

AsuNHI G^CTAGC IV false false

AvaI C^YCGRG BJMNOQRUX true false

AvaII G^GWCC JKMNRY true false

AviII TGC^GCA M false false

AvrII C^CTAGG N false false

AxyI CC^TNAGG J false false

BaeGI GKGCM^C N true false

BaeI (10/15)ACNNNNGTAYC(12/7) N false false

BalI TGG^CCA JKQRX true false

BamHI G^GATCC BCFHIJKMNOQRSUVXY true false

BanI G^GYRCC NORU true false

BanII GRGCY^C KNORX true false

BanIII AT^CGAT O false false

BarI (7/12)GAAGNNNNNNTAC(12/7) I false false

BasI CCANNNN^NTGG U false false

BauI CACGAG(-5/-1) F false false

BbrPI CAC^GTG MO false false

BbsI GAAGAC(2/6) N false false

BbuI GCATG^C R false false

Bbv12I GWGCW^C IV false false

BbvCI CCTCAGC(-5/-2) N true false

BbvI GCAGC(8/12) N true false

BccI CCATC(4/5) N false false

BceAI ACGGC(12/14) N true false

BcgI (10/12)CGANNNNNNTGC(12/10) N true false

BciT130I CC^WGG K false false

BciVI GTATCC(6/5) N false false

BclI T^GATCA CFJMNORSUY true false

BcnI CC^SGG FK true false

BcoDI GTCTC(1/5) N false false

BcuI A^CTAGT F false false

BdaI (10/12)TGANNNNNNTCA(12/10) F false false

BfaI C^TAG N false false

BfiI ACTGGG(5/4) F true false

BfmI C^TRYAG F false false

BfoI RGCGC^Y F false false

BfrI C^TTAAG MO false false

BfuAI ACCTGC(4/8) N true false

BfuCI ^GATC N true false

BfuI GTATCC(6/5) F false false

BglI GCCNNNN^NGGC CFHIJKMNOQRUVXY true false

BglII A^GATCT BCFHIJKMNOQRSUVXY true false

BisI GC^NGC I false false

BlnI C^CTAGG KMS false false

BlpI GC^TNAGC N true false

BlsI GCN^GC I false false

BmcAI AGT^ACT V false false

Bme1390I CC^NGG F false false

Bme18I G^GWCC IV false false

BmeRI GACNNN^NNGTC V false false

BmeT110I C^YCGRG K false false

BmgBI CACGTC(-3/-3) N true false

BmgT120I GG^NCC K false false

BmiI GGN^NCC V false false

BmrFI CC^NGG V false false

BmrI ACTGGG(5/4) N false false

BmsI GCATC(5/9) F false false

BmtI GCTAG^C INV true false

BmuI ACTGGG(5/4) I false false

BoxI GACNN^NNGTC F false false

BpiI GAAGAC(2/6) F false false

BplI (8/13)GAGNNNNNCTC(13/8) F false false

BpmI CTGGAG(16/14) IN true false

Bpu10I CCTNAGC(-5/-2) FINV true false

Bpu1102I GC^TNAGC FK false false

Bpu14I TT^CGAA IV false false

BpuAI GAAGAC(2/6) M false false

BpuEI CTTGAG(16/14) N true false

BpuMI CC^SGG V false false

BpvUI CGAT^CG V false false

Bsa29I AT^CGAT I false false

BsaAI YAC^GTR N true false

BsaBI GATNN^NNATC N false false

BsaHI GR^CGYC N true false

BsaI GGTCTC(1/5) N true false

BsaJI C^CNNGG N true false

BsaMI GAATGC(1/-1) R false false

BsaWI W^CCGGW N true false

BsaXI (9/12)ACNNNNNCTCC(10/7) N false false

Bsc4I CCNNNNN^NNGG I true false

Bse118I R^CCGGY IV false false

Bse1I ACTGG(1/-1) IV false false

Bse21I CC^TNAGG IV false false

Bse3DI GCAATG(2/0) IV false false

Bse8I GATNN^NNATC IV false false

BseAI T^CCGGA CM false false

BseBI CC^WGG C false false

BseCI AT^CGAT C true false

BseDI C^CNNGG F false false

BseGI GGATG(2/0) F false false

BseJI GATNN^NNATC F false false

BseLI CCNNNNN^NNGG F false false

BseMI GCAATG(2/0) F false false

BseMII CTCAG(10/8) F true false

BseNI ACTGG(1/-1) F false false

BsePI G^CGCGC IV false false

BseRI GAGGAG(10/8) N true false

BseSI GKGCM^C F false false

BseX3I C^GGCCG IV false false

BseXI GCAGC(8/12) F false false

BseYI CCCAGC(-5/-1) N false false

BsgI GTGCAG(16/14) N true false

Bsh1236I CG^CG F false false

Bsh1285I CGRY^CG F false false

BshFI GG^CC C false false

BshNI G^GYRCC F false false

BshTI A^CCGGT F false false

BshVI AT^CGAT V false false

BsiEI CGRY^CG N false false

BsiHKAI GWGCW^C N true false

BsiHKCI C^YCGRG QX false false

BsiSI C^CGG C false false

BsiWI C^GTACG MNO false false

BslFI GGGAC(10/14) I false false

BslI CCNNNNN^NNGG N true false

BsmAI GTCTC(1/5) N true false

BsmBI CGTCTC(1/5) N true false

BsmFI GGGAC(10/14) N true false

BsmI GAATGC(1/-1) JMNOS false false

BsnI GG^CC V false false

Bso31I GGTCTC(1/5) IV false false

BsoBI C^YCGRG N true false

Bsp119I TT^CGAA F false false

Bsp120I G^GGCCC F false false

Bsp1286I GDGCH^C JKNR true false

Bsp13I T^CCGGA IV false false

Bsp1407I T^GTACA FK false false

Bsp143I ^GATC F false false

Bsp1720I GC^TNAGC IV false false

Bsp19I C^CATGG IV false false

Bsp68I TCG^CGA F false false

BspACI CCGC(-3/-1) I true false

BspCNI CTCAG(9/7) N true false

BspDI AT^CGAT N false false

BspEI T^CCGGA N false false

BspFNI CG^CG I false false

BspHI T^CATGA N true false

BspLI GGN^NCC F false false

BspMI ACCTGC(4/8) N true false

BspOI GCTAG^C F false false

BspPI GGATC(4/5) F false false

BspQI GCTCTTC(1/4) N false false

BspT104I TT^CGAA K false false

BspT107I G^GYRCC K false false

BspTI C^TTAAG F false false

BsrBI CCGCTC(-3/-3) N true false

BsrDI GCAATG(2/0) N false false

BsrFI R^CCGGY N true false

BsrGI T^GTACA N false false

BsrI ACTGG(1/-1) N true false

BsrSI ACTGG(1/-1) R false false

BssAI R^CCGGY C false false

BssECI C^CNNGG I false false

BssHII G^CGCGC JKMNOQRSX true false

BssKI ^CCNGG N true false

BssMI ^GATC V false false

BssNAI GTA^TAC IV false false

BssNI GR^CGYC V false false

BssSI CACGAG(-5/-1) N true false

BssT1I C^CWWGG IV false false

Bst1107I GTA^TAC FKM false false

Bst2BI CACGAG(-5/-1) IV false false

Bst2UI CC^WGG IV false false

Bst4CI ACN^GT IV false false

Bst6I CTCTTC(1/4) IV false false

Bst98I C^TTAAG R false false

BstACI GR^CGYC I false false

BstAFI C^TTAAG I false false

BstAPI GCANNNN^NTGC IN false false

BstAUI T^GTACA IV false false

BstBAI YAC^GTR IV false false

BstBI TT^CGAA N false false

BstC8I GCN^NGC I false false

BstDEI C^TNAG IV false false

BstDSI C^CRYGG IV false false

BstEII G^GTNACC CHJMNORSU false false

BstENI CCTNN^NNNAGG IV false false

BstF5I GGATG(2/0) IV true false

BstFNI CG^CG IV false false

BstH2I RGCGC^Y IV false false

BstHHI GCG^C IV false false

BstKTI GAT^C I false false

BstMAI GTCTC(1/5) IV false false

BstMBI ^GATC IV false false

BstMCI CGRY^CG IV false false

BstMWI GCNNNNN^NNGC I false false

BstNI CC^WGG N true false

BstNSI RCATG^Y IV false false

BstOI CC^WGG R false false

BstPAI GACNN^NNGTC IV false false

BstPI G^GTNACC K false false

BstSCI ^CCNGG I false false

BstSFI C^TRYAG I false false

BstSLI GKGCM^C I false false

BstSNI TAC^GTA IV false false

BstUI CG^CG N true false

BstV1I GCAGC(8/12) I false false

BstV2I GAAGAC(2/6) IV false false

BstX2I R^GATCY IV false false

BstXI CCANNNNN^NTGG FHIJKMNOQRVX false false

BstYI R^GATCY N true false

BstZ17I GTA^TAC N false false

BstZI C^GGCCG R false false

Bsu15I AT^CGAT F true false

Bsu36I CC^TNAGG NR false false

BsuI GTATCC(6/5) I false false

BsuRI GG^CC FI true false

BsuTUI AT^CGAT X false false

BtgI C^CRYGG N false false

BtgZI GCGATG(10/14) N true false

BtrI CACGTC(-3/-3) IV false false

BtsCI GGATG(2/0) N false false

BtsI GCAGTG(2/0) N false false

BtsIMutI CAGTG(2/0) N false false

BtuMI TCG^CGA V false false

BveI ACCTGC(4/8) F false false

Cac8I GCN^NGC N true false

CaiI CAGNNN^CTG F false false

CciI T^CATGA I false false

CciNI GC^GGCCGC IV false false

CelII GC^TNAGC M false false

CfoI GCG^C MRS false false

Cfr10I R^CCGGY FKO true false

Cfr13I G^GNCC FO true false

Cfr42I CCGC^GG F true false

Cfr9I C^CCGGG FO true false

CfrI Y^GGCCR F true false

ClaI AT^CGAT BHKMNQRSU true false

CpoI CG^GWCCG FK false false

CseI GACGC(5/10) F false false

CsiI A^CCWGGT F false false

Csp45I TT^CGAA O false false

Csp6I G^TAC F false false

CspAI A^CCGGT C false false

CspCI (11/13)CAANNNNNGTGG(12/10) N true false

CspI CG^GWCCG OR false false

CviAII C^ATG N true false

CviJI RG^CY QX true false

CviKI-1 RG^CY N false false

CviQI G^TAC N true false

DdeI C^TNAG BMNOQRSX true false

DinI GGC^GCC V false false

DpnI GA^TC BEFKMNOQRSX false false

DpnII ^GATC N true false

DraI TTT^AAA BFIJKMNOQRSUVXY true false

DraII RG^GNCCY M false false

DraIII CACNNN^GTG IMNV true false

DrdI GACNNNN^NNGTC N false false

DriI GACNNN^NNGTC I false false

DseDI GACNNNN^NNGTC IV false false

EaeI Y^GGCCR KN true false

EagI C^GGCCG N true false

Eam1104I CTCTTC(1/4) F false false

Eam1105I GACNNN^NNGTC FK false false

EarI CTCTTC(1/4) N true false

EciI GGCGGA(11/9) N false false

Ecl136II GAG^CTC F false false

EclXI C^GGCCG MS false false

Eco105I TAC^GTA FO false false

Eco130I C^CWWGG F false false

Eco147I AGG^CCT F false false

Eco24I GRGCY^C F false false

Eco31I GGTCTC(1/5) F true false

Eco32I GAT^ATC F false false

Eco47I G^GWCC FO false false

Eco47III AGC^GCT FMOR false false

Eco52I C^GGCCG FKO false false

Eco53kI GAG^CTC N false false

Eco57I CTGAAG(16/14) F true false

Eco57MI CTGRAG(16/14) F false false

Eco72I CAC^GTG F false false

Eco81I CC^TNAGG FKO false false

Eco88I C^YCGRG F false false

Eco91I G^GTNACC F false false

EcoICRI GAG^CTC IRV false false

EcoNI CCTNN^NNNAGG N false false

EcoO109I RG^GNCCY FJKN true false

EcoO65I G^GTNACC K false false

EcoRI G^AATTC BCFHIJKMNOQRSUVXY true false

EcoRII ^CCWGG FJMO true false

EcoRV GAT^ATC BCHIJKMNOQRSUVXY true false

EcoT14I C^CWWGG K false false

EcoT22I ATGCA^T KO false false

EcoT38I GRGCY^C J true false

EgeI GGC^GCC I false false

EheI GGC^GCC FO false false

ErhI C^CWWGG IV false false

Esp3I CGTCTC(1/5) F true false

FaeI CATG^ I false false

FaiI YA^TR I false false

FalI (8/13)AAGNNNNNCTT(13/8) I false false

FaqI GGGAC(10/14) F false false

FatI ^CATG IN true false

FauI CCCGC(4/6) IN true false

FauNDI CA^TATG IV false false

FbaI T^GATCA K false false

FblI GT^MKAC IV false false

Fnu4HI GC^NGC N true false

FokI GGATG(9/13) IJKMNVX true false

FriOI GRGCY^C IV false false

FseI GGCCGG^CC N true false

Fsp4HI GC^NGC I true false

FspAI RTGC^GCAY F false false

FspBI C^TAG F false false

FspEI CC(12/16) N false false

FspI TGC^GCA JNO true false

GlaI GC^GC I false false

GluI GC^NGC I false false

GsaI CCCAGC(-1/-5) I false false

GsuI CTGGAG(16/14) F false false

HaeII RGCGC^Y JKNOR true false

HaeIII GG^CC BHIJKMNOQRSUXY true false

HapII C^CGG K true false

HgaI GACGC(5/10) IN true false

HhaI GCG^C BFJKNOQRUXY true false

Hin1I GR^CGYC FKO false false

Hin1II CATG^ F false false

Hin4I (8/13)GAYNNNNNVTC(13/8) F false false

Hin6I G^CGC F false false

HinP1I G^CGC N true false

HincII GTY^RAC BFHJKNOQRUXY true false

HindII GTY^RAC IMV true false

HindIII A^AGCTT BCFHIJKMNOQRSUVXY true false

HinfI G^ANTC BCFHIJKMNOQRUVXY true false

HpaI GTT^AAC BCHIJKMNOQRSUVX true false

HpaII C^CGG BFIMNOQRSUVX true false

HphI GGTGA(8/7) FN true false

Hpy166II GTN^NAC N false false

Hpy188I TCN^GA N true false

Hpy188III TC^NNGA N false false

Hpy8I GTN^NAC F true false

Hpy99I CGWCG^ N true false

HpyAV CCTTC(6/5) N true false

HpyCH4III ACN^GT N false false

HpyCH4IV A^CGT N true false

HpyCH4V TG^CA N false false

HpyF10VI GCNNNNN^NNGC F false false

HpyF3I C^TNAG F false false

Hsp92I GR^CGYC R false false

Hsp92II CATG^ R false false

HspAI G^CGC IV true false

KasI G^GCGCC N true false

KflI GG^GWCCC F false false

Kpn2I T^CCGGA F true false

KpnI GGTAC^C BCFHIJKMNOQRSUVXY true false

KroI G^CCGGC I false false

Ksp22I T^GATCA IV false false

KspAI GTT^AAC F false false

KspI CCGC^GG MS false false

Kzo9I ^GATC I false false

LguI GCTCTTC(1/4) F false false

LpnPI CCDG(10/14) N false false

Lsp1109I GCAGC(8/12) F true false

LweI GCATC(5/9) F false false

MabI A^CCWGGT I false false

MaeI C^TAG M false false

MaeII A^CGT M false false

MaeIII ^GTNAC M false false

MalI GA^TC I false false

MauBI CG^CGCGCG F false false

MbiI CCGCTC(-3/-3) F false false

MboI ^GATC BCFKNQRUXY true false

MboII GAAGA(8/7) FIJKNOQRVX true false

MfeI C^AATTG IN true false

MflI R^GATCY K false false

MhlI GDGCH^C IV false false

MlsI TGG^CCA F false false

MluCI ^AATT N false false

MluI A^CGCGT BFHIJKMNOQRUVX true false

MluNI TGG^CCA M false false

Mly113I GG^CGCC I false false

MlyI GAGTC(5/5) N true false

MmeI TCCRAC(20/18) N true false

MnlI CCTC(7/6) FINQVX true false

Mph1103I ATGCA^T F false false

MreI CG^CCGGCG F false false

MroI T^CCGGA MO false false

MroNI G^CCGGC IV false false

MroXI GAANN^NNTTC IV false false

MscI TGG^CCA BNO false false

MseI T^TAA BN true false

MslI CAYNN^NNRTG N false false

Msp20I TGG^CCA IV false false

MspA1I CMG^CKG INRV true false

MspCI C^TTAAG C false false

MspI C^CGG FHIJKMNOQRSUVXY true false

MspJI CNNR(9/13) N false false

MspR9I CC^NGG I false false

MssI GTTT^AAAC F false false

MunI C^AATTG FKM true false

Mva1269I GAATGC(1/-1) F false false

MvaI CC^WGG FMOS true false

MvnI CG^CG M false false

MvrI CGAT^CG U false false

MwoI GCNNNNN^NNGC N true false

NaeI GCC^GGC CKMNOU true false

NarI GG^CGCC JMNOQRUX false false

NciI CC^SGG JNOR false false

NcoI C^CATGG BCFHJKMNOQRSUXY true false

NdeI CA^TATG BFJKMNQRSXY true false

NdeII ^GATC JMR false false

NgoMIV G^CCGGC N true false

NheI G^CTAGC BCFJKMNOQRSUX false false

NlaIII CATG^ N true false

NlaIV GGN^NCC N true false

NmeAIII GCCGAG(21/19) N true false

NmuCI ^GTSAC F false false

NotI GC^GGCCGC BCFHJKMNOQRSUXY true false

NruI TCG^CGA BCIJKMNOQRUX false false

NsbI TGC^GCA FK false false

NsiI ATGCA^T BHJMNQRSUX false false

NspI RCATG^Y MN true false

NspV TT^CGAA JO false false

OliI CACNN^NNGTG F false false

PacI TTAAT^TAA FNO false false

PaeI GCATG^C F false false

PaeR7I C^TCGAG N true false

PagI T^CATGA F false false

PalAI GG^CGCGCC I false false

PasI CC^CWGGG F false false

PauI G^CGCGC F false false

PceI AGG^CCT IV false false

PciI A^CATGT IN false false

PciSI GCTCTTC(1/4) I false false

PcsI WCGNNNN^NNNCGW I false false

PctI GAATGC(1/-1) IV false false

PdiI GCC^GGC F false false

PdmI GAANN^NNTTC F false false

PfeI G^AWTC F false false

Pfl23II C^GTACG F false false

PflFI GACN^NNGTC N false false

PflMI CCANNNN^NTGG N false false

PfoI T^CCNGGA F false false

PhoI GG^CC N true false

PinAI A^CCGGT BMQX false false

Ple19I CGAT^CG I false false

PleI GAGTC(4/5) N false false

PmaCI CAC^GTG K false false

PmeI GTTT^AAAC N false false

PmlI CAC^GTG N false false

PpiI (7/12)GAACNNNNNCTC(13/8) F true false

PpsI GAGTC(4/5) I false false

Ppu21I YAC^GTR F false false

PpuMI RG^GWCCY NO true false

PscI A^CATGT F false false

PshAI GACNN^NNGTC KN true false

PshBI AT^TAAT K false false

PsiI TTA^TAA INO false false

Psp124BI GAGCT^C IV false false

Psp1406I AA^CGTT FK false false

Psp5II RG^GWCCY F false false

Psp6I ^CCWGG I false false

PspCI CAC^GTG IV false false

PspEI G^GTNACC IV false false

PspGI ^CCWGG N true false

PspLI C^GTACG I false false

PspN4I GGN^NCC I false false

PspOMI G^GGCCC INV false false

PspPI G^GNCC C true false

PspPPI RG^GWCCY I false false

PspXI VC^TCGAGB IN false false

PsrI (7/12)GAACNNNNNNTAC(12/7) I false false

PstI CTGCA^G BCFHIJKMNOQRSUVXY true false

PstNI CAGNNN^CTG I false false

PsuI R^GATCY F false false

PsyI GACN^NNGTC F false false

PteI G^CGCGC F false false

PvuI CGAT^CG BFKMNOQRSUXY false false

PvuII CAG^CTG BCFHIJKMNOQRSUVXY true false

RcaI T^CATGA M false false

RgaI GCGAT^CGC I false false

RigI GGCCGG^CC I false false

RruI TCG^CGA F false false

RsaI GT^AC BCFHIJMNOQRSVXY true false

RsaNI G^TAC I false false

RseI CAYNN^NNRTG F false false

Rsr2I CG^GWCCG IV false false

RsrII CG^GWCCG MNQX true false

SacI GAGCT^C FHJKMNOQRSUX true false

SacII CCGC^GG HJKNOQRX true false

SalI G^TCGAC BCFHIJKMNOQRSUVXY true false

SapI GCTCTTC(1/4) N false false

SaqAI T^TAA F false false

SatI GC^NGC F false false

Sau3AI ^GATC CHJKMNORSU true false

Sau96I G^GNCC JNOU true false

SbfI CCTGCA^GG INOV false false

ScaI AGT^ACT BCFJKMNOQRSX true false

SchI GAGTC(5/5) F false false

ScrFI CC^NGG JNO true false

SdaI CCTGCA^GG F false false

SduI GDGCH^C F false false

SetI ASST^ I false false

SexAI A^CCWGGT MN true false

SfaAI GCGAT^CGC F false false

SfaNI GCATC(5/9) INV false false

SfcI C^TRYAG N false false

SfiI GGCCNNNN^NGGCC CFIJKMNOQRSUVX true false

SfoI GGC^GCC N true false

Sfr274I C^TCGAG IV false false

Sfr303I CCGC^GG IV false false

SfuI TT^CGAA M false false

SgeI CNNGNNNNNNNNN^ F false false

SgfI GCGAT^CGC R false false

SgrAI CR^CCGGYG MN true false

SgrBI CCGC^GG C false false

SgrDI CG^TCGACG F false false

SgsI GG^CGCGCC F false false

SinI G^GWCC X true false

SlaI C^TCGAG C false false

SmaI CCC^GGG BCFHIJKMNOQRSUVXY true false

SmiI ATTT^AAAT FIKV false false

SmiMI CAYNN^NNRTG IV false false

SmlI C^TYRAG N false false

SmoI C^TYRAG F false false

SmuI CCCGC(4/6) F false false

SnaBI TAC^GTA CKMNR true false

SpeI A^CTAGT BHJKMNOQRSUX false false

SphI GCATG^C BCHIJKMNOQRSVX false false

SrfI GCCC^GGGC EO false false

Sse8387I CCTGCA^GG K false false

Sse9I ^AATT IV true false

SseBI AGG^CCT C false false

SsiI CCGC(-3/-1) F false false

SspDI G^GCGCC F false false

SspI AAT^ATT BCFIJKMNOQRSUVX true false

SstI GAGCT^C BC false false

SstII CCGC^GG B false false

StrI C^TCGAG U false false

StuI AGG^CCT BJKMNQRUX false false

StyD4I ^CCNGG N true false

StyI C^CWWGG CJMNR false false

SwaI ATTT^AAAT JMN true false

TaaI ACN^GT F false false

TaiI ACGT^ F false false

TaqI T^CGA BCFIJKMNOQRSUVXY true false

TaqII GACCGA(11/9),CACCCA(11/9) QX true false

TasI ^AATT F false false

TatI W^GTACW F false false

TauI GCSG^C F false false

TfiI G^AWTC N true false

TliI C^TCGAG N false false

Tru1I T^TAA F false false

Tru9I T^TAA IMRV false false

TscAI CASTGNN^ F false false

TseI G^CWGC N true false

TsoI TARCCA(11/9) F false false

Tsp45I ^GTSAC N true false

Tsp509I ^AATT N true false

TspDTI ATGAA(11/9) QX false false

TspEI ^AATT O false false

TspGWI ACGGA(11/9) QX true false

TspMI C^CCGGG N true false

TspRI CASTGNN^ N true false

TstI (8/13)CACNNNNNNTCC(12/7) F true false

Tth111I GACN^NNGTC IKNQRVX true false

Van91I CCANNNN^NTGG FKM false false

Vha464I C^TTAAG V false false

VneI G^TGCAC IV false false

VpaK11BI G^GWCC K false false

VspI AT^TAAT FIRV true false

XagI CCTNN^NNNAGG F false false

XapI R^AATTY F false false

XbaI T^CTAGA BCFHIJKMNOQRSUVXY true false

XceI RCATG^Y F false false

XcmI CCANNNNN^NNNNTGG N true false

XhoI C^TCGAG BFHJKMNOQRSUXY true false

XhoII R^GATCY R true false

XmaI C^CCGGG INRUV true false

XmaJI C^CTAGG F false false

XmiI GT^MKAC F false false

XmnI GAANN^NNTTC NRU false false

XspI C^TAG K false false

ZraI GAC^GTC INV false false

ZrmI AGT^ACT I false false

Zsp2I ATGCA^T IV false false

-

+Not purchasable

AaaI C^GGCCG false false

AagI AT^CGAT false false

AauI T^GTACA false false

AbaI T^GATCA false false

AbeI CCTCAGC(-5/-2) false false

AbrI C^TCGAG false false

Acc113I AGT^ACT false false

AccB2I RGCGC^Y false false

AccEBI G^GATCC false false

AceI G^CWGC false false

AceII GCTAG^C false false

AceIII CAGCTC(7/11) false false

AclNI A^CTAGT false false

AcpI TT^CGAA false false

AcpII CCANNNN^NTGG false false

AcrII G^GTNACC false false

AeuI CC^WGG false false

Afa16RI CGAT^CG false false

Afa22MI CGAT^CG true false

AflI G^GWCC false false

AglI CC^WGG false false

AhaB8I G^GTACC false false

AhaI CC^SGG false false

AhaII GR^CGYC false false

AhaIII TTT^AAA false false

AhyI C^CCGGG false false

AitI AGC^GCT false false

AjoI CTGCA^G false false

AliAJI CTGCA^G false false

AliI G^GATCC false false

AlwXI GCAGC(8/12) false false

AocI CC^TNAGG false false

AocII GDGCH^C false false

AorI CC^WGG false false

AosI TGC^GCA false false

AosII GR^CGYC false false

AoxI ^GGCC false false

ApaBI GCANNNNN^TGC false false

ApaCI G^GATCC false false

ApaORI CC^WGG false false

ApiI CTGCA^G false false

ApyI CC^WGG false false

ApyPI ATCGAC(20/18) true false

AquI C^YCGRG true false

AquII GCCGNAC(20/18) false false

AquIII GAGGAG(20/18) false false

AquIV GRGGAAG(19/17) false false

AseII CC^SGG false false

Asi256I G^ATC false false

Asi372I ATGCA^T false false

AsiAI A^CCGGT false false

AsiI G^GATCC false false

AsnI AT^TAAT false false

Asp10HI TT^CGAA false false

Asp10HII CCANNNN^NTGG false false

Asp26HI GAATGC(1/-1) false false

Asp27HI GAATGC(1/-1) false false

Asp35HI GAATGC(1/-1) false false

Asp36HI GAATGC(1/-1) false false

Asp40HI GAATGC(1/-1) false false

Asp50HI GAATGC(1/-1) false false

Asp713I CTGCA^G false false

Asp745I G^GWCC false false

AspAI G^GTNACC false false

AspBHI YSCNS(8/12) false false

AspHI GWGCW^C false false

AspMDI ^GATC false false

AspMI AGG^CCT false false

AspNI GGN^NCC false false

AstWI GR^CGYC false false

AsuI G^GNCC false false

AsuIII GR^CGYC false false

AtsI GACN^NNGTC false false

AvcI G^GNCC false false

AvoI RCATG^Y false false

AvrBII C^CTAGG false false

Bac36I G^GNCC false false

Bal228I G^GNCC false false

BamGI CAG^CTG false false

BamNxI G^GWCC false false

BanAI GG^CC false false

BavAI CAG^CTG false false

BavAII G^GNCC false false

BavBI CAG^CTG false false

BavBII G^GNCC false false

BavCI AT^CGAT false false

BavI CAG^CTG false false

BbeI GGCGC^C false false

Bbi24I A^CGCGT false false

BbiII GR^CGYC false false

Bbr7I GAAGAC(7/11) false false

BbrI A^AGCTT false false

BbtI G^CGC false false

Bbv16II GAAGAC(2/6) false false

BbvAI GAANN^NNTTC false false

BbvAII AT^CGAT false false

BbvAIII T^CCGGA false false

BbvBI G^GYRCC false false

BbvII GAAGAC(2/6) false false

Bca77I W^CCGGW false false

Bce22I G^GNCC false false

Bce243I ^GATC false false

Bce4I GCNNNNN^NNGC false false

Bce751I G^GATCC false false

Bce83I CTTGAG(16/14) false false

BceBI CG^CG false false

BceCI GCNNNNN^NNGC false false

BceSIII ACGGC(12/14) false false

BcefI ACGGC(12/13) false false

Bci29I AT^CGAT false false

BciBI AT^CGAT false false

BciBII CC^WGG false false

BcmI AT^CGAT false false

Bco116I CTCTTC(1/4) false false

Bco118I R^CCGGY false false

Bco27I C^CGG false false

Bco5I CTCTTC(1/4) false false

BcoAI CAC^GTG false false

BcoI C^YCGRG false false

BcoKI CTCTTC(1/4) true false

BcuAI G^GWCC false false

BdiI AT^CGAT false false

BdiSI C^TRYAG false false

BecAII GG^CC false false

BepI CG^CG true false

BetI W^CCGGW false false

Bfi57I ^GATC false false

Bfi89I Y^GGCCR false false

BflI CCNNNNN^NNGG false false

BfrBI ATGCA^T false false

BfuAII GCATG^C false false

BgiI GACN^NNGTC false false

Bim19I TT^CGAA false false

Bim19II GG^CC false false

BimI TT^CGAA false false

BinI GGATC(4/5) false false

BinSII GGCGC^C false false

BlfI T^CCGGA false false

Bli41I AT^CGAT false false

Bli736I GGTCTC(1/5) false false

Bli86I AT^CGAT false false

BliAI AT^CGAT false false

BliHKI CC^TNAGG false false

BliRI AT^CGAT false false

BloHI R^GATCY false false

BloHII CTGCA^G false false

BluI C^TCGAG false false

BmaHI GAATGC(1/-1) false false

Bme12I ^GATC false false

Bme142I RGC^GCY false false

Bme1580I GKGCM^C true false

Bme216I G^GWCC false false

Bme361I GG^CC false false

Bme585I CCCGC(4/6) false false

BmpI G^GWCC false false

BmyI GDGCH^C false false

BnaI G^GATCC true false

Bpa34I AGT^ACT false false

BpcI C^TRYAG false false

BpoAI AT^TAAT false false

BptI CC^WGG false false

Bpu95I CG^CG false false

BpuAmI GAG^CTC false false

BpuB5I C^GTACG false false

BpuDI CCTNAGC(-5/-2) false false

BpuI GRGCY^C false false

BpuSI GGGAC(10/14) true false

BsaOI CGRY^CG false false

BsbI CAACAC(21/19) false false

Bsc107I CCNNNNN^NNGG false false

Bsc91I GAAGAC(2/6) false false

BscAI GCATC(4/6) false false

BscBI GGN^NCC false false

BscCI GAATGC(1/-1) false false

BscFI ^GATC false false

BscI AT^CGAT false false

Bse15I C^YCGRG false false

Bse16I CC^WGG false false

Bse17I CC^WGG false false

Bse24I CC^WGG false false

Bse634I R^CCGGY true false

Bse64I G^GTNACC false false

BseKI GCAGC(8/12) false false

BseQI GG^CC false false

BseT10I G^GTNACC false false

BseT9I G^GTNACC false false

BseZI CTCTTC(1/4) false false

Bsh1365I GATNN^NNATC false false

Bsh45I GWGCW^C false false

BshGI CC^WGG false false

BshI GG^CC false false

BshKI G^GNCC false false

BsiBI GATNN^NNATC false false

BsiCI TT^CGAA false false

BsiI CACGAG(-5/-1) false false

BsiKI G^GTNACC false false

BsiLI CC^WGG false false

BsiMI T^CCGGA false false

BsiQI T^GATCA false false

BsiXI AT^CGAT false false

BsiYI CCNNNNN^NNGG false false

BsiZI G^GNCC false false

Bsm6I GWGCW^C false false

BsmRI T^GTACA false false

BsmSI C^CWWGG false false

BsoCI GDGCH^C false false

BsoFI GC^NGC false false

BsoMAI GTCTC(1/5) false false

Bsp105I ^GATC false false

Bsp106I AT^CGAT true false

Bsp123I CG^CG false false

Bsp143II RGCGC^Y false false

Bsp153AI CAG^CTG false false

Bsp1894I G^GNCC false false

Bsp2095I ^GATC false false

Bsp211I GG^CC false false

Bsp24I (8/13)GACNNNNNNTGG(12/7) false false

Bsp4009I G^GATCC false false

Bsp423I GCAGC(8/12) false false

Bsp50I CG^CG false false

Bsp519I GRGCY^C false false

Bsp63I CTGCA^G false false

Bsp67I ^GATC false false

Bsp6I GC^NGC true false

Bsp98I G^GATCC true false

BspA2I C^CTAGG false false

BspAAI C^TCGAG false false

BspAAII T^CTAGA false false

BspAAIII G^GATCC false false

BspAI ^GATC false false

BspANI GG^CC false false

BspBI CTGCA^G false false

BspBII G^GNCC false false

BspBRI GG^CC false false

BspBS31I GAAGAC(2/6) false false

BspCI CGAT^CG false false

BspD6I GACTC(4/6) false false

BspF4I G^GNCC false false

BspFI ^GATC false false

BspIS4I GAAGAC(2/6) true false

BspJI ^GATC false false

BspJII AT^CGAT false false

BspKI GG^CC false false

BspKMI ^GATC false false

BspKT5I CTGAAG(16/14) false false

BspKT6I GAT^C true false

BspKT8I A^AGCTT false false

BspLAI GCG^C false false

BspLAII TT^CGAA false false

BspLAIII A^AGCTT false false

BspLS2I GDGCH^C false false

BspLU11I A^CATGT false false

BspLU11III GGGAC(10/14) true false

BspLU4I C^YCGRG false false

BspM39I CAG^CTG false false

BspM90I GTA^TAC false false

BspMAI CTGCA^G false false

BspMII T^CCGGA false false

BspMKI G^TCGAC false false

BspNI CC^WGG false false

BspO4I CAG^CTG false false

BspOVI GACNNN^NNGTC false false

BspOVII AT^CGAT false false

BspR7I CC^TNAGG false false

BspRI GG^CC true false

BspST5I GCATC(5/9) true false

BspTNI GGTCTC(1/5) false false

BspTS514I GAAGAC(2/6) false false

BspUI GCSG^C false false

BspWI GCNNNNN^NNGC false false

BspXI AT^CGAT false false

BspXII T^GATCA false false

BspZEI AT^CGAT false false

BsrAI G^GWCC false false

BsrBRI GATNN^NNATC false false

BssHI C^TCGAG false false

BssIMI GGGTC(-3/0) false false

Bst100I CC^WGG false false

Bst11I ACTGG(1/-1) false false

Bst12I GCAGC(8/12) false false

Bst19I GCATC(4/6) true false

Bst19II ^GATC false false

Bst1I CC^WGG false false

Bst22I CCNNNNN^NNGG false false

Bst28I AT^CGAT false false

Bst2I CC^WGG false false

Bst31NI CCGCTC(-3/-3) false false

Bst31TI GGATC(4/5) false false

Bst38I CC^WGG false false

Bst40I C^CGG false false

Bst71I GCAGC(8/12) false false

BstB7SI R^CCGGY false false

BstBS32I GAAGAC(2/6) false false

BstBSI GTA^TAC false false

BstBZ153I G^CGCGC false false

BstD102I CCGCTC(-3/-3) false false

BstENII ^GATC false false

BstEZ359I GTT^AAC false false

BstFI A^AGCTT false false

BstFZ438I CCCGC(4/6) false false

BstGZ53I CGTCTC(1/5) false false

BstH9I GGATC(4/5) false false

BstHPI GTT^AAC false false

BstHZ55I CCANNNNN^NTGG false false

BstI G^GATCC true false

BstIZ316I CACNNN^GTG false false

BstJZ301I C^TNAG false false

BstM6I CC^WGG false false

BstMZ611I ^CCNGG false false

BstNZ169I AT^CGAT false false

BstOZ616I GGGAC(10/14) false false

BstPZ740I C^TTAAG false false

BstRZ246I ATTT^AAAT false false

BstSI C^YCGRG false false

BstSWI ATTT^AAAT false false

BstT10I G^GTNACC false false

BstT7I T^GATCA false false

BstT9I G^GTNACC false false

BstTS5I GAAGAC(2/6) false false

BstVI C^TCGAG true false

Bsu1532I CG^CG false false

Bsu1854I GRGCY^C false false

Bsu23I T^CCGGA false false

Bsu537I GGTCTC(1/5) false false

Bsu54I G^GNCC false false

Bsu6I CTCTTC(1/4) false false

BsuBI CTGCA^G true false

BsuFI C^CGG true false

BteI GG^CC false false

BthAI G^GWCC false false

BthCI GCNG^C true false

BthDI CC^WGG false false

BthEI CC^WGG false false

BthP35I C^TRYAG false false

BtkI CG^CG false false

BtkII ^GATC false false

BvuBI C^GTACG false false

BvuI GRGCY^C false false

CacI ^GATC false false

CatHI CTCTTC(1/4) false false

CauB3I T^CCGGA false false

CauI G^GWCC false false

CauII CC^SGG false false

CbiI TT^CGAA false false

CboI C^CGG true false

CbrI CC^WGG false false

CchI C^TAG false false

CchII GGARGA(11/9) true false

CchIII CCCAAG(20/18) true false

CcoI GCC^GGC false false

CcrI C^TCGAG false false

CcuI G^GNCC false false

CcyI ^GATC false false

CdiI CATCG(-1/-1) false false

CdpI GCGGAG(20/18) true false

CelI G^GATCC false false

CeqI GAT^ATC false false

CfaI R^AATTY false false

CflI CTGCA^G false false

Cfr6I CAG^CTG true false

CfrA4I CTGCA^G false false

CfrBI C^CWWGG true false

CfrJ4I CCC^GGG false false

CfuI GA^TC false false

CfuII CTGCA^G false false

ChaI GATC^ false false

CjeI (8/14)CCANNNNNNGT(15/9) true false

CjePI (7/13)CCANNNNNNNTC(14/8) false false

CltI GG^CC false false

CpfI ^GATC false false

CscI CCGC^GG false false

CsiAI A^CCGGT false false

CsiBI GC^GGCCGC false false

Csp68KI G^GWCC true false

Csp68KII TT^CGAA false false

Csp68KIII ATGCA^T false false

Csp68KVI CG^CG false false

CspBI GC^GGCCGC false false

CstI CTGCA^G false false

CstMI AAGGAG(20/18) true false

CthII CC^WGG false false

CviAI ^GATC true false

CviBI G^ANTC true false

CviKI RG^CY true false

CviRI TG^CA true false

CviRII G^TAC true false

CvnI CC^TNAGG false false

DmaI CAG^CTG false false

DpaI AGT^ACT false false

DraRI CAAGNAC(20/18) true false

DrdIV TACGAC(20/18) true false

DsaI C^CRYGG false false

DsaII GG^CC false false

DsaIII R^GATCY false false

DsaIV G^GWCC false false

DsaV ^CCNGG true false

EacI GGATC(4/5) false false

Eae46I CCGC^GG false false

EaeAI C^CCGGG false false

EagBI CGAT^CG false false

EagMI G^GWCC false false

EcaI G^GTNACC true false

Eci125I G^GTNACC false false

Ecl18kI ^CCNGG true false

Ecl2zI CTGCA^G false false

Ecl37kI CTGCA^G false false

EclHKI GACNNN^NNGTC false false

EclI CAG^CTG false false

EclRI C^CCGGG false false

Eco137kI ^CCNGG false false

Eco13kI ^CCNGG false false

Eco1524I AGG^CCT false false

Eco1831I ^CCSGG true false

Eco21kI ^CCNGG false false

Eco255I AGT^ACT false false

Eco27kI C^YCGRG false false

Eco29kI CCGC^GG true false

Eco56I G^CCGGC true false

Eco64I G^GYRCC false false

Eco75KI GRGCY^C false false

Eco78I GGC^GCC false false

EcoA4I GGTCTC(1/5) false false

EcoHI ^CCSGG true false

EcoHK31I Y^GGCCR true false

EcoO128I G^GTNACC false false

EcoO44I GGTCTC(1/5) false false

EcoVIII A^AGCTT true false

ErhB9I CGAT^CG false false

ErhB9II C^CWWGG false false

ErpI G^GWCC false false

EsaBC3I TC^GA true false

EsaBC4I GG^CC true false

EscI C^TCGAG false false

Esp1396I CCANNNN^NTGG true false

Esp4I C^TTAAG false false

EspI GC^TNAGC false false

FalII CG^CG false false

FauBII CG^CG false false

FbrI GC^NGC false false

FdiI G^GWCC false false

FdiII TGC^GCA false false

FgoI C^TAG false false

FmuI GGNC^C false false

FnuAI G^ANTC false false

FnuCI ^GATC false false

FnuDI GG^CC true false

FnuDII CG^CG true false

FnuDIII GCG^C false false

FnuEI ^GATC false false

FsiI R^AATTY false false

Fsp1604I CC^WGG false false

FspII TT^CGAA false false

FspMSI G^GWCC false false

FssI G^GWCC true false

FunI AGC^GCT false false

FunII G^AATTC false false

GalI CCGC^GG false false

GceGLI CCGC^GG false false

GceI CCGC^GG false false

GdiI AGG^CCT false false

GdiII CGGCCR(-5/-1) false false

GstI G^GATCC false false

HacI ^GATC false false

HaeI WGG^CCW false false

HaeIV (7/13)GAYNNNNNRTC(14/9) true false

HalI G^AATTC false false

HalII CTGCA^G false false

HgiAI GWGCW^C false false

HgiBI G^GWCC true false

HgiCI G^GYRCC true false

HgiCII G^GWCC true false

HgiCIII G^TCGAC false false

HgiDI GR^CGYC true false

HgiDII G^TCGAC true false

HgiEI G^GWCC true false

HgiGI GR^CGYC true false

HgiHI G^GYRCC false false

HgiHII GR^CGYC false false

HgiHIII G^GWCC false false

HgiI GR^CGYC false false

HgiJI G^GWCC false false

HgiJII GRGCY^C false false

HgiS22I CC^SGG false false

HhaII G^ANTC true false

Hin2I C^CGG false false

Hin4II CCTTC(6/5) true false

HinJCI GTY^RAC false false

HjaI GAT^ATC false false

Hpy178III TC^NNGA false false

Hpy51I ^GTSAC false false

HpyBI GT^AC false false

HpyBII GTN^NAC false false

HpyC1I CCATC(4/5) false false

HpyCH4I CATG^ false false

HpyCI GAT^ATC false false

HpyF44III TG^CA false false

HsoI G^CGC false false

HsuI A^AGCTT false false

ItaI GC^NGC false false

Kaz48kI RGGNC^CY false false

KoxII GRGCY^C false false

Kpn2kI ^CCNGG true false

Kpn378I CCGC^GG false false

Kpn49kI G^AATTC false false

Kpn49kII ^CCSGG false false

Ksp632I CTCTTC(1/4) false false

Kzo49I G^GWCC false false

LcaI AT^CGAT false false

LlaAI ^GATC false false

LlaBI C^TRYAG false false

LlaCI A^AGCTT true false

LlaG2I G^CTAGC false false

Lmu60I CC^TNAGG false false

LplI AT^CGAT false false

LpnI RGC^GCY false false

LspI TT^CGAA false false

Mae7806III G^GWCC true false

MaeK81I C^GTACG false false

MaeK81II G^GNCC false false

MamI GATNN^NNATC true false

MaqI CRTTGAC(21/19) true false

MavI C^TCGAG false false

McaTI GCGC^GC true false

MchAI GC^GGCCGC false false

MchAII GG^CC false false

MchI GG^CGCC false false

McrI CGRY^CG false false

MfoAI GG^CC false false

Mgl14481I CC^SGG false false

MgoI ^GATC false false

MhaAI CTGCA^G false false

MkrAI ^GATC false false

MlaAI C^TCGAG false false

MlaI TT^CGAA false false

MltI AG^CT false false

Mlu23I G^GATCC false false

Mlu31I TGG^CCA false false

MluB2I TCG^CGA false false

MnoI C^CGG false false

Mpr154I CCGC^GG false false

Msp17I GR^CGYC false false

Msp67I CC^NGG false false

MspB4I G^GYRCC false false

MspSWI ATTT^AAAT false false

MspV281I GWGCW^C false false

MspYI YAC^GTR false false

MstI TGC^GCA false false

MstII CC^TNAGG false false

MthZI C^TAG true false

MxaI GAG^CTC false false

NblI CGAT^CG false false

NcrI A^GATCT false false

NcuI GAAGA(8/7) true false

NdaI GG^CGCC false false

NgoAIII CCGC^GG true false

NgoAIV G^CCGGC true false

NgoAVIII (12/14)GACNNNNNTGA(13/11) true false

NgoPII GG^CC true false

NgoPIII CCGC^GG false false

NlaCI CATCAC(19/17) true false

NlaII ^GATC false false

Nli3877I CYCGR^G false false

NmeCI ^GATC false false

NmeDI (12/7)RCCGGY(7/12) true false

NmeRI CAG^CTG false false

NopI G^TCGAC false false

NphI ^GATC false false

NruGI GACNNN^NNGTC false false

NsiCI GAT^ATC false false

Nsp29132II G^GATCC false false

Nsp7121I G^GNCC false false

NspBII CMG^CKG false false

NspHI RCATG^Y true false

NspII GDGCH^C false false

NspIII C^YCGRG true false

NspIV G^GNCC false false

NspLKI GG^CC false false

NspMACI A^GATCT false false

NspSAI C^YCGRG false false

NspSAII G^GTNACC false false

NspSAIV G^GATCC false false

NunII GG^CGCC false false

OfoI C^YCGRG false false

OkrAI G^GATCC false false

OxaNI CC^TNAGG false false

PabI GTA^C true false

Pac25I C^CCGGG true false

Pae14kI CCGC^GG false false

Pae17kI CAG^CTG false false

Pae18kI A^GATCT false false

Pae2kI A^GATCT false false

Pae5kI CCGC^GG false false

PaeAI CCGC^GG false false

PaeBI CCC^GGG false false

PaeHI GRGCY^C false false

PaePI CTGCA^G false false

PaeQI CCGC^GG false false

PalI GG^CC false false

PamI TGC^GCA false false

PamII GR^CGYC false false

PanI C^TCGAG false false

ParI T^GATCA false false

PauAI RCATG^Y false false

PauAII TTT^AAA false false

PbrTI ^GATC false false

Pde12I G^GNCC false false

Pde133I GG^CC false false

Pde137I C^CGG false false

PfaAI G^GYRCC false false

PfaAII CA^TATG false false

PfaAIII GCATG^C false false

Pfl21I CTGCA^G false false

Pfl27I RG^GWCCY false false

Pfl8I G^GATCC false false

PflBI CCANNNN^NTGG false false

PflKI GG^CC false false

PgaI AT^CGAT false false

PhaI GCATC(5/9) false false

PinBI ATGCA^T false false

PinBII T^CCGGA false false

PlaAI C^YCGRG false false

PlaAII GT^AC false false

PlaDI CATCAG(21/19) true false

PlaI GG^CC false false

PlaII TT^CGAA false false

PluTI GGCGC^C true false

Pme55I AGG^CCT false false

PpaAI TT^CGAA false false

PpaAII T^CGA false false

PpeI GGGCC^C false false

Ppu10I A^TGCAT false false

Ppu111I G^AATTC false false

PpuAI C^GTACG false false

PpuXI RG^GWCCY false false

Pru2I GG^CC false false

Psp03I GGWC^C false false

Psp1009I GCCNNNN^NGGC false false

Psp23I CTGCA^G false false

PspAI C^CCGGG false false

PspALI CCC^GGG false false

PspOMII CGCCCAR(20/18) true false

PspPRI CCYCAG(15/13) true false

PssI RGGNC^CY false false

PstNHI G^CTAGC false false

Psu161I CGAT^CG false false

PsuAI YAC^GTR false false

PtaI T^CCGGA false false

Pun14627I TGC^GCA false false

Pun14627II CAG^CTG false false

PunAI C^YCGRG false false

PunAII RCATG^Y false false

Pvu84II CAG^CTG false false

RalF40I ^GATC false false

RaqI CCGC^GG false false

RceI CATCGAC(20/18) true false

RdeGBII ACCCAG(20/18) true false

RdeGBIII (9/11)TGRYCA(11/9) true false

RflFI G^TCGAC false false

RflFII AGT^ACT false false

RleAI CCCACA(12/9) false false

RmaI C^TAG false false

Rme21I AT^CGAT false false

RpaB5I CGRGGAC(20/18) true false

RpaBI CCCGCAG(20/18) true false

RpaI GTYGGAG(11/9) true false

Rrh4273I G^TCGAC true false

RrhJ1I GCC^GGC true false

RshI CGAT^CG true false

RspLKI GCATG^C false false

RspLKII G^GATCC false false

RspXI T^CATGA false false

RsrI G^AATTC true false

Rtr63I G^TCGAC false false

RtrI G^TCGAC false false

SacNI GRGCY^C false false

Sag16I CTGCA^G false false

Sag23I CTGCA^G false false

SalPI CTGCA^G false false

SanDI GG^GWCCC false false

SarI AGG^CCT false false

Sau3239I C^TCGAG true false

SauBMKI GCC^GGC false false

SauHPI GCC^GGC false false

SauI CC^TNAGG false false

SauLPI GCC^GGC true false

SauLPII C^TCGAG false false

SauMI ^GATC false false

SauNI GCC^GGC false false

SauSI GCC^GGC false false

Sbi68I C^TCGAG false false

Sbo13I TCG^CGA false false

SbvI GG^CC false false

SceIII G^CCGGC false false

SchZI CCGC^GG false false

SciI CTC^GAG false false

SciNI G^CGC false false

SdeAI CAGRAG(21/19) true false

SdeOSI (11/13)GACNNNNRTGA(12/10) true false

SdiI GGCCNNNN^NGGCC false false

SecI C^CNNGG false false

SelI ^CGCG false false

SenPT14bI CCGC^GG false false

SenPT16I C^GGCCG false false

SepI ATGCA^T false false

SexBI CCGC^GG false false

SexCI CCGC^GG false false

SfaI GG^CC false false

SfeI C^TRYAG false false

SflI CTGCA^G false false

SgrTI CCDS(10/14) false false

SimI GGGTC(-3/0) false false

SleI ^CCWGG false false

Slu1777I GCC^GGC false false

SmuEI G^GWCC false false

SniI CC^WGG false false

SnoI G^TGCAC false false

Sol10179I C^TCGAG false false

SolI G^GATCC false false

SpaHI GCATG^C false false

SplI C^GTACG false false

SpmI AT^CGAT false false

SpoI TCG^CGA false false

SpuI CCGC^GG false false

Srl32DII G^AATTC false false

Srl55DI G^AATTC false false

Srl56DI C^TRYAG false false

Srl5DI CTGCA^G false false

SrlI G^CCGGC false false

Sru30DI AGG^CCT false false

Sru4DI AT^TAAT false false

SruI TTT^AAA false false

SsbI A^AGCTT false false

SscL1I G^ANTC true false

Sse1825I GG^GWCCC false false

Sse232I CG^CCGGCG false false

Sse8647I AG^GWCCT false false

SseAI GG^CGCC false false

SshAI CC^TNAGG false false

SsiAI ^GATC false false

SsiBI ^GATC false false

SslI CC^WGG false false

SsoI G^AATTC true false

SsoII ^CCNGG true false

Ssp1I TT^CGAA false false

Ssp27144I AT^CGAT false false

Ssp4800I T^GTACA false false

Ssp5230I GACGT^C false false

SspAI ^CCWGG false false

SspBI T^GTACA false false

SspCI GCC^GGC false false

SspD5I GGTGA(8/8) false false

SspD5II ATGCA^T false false

SspRFI TT^CGAA false false

SsrI GTT^AAC false false

Sst12I CTGCA^G false false

SstE37I CGAAGAC(20/18) true false

SteI AGG^CCT false false

Sth117I CC^WGG false false

Sth132I CCCG(4/8) false false

Sth134I C^CGG false false

Sth302I CC^WGG false false

Sth302II CC^GG false false

Sth368I ^GATC true false

SthI G^GTACC false false

StsI GGATG(10/14) true false

SuaI GG^CC true false

SuiI G^CWGC false false

SunI C^GTACG false false

SurI G^GATCC false false

SviI TT^CGAA false false

Taq52I G^CWGC false false

TaqXI CC^WGG false false

TelI GACN^NNGTC false false

ThaI CG^CG true false

Tru201I R^GATCY false false

TscI ACGT^ false false

Tsp1I ACTGG(1/-1) false false

Tsp32I T^CGA false false

Tsp32II T^CGA false false

Tsp49I ACGT^ false false

Tsp4CI ACN^GT false false

Tsp8EI GCCNNNN^NGGC false false

TspBI C^CRYGG false false

Tth111II CAARCA(11/9) true false

TthHB8I T^CGA true false

Tvu2HI GG^CC false false

Uba153AI CAG^CTG false false

Uba4009I G^GATCC false false

UbaM39I CAG^CTG false false

UcoMSI (7/5)GAGCTC(5/7) false false

UnbI ^GGNCC false false

Uur960I GC^NGC false false

VpaK11AI ^GGWCC false false

VpaK32I GCTCTTC(1/4) false false

WviI CACRAG(21/19) true false

XcaI GTA^TAC false false

XciI G^TCGAC false false

XcpI CTGCA^G false false

XcyI C^CCGGG true false

XmaCI C^CCGGG false false

XmaIII C^GGCCG true false

XorII CGAT^CG true false

XpaI C^TCGAG false false

YenI CTGCA^G false false

ZanI CC^WGG false false

ZhoI AT^CGAT false false

-

-

-
