# Supplementary figures and images for: CisSERS: Customizable In Silico Sequence Evaluation for Restriction Sites
Source: PLoS One. 2016 Apr 12;11(4):e0152404. doi: 10.1371/journal.pone.0152404 (PMC4829253; doi:10.1371/journal.pone.0152404)

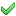

Supplement: S5 File — (ZIP) [file pone.0152404.s005.zip › CisSERS/doneOk.png]

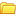

Supplement: S5 File — (ZIP) [file pone.0152404.s005.zip › CisSERS/load.png]

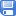

Supplement: S5 File — (ZIP) [file pone.0152404.s005.zip › CisSERS/save.png]

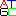

Supplement: S5 File — (ZIP) [file pone.0152404.s005.zip › CisSERS/ATGC.png]
